# Quantum Chemical and Kinetic Study on Polychlorinated Naphthalene Formation from 3-Chlorophenol Precursor

**DOI:** 10.3390/ijms160920620

**Published:** 2015-08-31

**Authors:** Fei Xu, Xiangli Shi, Qingzhu Zhang

**Affiliations:** Environment Research Institute, Shandong University, Jinan 250100, China; E-Mails: xufei@sdu.edu.cn (F.X.); 123songchuan@163.com (X.S.)

**Keywords:** 3-chlorophenol, polychlorinated naphthalene, formation mechanism, rate constants, density functional method

## Abstract

Polychlorinated naphthalenes (PCNs) are the smallest chlorinated polycyclic aromatic hydrocarbons (Cl-PAHs) and are often called dioxin-like compounds. Chlorophenols (CPs) are important precursors of PCN formation. In this paper, mechanistic and kinetic studies on the homogeneous gas-phase formation mechanism of PCNs from 3-CP precursor were investigated theoretically by using the density functional theory (DFT) method and canonical variational transition-state theory (CVT) with small curvature tunneling contribution (SCT). The reaction priority of different PCN formation pathways were disscussed. The rate constants of crucial elementary steps were deduced over a wide temperature range of 600−1200 K. The mechanisms were compared with the experimental observation and our previous works on the PCN formation from 2-CP and 4-CP. This study shows that pathways ended with Cl elimination are favored over those ended with H elimination from the 3-CP precursor. The formation potential of MCN is larger than that of DCN. The chlorine substitution pattern of monochlorophenols has a significant effect on isomer patterns and formation potential of PCN products. The results can be input into the environmental PCN controlling and prediction models as detailed parameters, which can be used to confirm the formation routes of PCNs, reduce PCN emission and establish PCN controlling strategies.

## 1. Introduction

Chlorinated polycyclic aromatic hydrocarbons (Cl-PAHs) are PAH derivatives and have recently attracted considerable public concern because of their widespread occurrence [1,2], high toxicity [3,4], and close association with particle formation in urban air [2,5,6]. Compared with their parent PAHs, Cl-PAHs have lower vapor pressures, higher octanol-water partition coefficient values, and even highter mutagenicity and toxicixty [7,8,9]. Main sources of Cl-PAHs include chlorinated tap waters, combustion of chlorine-containing materials, wastewater of pulp and paper mills, photochemical reactions of PAHs, automobile exhaust, and municipal solid waste (MSW) incineration [1,10,11,12,13,14,15,16]. Polychlorinated naphthalenes (PCNs), which are often called dioxin-like compounds, are the smallest Cl-PAHs. The mechanism of PCN formation will represent growth reactions for larger Cl-PAHs. PCN are a group of 75 possible congeners consisting of naphthanlene substituted with 1–8 chlorine atoms. The structures of PCNs are 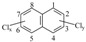.

Chlorophenols (CPs) are among the most abundant aromatic compounds found in MSW exhaust gases [17]. CPs are an important class of serious environmental contaminants because of their toxicity, carcinogenicity, bioaccumulation, and considerable persistence. Due to the different substitution pattern of phenol, CPs have 19 isomers. Among them, monochlorophenols (MCPs) are some of the most important contaminants existed in the environment which were used in several industrial processes and agricultures [18,19]. It was reported that the three MCP have similar toxicity, but 3-Chlorophenol (3-CP) has a the slowest degradation rate and the longest residence time among them [20]. 3-CP is used as the intermediate products of the coal and petroleum refinery industries, and raw materials to further synthesize pharmaceutical products, pesticides, solvents, textile additives, and specialty chemicals [21].Contact and breathing 3-CP can irritate and burn the skin, eyes, nose, throat, and lungs causing coughing, wheezing, and shortness of breath [22]. High exposure can cause headache, dizziness, fatigue, restlessness, muscle weakness, tremors, seizures, coma, and even death [22]. 3-CP is on the Hazadous Substance list of Department of Traspotation (DOT), New Jersey Department of Environmental Protection(DEP) and Environmental Protection Agency(EPA) [22].

**Scheme 1:**
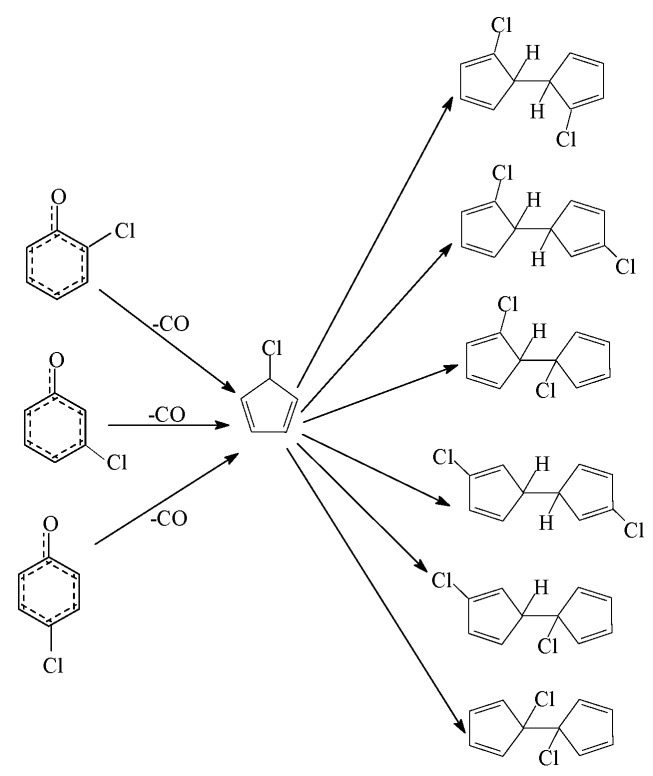
The hypothesis on chlorinated dihydrofulvene formation from three monochlorophenols.

PCN formation contains gas-phase homogeneous reactions and heterogeneous metal-mediated reactions on fly ash from precursors. Numbers studies reported that PCN are formed unintentionally from industrial thermal processes and incomplete combustion from municipal solid waste incineration as byproducts, along with other halogenated aromatic compounds, such as PCBs, PCDDs, and PCDFs in combustion exhaust gas [23,24,25,26,27,28,29,30,31]. Among different precursors, CPs play a crucial role in the formation of PCNs [26,27,28,29,32,33,34]. In combustion and thermal processes, CPs can lose phenoxyl-hydrogen to form chlorophenoxy radicals (CPRs). In 1975, Cypres and Betterns first reported the PCN formation from phenol [35]. Since that time, many researches reported the naphthalene formation from phenol via dimerization of cyclopentadienyl (CPDyl) radicals, which were produced via CO loss from phenol [36,37,38]. CPDyl radicals have been shown to play important role in the PAH growth mechanisms at high temperature [39,40]. According to this naphthalene formation mechanism, previous studies predicted PCN formation occur via condensation of two chlorinated cyclopentadienyl radicals (chloro-CPDyl 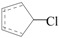) produced by CO loss of CPRs [26,27,28,29], based on the naphthalene formation mechanism proposed by Melius [41]. Recombination of two chloro-CPDyl radicals to form chlorinated dihydrofulvene (
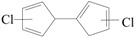
), followed by rearrangement and H/Cl elimination can result in the PCN’s formation [25,26,27,28]. In this mechanism, 2-CP, 3-CP, and 4-CP were speculated to form the same chloro-CPDyl radical and exactly the same PCN isomer patterns [26,27,28,29,32,33]. However, this hypothesis was experimentally opposed by Kim via obtaining completely different PCN isomer distributions from 2-CP, 3-CP, and 4-CP (Scheme 1) [32,33]. For example, in Kim’s experiment, 2-CP produced mostly 1-MCN and 1,5/1,6/1,7-DCNs; 4-CP produced mostly 2-MCN and 2,6/2,7-DCNs; 3-CP produced nearely equivalent 1-MCN and 2-MCN, and nearly equal 1,5/1,6/1,7-DCNs and 2,6/2,7-DCNs [32,33]. In addition, this hypothesis was, again, denied by some experimental observations in thermal processes that the correlation between PCN and PCDF isomer distributions or mass concentrations is more closer than that between PCN and PCDD, which indicate a new PCN formation mechanism more similar with PCDF formation than PCDD [30,31,42,43,44,45,46]. In this situation, Kim proposed an alternative PCN formation mechanism based on experimental results [32,33,34]; in his scheme, PCNs are formed via carbon-carbon coupling at *ortho*-sites of CPR pairs, resulting in an intermediate chlorinated o,oʹ-dihydroxybiphenyl (chloro-DOHB) [32,33,34]. Chlorinated dihydrofulvene are formed from chloro-DOHB by two CO loss and ring close steps, not by condensation of two chloro-CPDyl radicals [32,33,34]. Both PCDF and PCN can be produced from chloro-DOHB by different tautomerization steps [32,33,34].

Due to the high toxicity of PCNs and the lack of efficient detection schemes for intermediate radicals, the specific formation mechanism of PCNs from CPs remains unclear. Our previous studies have systematically investigated the homogeneous gas-phase formation mechanism of PCNs from 2-CP and 4-CP precursors [47,48]. As a ongoing study of this field, it is important to continue study the PCN formation mechanism from 3-CP in order to compare the contributions and effects of *ortho*-, *meta-*, and *para-*chlorine of monochlorophenol on the formation potential and isomer distributions of PCN products. Kim only gave a simple mechanism of PCN formation from 3-CP which loses numbers of important intermediates and can not explain some of his experimental observations [32,33]. For example, the mechanism predicted by Kim can not give reasonable explanations on the experimental results that the amont of MCN is significantly larger than that of DCN [32,33]. In addition, only trace amounts of 1,8-DCN were experimentally observed from 3-CP, whereas the 1,8-DCN formation routes are competitive with 1,5-/1,6-/1,7-DCNs in Kim’s speculated mechanism [32,33]. Moreover, Yang also studied the PCN formation from 3-CP [27]. In Yang’s study the main MCN product from 3-CP is 2-MCN, wheras in Kim’s results 3-CP produced approximately equal amounts of 1-MCN and 2-MCN [32,33]. Furthermore, Kim mentioned in his paper that H/Cl migration in the cyclopentadiene ring of chlorinated dihydrofulvene [32,33] to *ortho*-carbon occurs first before *ortho*-carbon H/Cl elimination. However, the H or Cl atoms may be abstracted directly without H or Cl migration, which need to be studied further. Thus, more detailed and specific mechanisms on PCN formation from 3-CP precursor need to be proposed to solve all the contradictions above.

This study is carried out for three objectives. The first aim of this paper is to deeply investigate the reaction mechanism on the PCN formation from 3-CP precursor, using a comprehensive quantum calculation. Quantum chemical calculation is a useful method to establish the pathway feasibility and confirm the product priority, especially for the highly toxic compounds. A second motivation for this work is to evaluate the rate constants of the elementary reactions involved in the PCN formations over a wide temperature range of 600−1200 K. The absence of the kinetic parameters prevent to further improve and optimize PCN formation models. The third purpose is to discuss the effect of the chlorine substitution pattern of CPs on isomer patterns and formation potential of PCNs.

## 2. Results and Discussion

The formation of CPRs from CPs is the initial and key step in the formation of PCNs. In combustion and thermal processes, CPRs can be produced through loss of the phenoxyl-hydrogen via unimolecular cleavage of the O-H bond or abstracted by the active radicals H, OH, O (^3^P), and Cl. The potential barriers (Δ*E*) and the reaction heats (Δ*H*) of 3-CP phenoxyl-hydrogen cleavage and abstraction by H, OH, O (^3^P), and Cl were calculated at the MPWB1K/6-311+G(3df,2p) level as follows. The H/OH abstraction data were cited by the previous studies of our group [49,50]. All the abstraction steps are strongly exothermic:

3-CP + H → 3-CPR + H_2_ Δ*E* = 12.51 kcal/mol Δ*H* = −12.94 kcal/mol
3-CP + OH → 3-CPR + H_2_O Δ*E* = 0.17 kcal/mol Δ*H* = −27.81 kcal/mol
3-CP + O(^3^P) → 3-CPR + OH Δ*E* = 8.20 kcal/mol Δ*H* = −12.04 kcal/mol
3-CP + Cl → 3-CPR + HCl Δ*E* = −5.87 kcal/mol Δ*H* = −15.66 kcal/mol

### 2.1. Formation of Chloro-Dihydrofulvene from Dimerization of 3-CPRs

**Figure 1:**
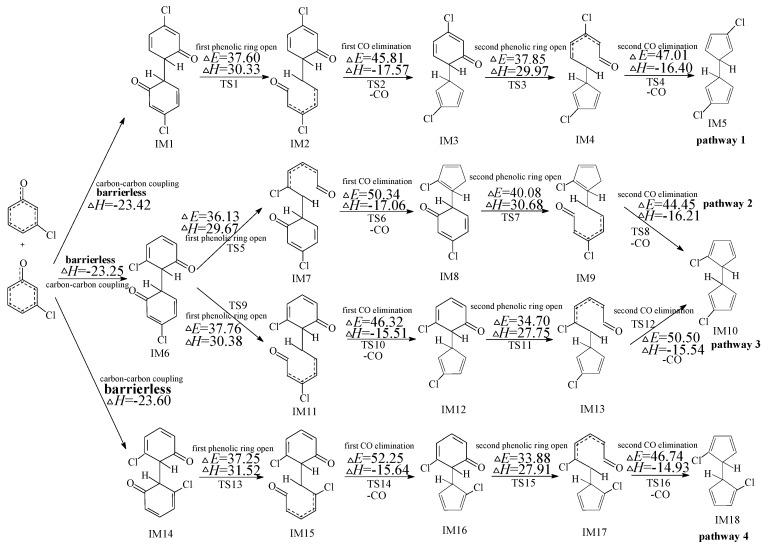
Chlorinated dihydrofulvene formation routes embedded with the potential barriers Δ*E* (in kcal/mol) and reaction heats Δ*H* (in kcal/mol) from dimerization of 3-CPRs. Δ*H* is calculated at 0 K.

**Figure 2:**
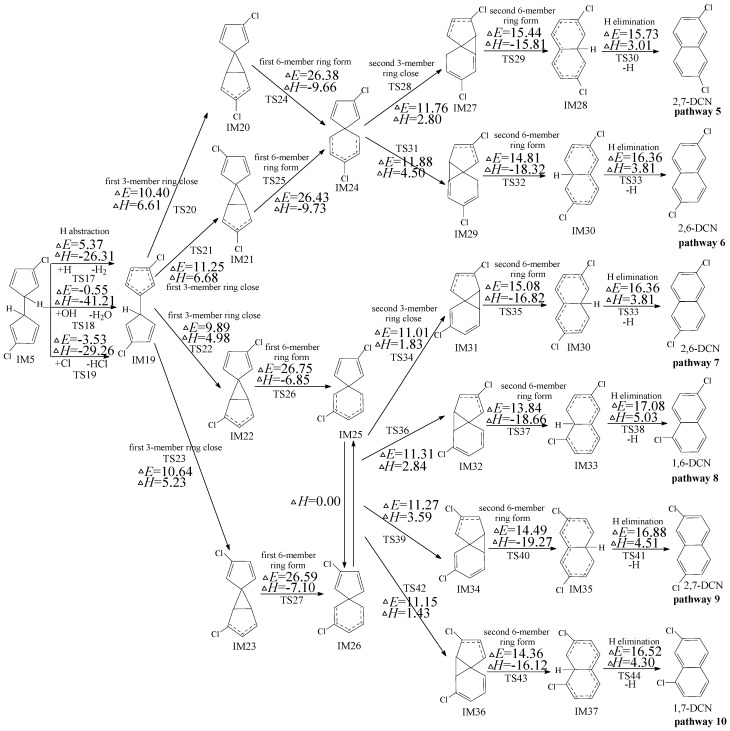
PCN formation routes embedded with the potential barriers Δ*E* (in kcal/mol) and reaction heats Δ*H* (in kcal/mol) from IM5. Δ*H* is calculated at 0 K.

**Figure 3:**
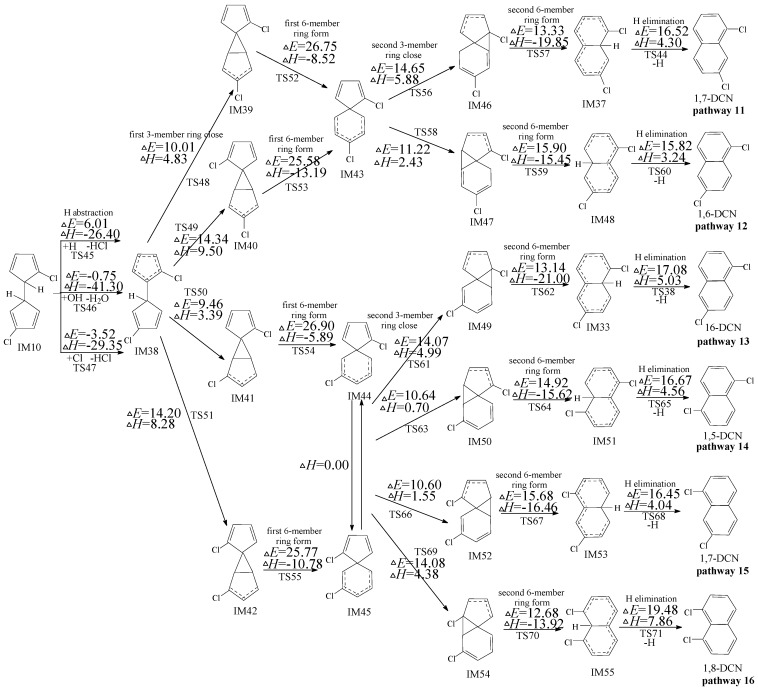
PCN formation routes embedded with the potential barriers Δ*E* (in kcal/mol) and reaction heats Δ*H* (in kcal/mol) from IM10 initiated H abstraction with ortho-carbon Cl substitute. Δ*H* is calculated at 0 K.

**Figure 4:**
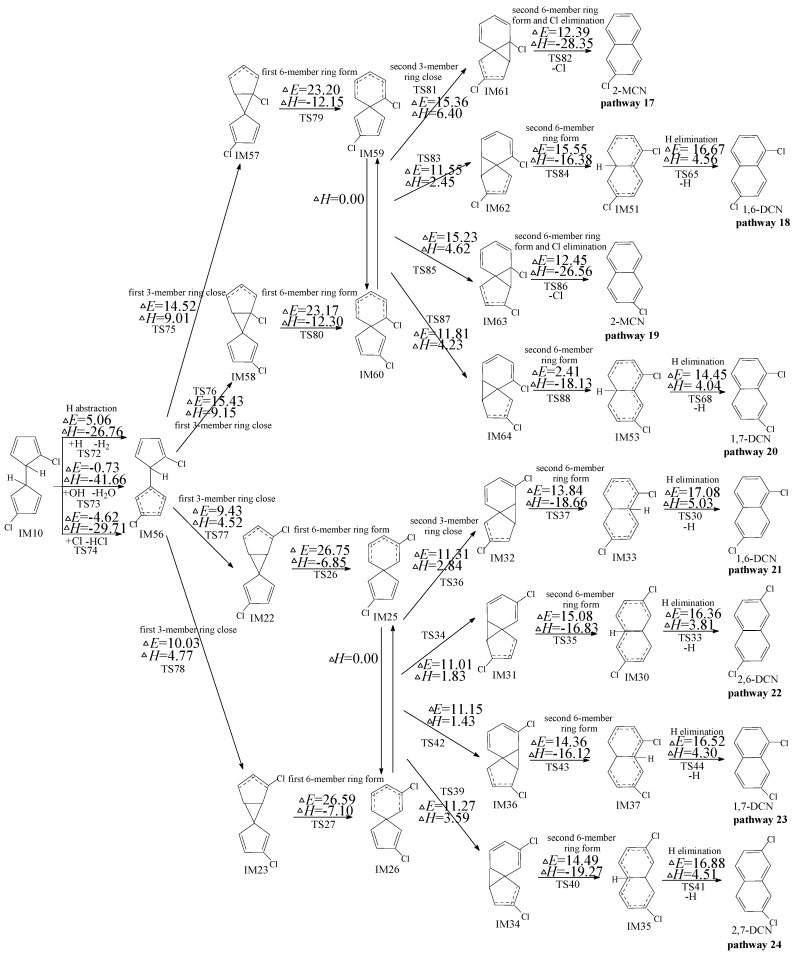
PCN formation routes embedded with the potential barriers Δ*E* (in kcal/mol) and reaction heats Δ*H* (in kcal/mol) from IM10 initiated H abstraction with *meta*-carbon Cl substitute. Δ*H* is calculated at 0 K.

**Figure 5:**
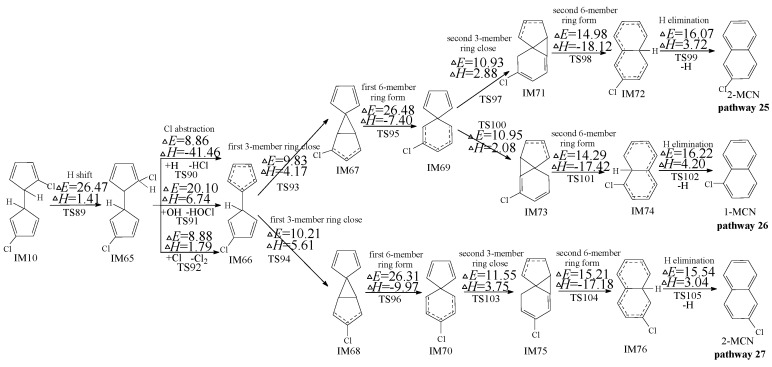
PCN formation routes from IM10 proposed by Kim [32,33], starting with H-shift step. These routes are embedded with the potential barriers Δ*E* (in kcal/mol) and reaction heats Δ*H* (in kcal/mol). Δ*H* is calculated at 0 K.

**Figure 6:**
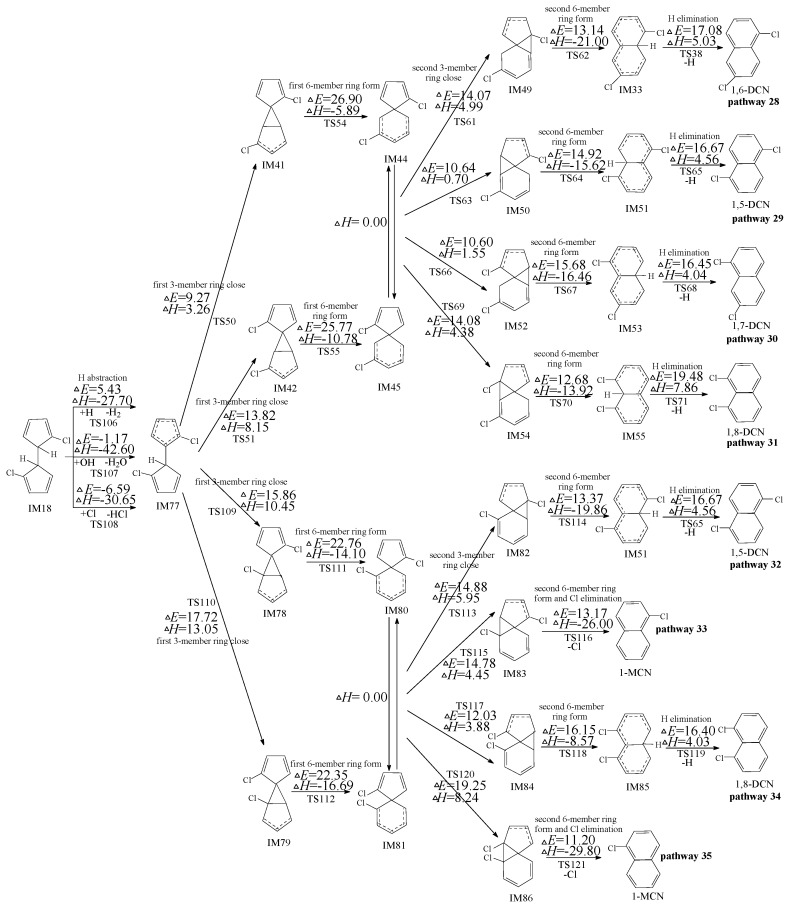
PCN formation routes from IM18 embedded with the potential barriers Δ*E* (in kcal/mol) and reaction heats Δ*H* (in kcal/mol). Δ*H* is calculated at 0 K.

Formation of PCNs from dimerization of 3-CPRs contains two progressions: formation of chloro-dihydrofulvlene (IM5, IM10 and IM18) from dimerization of 3-CPRs (Figure 1) and formation of PCNs from subsequent reactions of chloro-dihydrofulvene (Figure 2, Figure 3, Figure 4, Figure 5 and Figure 6). The configurations of the transition states involved in one typical route of PCN formation are depicted in Figure S1 of the Suporting Information. Imaginary frequencies, zero point energies, and total energies for all the transition states involved in the formation of PCNs from dimerization of 3-CPRs are shown in Table S1 of the Suporting Information. Cartesian coordinates for the reactants, intermediates, transition states, and products involved in this paper are revealed in Tables S2 and S3.

Figure 1 demonstrates the first progress of PCN formation from dimerization of 3-CPRs, e.g., formation of chloro-dihydrofulvene. As shown in Figure 1, three chloro-dihydrofulvenes (IM5, IM10, and IM18) from four possible formation pathways (pathways 1−4) are proposed from the dimerization of 3-CPRs. The potential barriers (Δ*E*) and reaction heats (Δ*H*) are calculated at the MPWB1K/6-311+G(3df,2p)//MPWB1K/6-31+G(d,p) level. All the four pathways are similar, involving five elementary steps: (1) carbon-carbon coupling; (2) first phenolic ring open; (3) first CO elimination; (4) second phenolic ring open; and (5) second CO elimination. There are three carbon-carbon coupling modes in Figure 1, resulting in the formation of intermediates IM1, IM6, and IM14. Different from dimerization of 2-CPRs, all the three carbon-carbon coupling modes are the coupling of two carbon(hydrogen)-centered radical mesomers (CH/CH for short), because all the two *ortho*-positions in 3-CPR are H atoms. All the three CH/CH coupling are barrierless and strongly exothermic. The first/second CO elimination step is a concerted reaction with a five-member ring form simultaneously. Comparing the four chloro-dihydrofulvene formation pathways, the barrier of each step in pathway 1 is lower than 50 kcal/mol, whereas one step has a potential barrier about 50 kcal/mol in pathway 2 and pathway 3, and one step has barrier higher than 52 kcal/mol in pathway 4. Thus, pathway 1 is the most feasible energetically, and pathway 4 is the most difficult to occur. However, at high temperature conditions in combustion and thermal processes, these potential barriers are easily overcome. Thus, all pathways 1–4 are energetically feasible.

### 2.2. Formation of PCNs from Subsequent Reactions of IM5

Four PCNs products (1,6-/1,7-DCNs and 2,6-/2,7-DCNs) from six possible reaction pathways (pathways 5−10) are proposed for the subsequent reactions of IM5 in Figure 2. In Figure 2, pathways 5−10 are similar, involving the following six elementary steps: (1) H abstraction; (2) first three-member ring close; (3) first six-member ring form; (4) second three-member ring close; (5) second six-member ring form; and (6) H elimination. The H atom can be abstracted by H, OH, and Cl radicals with low potential barriers and quite negative reaction heats. H abstraction of IM5 by OH and Cl radicals exhibit negative barriers due to the existence of prereactive complexes. The first six-member ring form step requires crossing a high barrier and is the rate-determining step for pathways 5−10. IM25 and IM26 are enantiomers that produce the same subsequent intermediate (IM31, IM32, IM34, and IM36). From Figure 2, pathway 5 and pathway 6 have the same rate-determining step with the potential barriers 26.38 and 26.43 kcal/mol, whearas pathways 7−10 have the same rate-determining step with the potential barriers 26.75 and 26.59 kcal/mol. The potential barriers of the rate-determining steps for all the six pathways are close, *i.e.*, pathways 5−10 are competitive. Thus all the PCN formation pathways in Figure 2 are feasible, resulting in the formation of 1,6-/1,7-DCNs and 2,6-/2,7-DCNs. Among the four DCN formation pathways, 1,6-/1,7-DCN are obtained only via two pathways (pathways 8 and 10), whereas 2,6-/2,7-DCN are obtained via four pathways (pathways 5, 6, 7, and 9). Thus, the formation potential of 2,6-/2,7-DCNs is larger than that of 1,6-/1,7-DCNs. The main PCN products from subsequent reactions of IM5 are 2,6-/2,7-DCNs.

### 2.3. Formation of PCNs from Subsequent Reactions of IM10

Owning to the asymmetry of IM10, the subsequent reactions of IM10 can occur initiated by two H abstraction modes: H abstraction with *ortho*-carbon Cl substitute (in Figure 3) and H abstraction with *meta*-carbon Cl substitute (in Figure 4). The potential barriers (Δ*E*) and reaction heats (Δ*H*) are calculated at the MPWB1K/6-311+G(3df,2p)//MPWB1K/6-31+G(d,p) level. In Figures 3, four PCN products (1,5-/1,6-/1,7-/1,8-DCNs) from six possible reaction pathways (pathways 11–16) are proposed for the subsequent reactions of IM10 initiated by H abstraction with *ortho*-carbon Cl substitute. All the six pathways start with H abstraction step by H, OH, and Cl radicals, followed by two three-member ring close steps (first/second 3-member ring close) and two 6-member ring form steps (first/second six-member ring form) and ended by the H elimination step. The rate-determining step for pathways 11–16 is also the first six-member ring form. Pathway 11 and pathway 12 have the same rate-determining step. (potential barrier 26.75 and 25.58 kcal/mol). IM44 and IM45 are enantiomers that produce the same subsequent intermediate (IM49, IM50, IM52 and IM54). Pathways 13−16 have the same rate-determining step (potential barrier 26.90 and 25.77 kcal/mol), and the differences of pathways 13−16 occur in the last three elementary steps. The H elimination step in pathway 16 involves the highest barrier (19.48 kcal/mol) and is most endoergic (7.86 kcal/mol) than any elementary step in the last three elementary steps of pathways 13–16, e.g., pathways 13−15 (resulting in 1,5-/1,6-/1,7-DCNs) are thermodynamically favored compared with pathway 16 (resulting in 1,8-DCN). Thus, 1,8-DCN is not the main product of subsequent reactions of IM10 in Figure 3. This agrees well with the experiment result that only trace amounts of 1,8-DCN were observed from 3-CP as precursor [32,33]. Comparing pathway 13−15 with pathways 11−12, all the five pathways occur via the same elementary steps, and potential barrier of rate-determining steps are closer. Thus pathways 11−15 are energetically competitive, resulting in the formation of 1,5-/1,6-/1,7-DCNs. The main products of the subsequent reactions of IM10 initiated H abstraction with *ortho*-carbon Cl substitute are 1,5-/1,6-/1,7-DCNs.

Figure 4 shows the subsequent reactions of IM10 starting at H abstraction with *meta*-carbon Cl substitute. In Figure 4, pathways 18, 20, 21, 22, 23, and 24 are homologous, which are ended with the H elimination step and contain the same six elementary steps as those mentioned above. The rate-determining step for pathways 18, 20, 21, 22, 23, and 24 is the first six-member ring form step. Pathways 17 and 19 are similar. The second 6-member ring form and Cl elimination in pathways 17 and 19 is a concerted reaction occurring in a one-step. Thus pathways 17 and 19 involve five elementary steps: (1) H abstraction; (2) first three-member ring close; (3) first six-member ring form; (4) second three-member ring close; and (5) second six-member ring form and Cl elimination. The rate-determining step for pathways 17 and 19 is also the first 6-member ring form step. From Figure 4, pathways 17−20 occur via a pair of enantioners, denoted as IM59 and IM60, and the same rate-determining step (potential barrier 23.20 and 23.17 kcal/mol); pathways 21−24 occur via a pair of enantioners (IM25 and IM26) and the identical rate-determining step (potential barrier 26.75 and 26.59 kcal/mol). The rate-determining steps for pathways 17−20 require relative lower potential barrier than those of pathways 21−24. In addition, pathways 17 and 19 have one step less than pathways 18, 20, 21, 22, 23, and 24. Thus, pathways 17 and 19 are energetically preferred than pathways 18, 20, 21, 22, 23, and 24, *i.e.*, pathways ended with Cl elimination are favored over those ended with H elimination, resulting in the formation of 2-MCN. The main product of the subsequent reactions of IM10 initiated H abstraction with *meta*-carbon Cl substitute is 2-MCN.

In Kim’s mechanism, the chloro-dihydrofulvene occurs intramolecular rearrangement of 1,5-sigmatropic shifts of H/Cl to the *ortho*-carbon of cyclopentadiene ring before *ortho*-carbon H/Cl atom is abstracted by H, OH and Cl radicals. However, this deduction has been refused in our previous study on PCN formation from 2-CP and 4-CP [47,48], owning to the high potential barrier of 1,5-sigmatropic shift step. Similarly, theoretical calculation of 1,5-sigmatropic H shift mechanism of IM10 is shown in Figure 5. Compared with the H direct abstraction routes in Figure 3, the H shift mode requires crossing a high barrier (26.47 kcal/mol) and is endothermic (1.41 kcal/mol). This reconfirms the conclusion that the direct H abstraction mechanism shown in Figure 3 is energetically favored over H shift mechanisms shown in Figure 5.

### 2.4. Formation of PCNs from Subsequent Reactions of IM18

Figures 6 shows formation of PCNs from subsequent reactions of IM18 embdded with the potential barriers (Δ*E*) and reaction heats (Δ*H*) at the MPWB1K/6-311+G(3df,2p)//MPWB1K/6-31+G(d,p) level. Eight possible reaction pathways (pathways 28–35) are proposed for the subsequent reactions of IM10 to form five PCNs (1-MCN and 1,5-/1,6-/1,7-/1,8-DCNs). From Figure 6, pathways 28, 29, 30, 31, 32, and 34 are alike, and they involve six elementary steps similar as the six-step pathways mentioned above (ended with H elimination step). Pathways 33 and 35 are homologous, and they contain five elementary steps similar as five-step pathways mentioned above (ended with a concerted second 6-member ring form and Cl elimination step). The rate-determine step for pathways 28−35 is the first 6-member ring form step. From Figure 6, pathways 28−31 have the same rate-determining step (potential barriers 26.90 and 25.77 kcal/mol), and pathways 32−35 have the identical rate-determining steps (potential barriers 22.76 and 22.35 kcal/mol). The rate-determining step of pathway 32−35 require crossing lower potential barriers than those of pathway 28−31. In addition, pathway 33 and 35 have one step less than pathways 28, 29, 30, 31, 32, and 34. Thus, pathway 33 and 35 are favored over pathways 28, 29, 30, 31, 32, and 34, resulting in the formation of naphthalene (1-MCN). This re-verifies the conclusions that pathways ended with Cl elimination are energetically preferred than those ended with H elimination. 1-MCN is the main PCN products of subsequent reactions of IM18.

### 2.5. Comparison of PCN Formation Mechanism with the Experimental Observation

**Figure 7:**
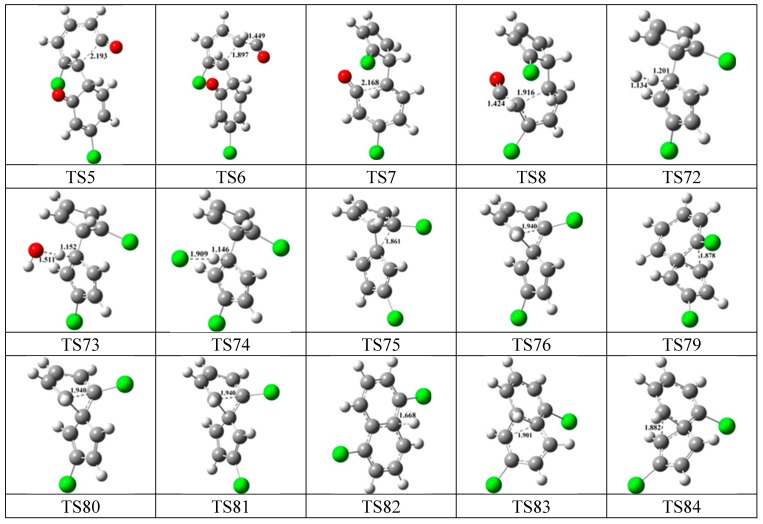
Configurations of the transition states involved in one typical route of PCN formation. Distances are in angstroms. Gray sphere, C; White sphere, H; Red sphere, O; Green sphere, Cl. (For interpretation of the references to color in this figure legend, the reader is referred to the web version of this article.)

To sum up Figure 1, Figure 2, Figure 3, Figure 4, Figure 5 and Figure 6, the main PCN products from subsequent reactions of IM5 are 2,6-/2,7-DCNs in Figure 2; the main PCN products from subsequent reactions of IM10 are 1,5-/1,6-/1,7-/DCNs in Figure 3 and 2-MCN in Figure 4; the main PCN products from subsequent reactions of IM18 are 1-MCN in Figure 6. This can give a reasonbale explanation for the experimental observations that 3-CP produced nearly equal amounts of 1- and 2-MCN and nearly equal amounts of 1,5-/1,6-/1,7-DCNs and 2,6-/2,7-DCNs [32,33]. From both Kim’s and Yang’s experiments, the formation potential of MCN products are larger than that of DCN [27,32,33], which can be explained from three aspects. First, MCNs are formed from five elementary steps, with one step less than the DCNs formation. Secondly, the rate-determined steps of MCN formation pathways require crossing lower potential barriers than that of DCN formation pathways. Thirdly, more MCN products may come from the cross-reaction of phenoxy radicals with 3-CPR because CPs are easy to lose Cl atoms at high temperatures to form phenol, which was also detected in the Kim’s experiments [32,33]. The formation of phenol can be reconfirmed by the large amount of N formation from 3-CP as a precursor in both Kim and Yang’s experiments, which are not existent in our mechanism from dimerization of 3-CPRs. Presumably, N is formed from the cross-reaction of phenoxy radicals with 3-CPR with one Cl atom loss or the self-reaction of phenoxy radicals. Configurations of the transition states involved in one typical route of PCN formation of Figure 1, Figure 2, Figure 3, Figure 4, Figure 5 and Figure 6 are depicted in Figure 7.

### 2.6. Comparison of PCN Formation from 2-CP, 3-CP, and 4-CP

Comparison of the formation of PCNs from the 3-CP with our previous studies of PCN formation from 2-CP and 4-CP precursors [47,48] shows that the position of chlorination affects each elementary step involved in the PCN formation. On the one hand, the isomer patterns of PCNs products are completely different from the three CPs. To sum up the relationship of PCN isomers with chlorinated dihydrofulvalene, it could be concluded that the *ortho*-Cl in dihydrofulvene would finally mainly located in the *alpha* position of naphthalene or be elimitated, but rarely located in *beta* position of naphthalene; the *meta*-Cl in dihydrofulvene would be finally mainly located in the *beta* position of naphthalene (could not be eliminated), rarely located in the *alpha* position of naphthalene. For 2-CP, it mainly forms dihydrofulvene with two *ortho*-Cl atoms. Thus, the finally main DCN production from 2-CP are DCNs with Cl atoms mainly located in the *alpha* position of naphthalene (1,5-/1,6-/1,7-DCNs), and the finally main MCN products from 2-CP is MCN eliminated one Cl and with other Cl located in the *alpha* position (1-MCN). For 4-CP, it mainly produces dihydrofulvene with two *meta*-Cl atoms. Thus the finally main DCN products from 4-CP are DCNs with Cl atoms maily located in the *beta* position of naphthalene and rarely located in the *alpha* position (2,6-/2,7-DCNs). For 3-CP, it can form three dihydrofulvenes: one is the dihydrofulvene with two *ortho*-Cl, one is the dihydrofulvene with two *meta*-Cl, and one is with one *ortho* and one *meta*-Cl atoms. Thus, 3-CP can, almost equally, produce PCNs with Cl atoms located in the *alpha* position (1,5-/1,6-/1,7-DCNs, 1-MCN) and PCNs with Cl atoms located in the *beta* position (2,6-/2,7-DCNs, 2-MCN). Hence, for MCNs, 2-CP mostly produced 1-MCN, 3-CP produced approximately equal amounts of 1-MCN and 2-MCN, and 4-CP can not produce MCN. For DCNs, 2-CP mostly produce 1,5-/1,6-/1,7-DCNs, 3-CP produced approximately equal amounts of 1,5-/1,6-/1,7-DCNs and 2,6-/2,7-DCNs, and 4-CP almost exclusively produced 2,6-/2,7-DCNs. These results agree well with Kim’s observations [32,33] and oppose the previous hypothesis of PCN formation mechanism via CO loss of CPRs to form the same chloro-CPDyl radical, which would produce the same PCNs products [26,27,28,29].

On the other hand, the position of chlorination also affects PCN formation potential. In Kim’s experiment, 2-CP produced the lowest amount of PCNs [32,33]. This result could be explained by the higher potential barrier and less exothermic property of the phenolic-hydrogen abstraction of 2-CP than that of 3-CP and 4-CP, as mentioned in our previous studies [49,50], owning to intramolecular hydrogen bonding (enhancing the O-H bond in 2-CP) and the inductive effect of the electron-withdrawing chlorine (weakening the O-H bond in 4-CP) [49,50]. For example, the potential barrier of phenoxyl-hydrogen abstraction in 2-CP, 3-CP, and 4-CP by OH radical is 3.20, 0.17 and −0.83 kcal/mol, respectively, and the reaction heat of phenoxyl-hydrogen abstraction in 2-CP, 3-CP, and 4-CP by OH radical is −12.01, −12.94 and −15.13 kcal/mol [49,50]. By In addition, dimerization of CPRs is barrierless and is the initial step of PCN formation. The dimerization of 3-CPRs (−23.42, −23.25 and −23.60 kcal/mol) is approximately 10 kcal/mol more exothermic than those of 2-CPRs (−17.63 and −10.29 kcal/mol) and 4-CPRs (−16.20 kcal/mol) [47,48]. Thus, 3-CP have stronger PCN formation potential than that of 2-CP and 4-CP [46,47], which is consistent with the experimental result wherein 3-CP produced the highest PCN yield [32,33].

### 2.7. Rate Constant Calculations

In the environmental field, mathematical models have been extensively applied in pollutants policy formulation, quantitative prediction, and risk analyses by predicting the potential sources and emissions of contaminant releases to the environment. For example, the PCN formation models and PCN controlling kinetic models can account for the potential outcomes of PCNs to the environment and the gaseous route in the production of PCNs in combustion and thermal processes. Among different parameters, the rate constants can be used as important input parameters for constructing and improving PCN mathematical models. However, owing to the limitation of experimental conditions and lack of the effective detection methods, it is difficult to measure, experimentally, the rate constants of the elementary reactions, especially the reactions involving the short-life radical intermediates in the formation of PCNs. In such a situation, an alternative method is to use the calculated rate constant or other dynamical information directly from quantum calculations of electronic structure, frequency, and energy.

In this paper, the rate constants of the crucial elementary reactions for the formation of PCNs from 3-CP were calculated by using canonical variational transition state theory (CVT) with small-curvature tunneling (SCT) contribution methods [51,52,53,54]. The CVT/SCT method is among the most promising current avenues of approach in theoretical chemical kinetics. The error correction of the kinetic calculation may be mainly from the SCT method [51,52,53,54]. The reliability and accuracy of the CVT/SCT method was verified in our recent studies [47,48,49,50,55,56,57,58]. For example, the CVT/SCT rate constants of C_6_H_5_OH + H → C_6_H_5_O + H_2_ and C_6_H_5_OH + OH → C_6_H_5_OH + H_2_O are in good agreement with the corresponding experimental values [49,50], respectively. In addition, the CVT/SCT rate constants of 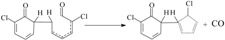 are matches well with the available literature rate constant values [59,60] for structurally-similar compounds 
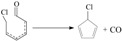
. Actually, the CVT/SCT method has been successfully performed for the the rate calculations in our previous studies involved in the formation of PCDD/Fs from various CP precursors [55,56,57,58] and PCN formation from 2-CP and 4-CP precursors [47,48], and are clarified to be an efficient method to calculate the rate constants.

To be used more effectively, the CVT/SCT rate constants every 50 K from 600 to 1200 K were calculated, for elementary reactions involved in the thermodynamically preferred formation pathways of PCNs from 3-CP as precursor. The 600−1200 K covers the possible formation temperature range of PCN in municipal waste incinerators. The calculated CVT/SCT rate constants under different temperatures are fitted in the Arrhenius form, as shown in Table 1. The pre-exponential factors, the activation energies, and the rate constants can be obtained from these Arrhenius.

**Table 1:** Arrhenius formulas for crucial elementary reactions involved in the formation of PCNs from the 3-CP precursor over the temperature range of 600−1200 K (units are s^−1^ and cm^3^·molecule^−1^·s^−1^ for unimolecular and bimolecular reactions, respectively).

Reactions	Arrhenius Formulas
3-TCP + H → 3-MCPR + H_2_	*k*(T) = (2.36 × 10^−^^12^) exp (−6579.12/T)
3-TCP + OH → 3-MCPR + H_2_O	*k*(T) = (3.12 × 10^−^^12^) exp (−1512.16/T)
3-TCP + O(^3^P) → 3-MCPR + OH	*k*(T) = (3.50 × 10^−^^11^) exp (−4659.85/T)
IM1 → IM2 via TS1	*k*(T) = (4.06 × 10^13^) exp (−20,030.86/T)
IM2 → IM3 + CO via TS2	*k*(T) = (9.43 × 10^9^) exp (−24,696.87/T)
IM3 → IM4 via TS3	*k*(T) = (5.13 × 10^13^) exp (−20,235.56/T)
IM4 → IM5 + CO via TS4	*k*(T) = (2.51 × 10^11^) exp (−23,661.43/T)
IM6 → IM7 via TS5	*k*(T) = (3.64 × 10^13^) exp (−20,292.91/T)
IM7 → IM8 + CO TS6	*k*(T) = (1.68 × 10^11^) exp (−25,390.77/T)
IM8 → IM9 via TS7	*k*(T) = (2.35 × 10^13^) exp (−36,819.88/T)
IM9 → IM10 + CO via TS8	*k*(T) = (4.10 × 10^12^) exp (−22,081.08/T)
IM6 → IM11 via TS9	*k*(T) = (1.10 × 10^14^) exp (−19,945.07/T)
IM11 → IM12 + CO via TS10	*k*(T) = (4.43 × 10^11^) exp (−23,531.77/T)
IM12 → IM13 via TS11	*k*(T) = (1.14 × 10^5^) exp (−18,756.53/T)
IM13 → IM10 + CO via TS12	*k*(T) = (5.95 × 10^9^) exp (−22,995.33/T)
IM14 → IM15 via TS13	*k*(T) = (5.70 × 10^13^) exp (−19,945.04/T)
IM15 → IM16 + CO via TS14	*k*(T) = (7.21 × 10^1^°) exp (−26,688.66/T)
IM16 → IM17 via TS15	*k*(T) = (1.53 × 10^13^) exp (−17,862.44/T)
IM17 → IM18 + CO via TS16	*k*(T) = (3.25 × 10^11^) exp (−23,917.06/T)
IM10 + H → IM56 + H_2_ via TS72	*k*(T) = (6.92 × 10^−^^11^) exp (−2926.10/T)
IM56 → IM57 via TS75	*k*(T) = (1.37 × 10^12^) exp (−7741.24/T)
IM56 → IM58 via TS76	*k*(T) = (1.08 × 10^12^) exp (−8010.75/T)
IM57 → IM59 via TS79	*k*(T) = (1.67 × 10^13^) exp (−11,934.50/T)
IM58 → IM60 via TS80	*k*(T) = (6.96 × 10^13^) exp (−11,934.96/T)
IM59/IM60 → IM61 via TS81	*k*(T) = (2.24 × 10^11^) exp (−9660.05/T)
IM61 → 2-MCN + Cl via TS82	*k*(T) = (2.21 × 10^13^) exp (−6651.99/T)
IM59/IM60 → IM63 via TS85	*k*(T) = (3.02 × 10^12^) exp (−7870.98/T)
IM63 → 2-MCN + Cl via TS86	*k*(T) = (1.89 × 10^13^) exp (−6658.32/T)
IM18 + H→IM77 + H_2_ via TS106	*k(T)=* (6.77 × 10^−^^13^) exp (−3847.80/T)
IM77 → IM78 via TS109	*k*(T) = (3.10 × 10^12^) exp (−8185.85/T)
IM77→ IM79 via TS110	*k*(T) = (4.05 × 10^12^) exp (−9142.08/T)
IM78 → IM80 via TS111	*k*(T) = (1.70 × 10^13^) exp (−11,846.10/T)
IM79 → IM81 via TS112	*k*(T) = (1.82 × 10^13^) exp (−11,683.31/T)
IM80/IM81 → IM83 via TS115	*k*(T) = (2.73 × 10^12^) exp (−7528.18/T)
IM83 → 1-MCN + Cl via TS116	*k*(T) = (1.98 × 10^13^) exp (−6998.83/T)
IM80/IM81 → IM86 via TS120	*k*(T) = (7.40 × 10^11^) exp (−8061.95/T)
IM86 → 1-MCN + Cl via TS121	*k*(T) = (3.52 × 10^13^) exp (−6073.38/T)

## 3. Experimental Section

### 3.1. Density Functional Theory

All the calculations on the geometries, energies, frequencies for reactants, complexes, transition states, and products were determined using the Gaussian 09 program package [61]. The hybrid meta function MPWB1K was employed for the homogeneous gas-phase formation of PCNs from 3-CP precursor, which has uniformly good performance in quantum calculations of thermochemistry, thermochemical kinetics, hydrogen bonding, and weak interactions [62]. The reliability and accuracy of the MPWB1K method for the geometries, vibrational frequencies, and energy calculation of PCNs formation from CPs have been confirmed in our previous works on PCNs formation from 2-CP and 4-CP as precursors [47,48]. The geometries, vibrational frequencies, and the intrinsic reaction coordinate (IRC) calculations were carried out at the MPWB1K/6-31+G(d,p) level. The vibrational frequency calculations were used to determine the nature of minima and first-order saddle points and to provide the zero-point energy (ZPE) and the thermal contributions to the free energy of activation. The minimum energy path (MEP) was obtained by the IRC calculation to confirm that the transition state really connects to minima along the reaction path [63]. To obtain more reliable potential barriers and reaction heats, a more flexible basis set, 6-311+G(3df,2p), was used to determine the single-point energies of the various species, based on the optimized geometries. All the relative energies quoted and discussed in this paper include ZPE corrections.

### 3.2. Kinetic Calculation

The canonical variational transition state theory (CVT) with small-curvature tunneling (SCT) correction is an effective method to calculate the rate constants [50,51,52,53,54]. In this paper, the CVT/SCT method is used to calculate the rate constants of key elementary step involved in this study over a wide temperature range (600−1200 K) by using the Polyrate 9.7 program [64]. The level of tunneling calculation is the small curvature tunneling (SCT) [54] method, based on the centrifugal-dominant small-curvature semi-classical adiabatic ground-state approximation. The rotational partition functions were calculated classically, and the vibrational modes were treated as quantum-mechanically separable harmonic oscillators. The error of the kinetic calculation may be mainly from the small curvature tunneling (SCT) method, especially for the heavy-light-heavy (HLH) mass combination reactions. The large-curvature tunneling approximation may be especially desirable to model these kind of reactions in detail. Methods for large curvature cases have been developed [65], but they require more information about the potential energy surface than that was determined in the present study. Thus, the SCT approximation becomes to be an alternative to the large-curvature approximation. The new CD-SCSAG approximation for small-curvature tunneling is an improvement over the original SCSAG small-curvature method in that it accounts for the effect of mode-mode coupling on the extent of corner cutting through each vibrational degree of freedom orthogonal to the reaction path [66]. To calculate the rate constants, 40 non-stationary points near the transition state along the minimum energy path (20 points on the reactants side and 20 points on the product side) were selected for frequency calculations at the MPWB1K/6-31 + G(d,p) level. The parameters such as energy data, matrices of force constants, Hessian matrixes, coordinates of each stationary points, and unstationary points are obtained from the Gaussian 09 program output files and are input into the polyrate input files automatically by our self-compile program.

## 4. Conclusions

In this study, the mechanism of the homogeneous gas-phase formation of PCNs from the 3-CP precursor were investigated theoretically using DFT electronic structure theory at the MPWB1K/6-311+G(3df,2p)//MPWB1K/6-31+G(d,p) level. The kinetic calculation was performed and the rate constants were calculated over the temeperature range of 600−1200 K using canonical variational transition-state (CVT) theory with the small curvature tunneling (SCT) contribution. The rate temperature formulas were fitted. The mechanism in this paper can well explain the experimental observation. The obtained rate constant can support more detailed input parameters for the PCN controlling and prediction models. This study is an ongoing work of our previous studies of PCN formation from 2-CP and 4-CP, so the effect of the position of chlorination on PCN formation were discussed. Four specific conclusions can be drawn:

(1) Dimerization of chlorophenoxy radicals (CPRs) contains three CH/CH coupling modes, resulting in three chloro-dihydrofulvene. The formation pathways of the three chloro-dihydrofulvene are all energetically feasible.

(2) The subsequent reactions of chloro-dihydrofulvene contains two different pathwways. The pathways ended with Cl elimination are favored over those ended with H elimination.

(3) Both 1-MCN and 2-MCN are the main MCN products from 3-CP precursor, and both 1,5-/1,6-/1,7-DCNs and 2,6-/2,7-DCNs are the main DCN products from 3-CP precursor. The formation potential of MCNs with one chlorine loss is larger than that of DCNs without chlorine loss.

(4) The position of chlorination in monochlorophenol affect both isomer patterns and formation potential of PCNs products.

## Supplementary Materials

The imaginary frequencies, the zero-point energies and the total energies for the transition states involved in the formation of PCNs from the 3-CP precursor. Cartesian coordinates for the reactants, intermediates, transition states and products involved in PCN formation from 3-CP. Supplementary materials can be found at http://www.mdpi.com/1422-0067/16/09/20620/s1.
