# Supplementary material for: Quantum Chemical and Kinetic Study on Polychlorinated Naphthalene Formation from 3-Chlorophenol Precursor
Source: Int J Mol Sci. 2015 Aug 31;16(9):20620–40. doi: 10.3390/ijms160920620 (PMC4613222; doi:10.3390/ijms160920620)
Supplement: Supplementary file 1 [file ijms-16-20620-s001.pdf]

Supplementary Information

Table S1. Imaginary frequencies (in cm^{-1}), zero point energies (ZPE, in a.u.) and total energies (in a.u.) for the transition states involved in the formation of PCNs from the 3-CP as precursor.

Transition States	Imaginary Frequencies	Total Energies	Transition States
TS1	515i	0.17046	−1532.88062
TS2	738i	0.16992	−1532.81866
TS3	526i	0.15965	−1419.55076
TS4	725i	0.15878	−1419.48753
TS5	443i	0.17091	−1532.88314
TS6	686i	0.16960	−1532.81192
TS7	567i	0.15937	−1419.54692
TS8	707i	0.15877	−1419.49045
TS9	525i	0.17046	−1532.88010
TS10	725i	0.16967	−1532.81725
TS11	473i	0.16001	−1419.55252
TS12	681i	0.15849	−1419.48159
TS13	439i	0.17102	−1532.88203
TS14	662i	0.16927	−1532.80615
TS15	471i	0.16028	−1419.55305
TS16	650i	0.15924	−1419.48704
TS17	1270i	0.15014	−1306.77309
TS18	302.7i	0.16034	−1381.98726
TS19	111i	0.15042	−1766.49827
TS20	506i	0.13825	−1305.63904
TS21	506i	0.13834	−1305.63778
TS22	501i	0.13864	−1305.64025
TS23	502i	0.13850	−1305.63891
TS24	735i	0.13766	−1305.60245
TS25	735i	0.13773	−1305.60233
TS26	686i	0.13792	−1305.60472
TS27	683i	0.13816	−1305.60482
TS28	633i	0.13856	−1305.64204
TS29	743i	0.13892	−1305.63208
TS30	1196i	0.13374	−1305.65163
TS31	625i	0.13836	−1305.64164
TS32	788.5i	0.13856	−1305.63000
TS33	1190i	0.13381	−1305.65198
TS34	614i	0.13846	−1305.64127
TS35	746i	0.13905	−1305.63245
TS36	615i	0.13831	−1305.64064
TS37	776i	0.13883	−1305.63259
TS38	1166i	0.13393	−1305.65227
TS39	614i	0.13830	−1305.64069
TS40	791i	0.13871	−1305.63025

Table S1. *Cont.*

Transition States	Imaginary Frequencies	Total Energies	Transition States
TS41	1178i	0.13379	−1305.65223
TS42	61i	0.13851	−1305.64109
TS43	729i	0.13910	−1305.63475
TS44	117i	0.13400	−1305.65189
TS45	1333i	0.15002	−1306.77047
TS46	53i	0.15984	−1381.98008
TS47	59i	0.15057	−1766.49694
TS48	472i	0.13831	−1305.63841
TS49	494i	0.13851	−1305.63171
TS50	461i	0.13848	−1305.63947
TS51	479i	0.13860	−1305.63202
TS52	734i	0.13785	−1305.60359
TS53	756i	0.13787	−1305.59803
TS54	680i	0.13791	−1305.60570
TS55	714i	0.13799	−1305.59979
TS56	688i	0.13839	−1305.63698
TS57	705i	0.13871	−1305.63004
TS58	621i	0.13868	−1305.64275
TS59	790i	0.13885	−1305.63158
TS60	1192i	0.13376	−1305.65124
TS61	677i	0.13825	−1305.63588
TS62	724i	0.13881	−1305.62995
TS63	607i	0.13860	−1305.64168
TS64	778i	0.13908	−1305.63423
TS65	1172i	0.13402	−1305.65128
TS66	605i	0.13851	−1305.64166
TS67	796i	0.13897	−1305.63155
TS68	1183i	0.13387	−1305.65146
TS69	676i	0.13838	−1305.63599
TS70	708i	0.13895	−1305.63181
TS71	1150i	0.13379	−1305.63799
TS72	1242i	0.15014	−1306.77213
TS73	289i	0.16046	−1381.98790
TS74	128i	0.14982	−1766.49794
TS75	568i	0.13851	−1305.63200
TS76	566i	0.13835	−1305.63039
TS77	501i	0.13864	−1305.64025
TS78	498i	0.13859	−1305.63923
TS79	746i	0.13810	−1305.60341
TS80	740i	0.13835	−1305.60348
TS81	655i	0.13839	−1305.63556
TS82	734i	0.13851	−1305.63020
TS83	631i	0.13872	−1305.64195
TS84	748i	0.13905	−1305.63199

Table S1. *Cont.*

Transition States	Imaginary Frequencies	Total Energies	Transition States
TS85	653i	0.13859	−1305.63595
TS86	661i	0.13888	−1305.63331
TS87	625i	0.13854	−1305.64136
TS88	400i	0.13908	−1305.65015
TS89	1239i	0.14895	−1306.23681
TS90	1036i	0.15341	−1306.76708
TS91	657i	0.16263	−1381.97759
TS92	490i	0.15089	−1766.47524
TS93	489i	0.14801	−845.954722
TS94	492i	0.14770	−845.953806
TS95	686i	0.14748	−845.921019
TS96	733i	0.14725	−845.918764
TS97	602i	0.14791	−845.958022
TS98	797i	0.14827	−845.947346
TS99	1181i	0.14377	−845.969967
TS100	604i	0.14798	−845.958055
TS101	780i	0.14850	−845.949945
TS102	1166i	0.14366	−845.969792
TS103	618i	0.14802	−845.958947
TS104	792i	0.14808	−845.947188
TS105	1190i	0.14372	−845.969695
TS106	1323i	0.15024	−1306.76936
TS107	528i	0.16039	−1382.00922
TS108	448i	0.14837	−1766.49737
TS109	543i	0.13830	−1305.62888
TS110	477i	0.13791	−1305.62552
TS111	745i	0.13830	−1305.60123
TS112	764i	0.13818	−1305.59762
TS113	684i	0.13859	−1305.63655
TS114	719i	0.13882	−1305.62969
TS115	647i	0.13863	−1305.63674
TS116	721i	0.13885	−1305.63244
TS117	638i	0.13881	−1305.64130
TS118	786i	0.13879	−1305.62853
TS119	1181i	0.13368	−1305.63669
TS120	708i	0.13855	−1305.62953
TS121	651i	0.13843	−1305.62911

Table S2. Cartesian coordinates for the reactants, intermediates and products involved in PCN formation from 3-CP, x coordinat, y coordinat and z coordinat.

IM	x	y	z
1-MCN			
0	1		
C	2.902601	−0.599853	−0.000160
C	2.383950	0.660792	−0.000252
C	0.126998	−0.252906	0.000037
C	0.691279	−1.545116	0.000387
C	2.044842	−1.712717	0.000387
H	3.971450	−0.747066	−0.000574
H	3.036380	1.521404	−0.001120
H	0.037538	−2.401450	0.001129
H	2.459175	−2.709018	0.000511
C	−0.903269	2.361812	0.000227
C	−1.772734	1.259679	0.000376
C	−1.266352	−0.005844	0.000196
C	0.989342	0.872955	−0.000227
C	0.446067	2.173978	−0.000016
H	−1.315976	3.358432	0.000216
H	−2.840466	1.406820	0.000499
H	1.119202	3.018160	−0.000276
Cl	−2.371979	−1.336704	−0.000360
2-MCN			
0	1		
C	−3.310660	0.212849	0.000047
C	−2.331799	1.163178	−0.000045
C	−0.629892	−0.577619	−0.000040
C	−1.662963	−1.538228	−0.000025
C	−2.971272	−1.152385	−0.000075
H	−4.349108	0.505710	0.000260
H	−2.586284	2.212908	0.000054
H	−1.401220	−2.585891	0.000233
H	−3.753540	−1.895559	0.000258
C	1.374571	1.374347	0.000016
C	1.696417	0.006198	0.000037
C	0.729553	−0.952064	−0.000011
C	−0.971112	0.794912	−0.000054
C	0.065339	1.751698	0.000014
H	2.167038	2.104827	0.000029
H	−0.191092	2.800678	−0.000030
H	0.996708	−1.997262	−0.000096
Cl	3.364024	−0.449572	0.000007

Table S2. *Cont.*

IM	x	y	z
1,5-DCN			
0	1		
C	−0.534289	−1.788749	−0.000011
C	−0.599348	−0.381574	−0.000109
C	1.822850	−0.330211	0.000205
C	1.866212	−1.691786	0.000384
C	0.670681	−2.424599	0.000398
H	−1.450584	−2.354394	−0.000164
H	2.818307	−2.196620	0.000281
H	0.713565	−3.502290	0.000784
Cl	3.316964	0.540525	−0.000411
C	−0.670631	2.424608	0.000391
C	−1.866219	1.691784	0.000236
C	−1.822908	0.330260	−0.000264
C	0.599353	0.381545	0.000147
C	0.534299	1.788761	0.000138
H	−0.713524	3.502297	0.000698
H	−2.818294	2.196669	0.000692
H	1.450616	2.354387	0.000257
Cl	−3.316969	−0.540542	−0.000274
1,6-DCN			
0	1		
C	−1.961392	2.255844	0.000097
C	−0.598968	2.251364	0.000007
C	0.111125	1.033444	−0.000004
C	−2.008063	−0.137550	0.000129
C	−2.678525	1.048842	0.000106
H	−2.503735	3.188209	0.000074
H	−0.045801	3.178142	−0.000123
H	−3.756208	1.052844	0.000069
C	1.499123	−1.393664	0.000210
C	2.187924	−0.170288	−0.000098
C	1.521124	1.015645	−0.000148
C	−0.595114	−0.194779	0.000181
C	0.136458	−1.399694	0.000417
H	2.056330	−2.316183	0.000334
H	−0.395867	−2.336662	0.000633
Cl	−2.920297	−1.606513	−0.000257
H	2.063881	1.947698	−0.000342
Cl	3.914370	−0.191666	−0.000098
1,7-DCN			
0	1		
C	−2.838370	1.454389	0.000355
C	−1.659892	2.138336	0.000070
C	−0.432324	1.447700	−0.000122

Table S2. *Cont.*

IM	x	y	z
C	−1.665456	−0.635550	0.000108
C	−2.843444	0.050240	0.000293
H	−3.778276	1.983325	0.000404
H	−1.650021	3.217773	−0.000083
H	−3.774796	−0.492234	0.000316
C	0.816858	−0.648141	0.000387
C	1.978341	0.062598	0.000053
C	1.984773	1.467227	−0.000374
C	0.798840	2.135735	−0.000438
C	−0.417511	0.030613	0.000149
H	0.836331	−1.724747	0.000685
H	0.788963	3.215340	−0.000704
Cl	−1.708981	−2.363503	−0.000355
H	2.924453	1.994808	−0.000556
Cl	3.492655	−0.766682	0.000181
1,8-DCN			
0	1		
C	1.745354	2.406325	0.000495
C	2.382738	1.207211	0.000210
C	1.656287	0.000064	−0.000054
C	−0.391288	1.284686	−0.000004
C	0.347916	2.435088	0.000356
H	2.298632	3.331906	0.000736
H	3.460566	1.152303	0.000177
H	−0.174069	3.377782	0.000436
C	0.348116	−2.435056	−0.000252
C	−0.391186	−1.284716	−0.000034
C	0.225361	0.000015	−0.000058
C	2.382839	−1.207019	−0.000268
C	1.745559	−2.406183	−0.000418
H	−0.173781	−3.377799	−0.000210
H	2.298913	−3.331720	−0.000560
H	3.460662	−1.152012	−0.000279
Cl	−2.102447	1.535312	−0.000632
Cl	−2.102323	−1.535486	0.000623
2,6-DCN			
0	1		
C	−0.753259	−1.698313	0.000491
C	0.215658	−0.673033	−0.000082
C	−1.596095	0.957271	−0.000332
C	−2.497087	−0.063485	−0.000348
C	−2.084097	−1.406538	0.000257
H	−0.426795	−2.727419	0.001125
H	−2.824942	−2.189283	0.000526
C	2.084105	1.406542	0.000422

Table S2. *Cont.*

IM	x	y	z
C	2.497111	0.063475	−0.000046
C	1.596079	−0.957252	−0.000369
C	−0.215654	0.673033	−0.000097
C	0.753294	1.698334	0.000330
H	2.824988	2.189258	0.000652
H	0.426801	2.727429	0.000643
H	−1.933223	1.981786	−0.000190
Cl	−4.190620	0.276446	−0.000070
H	1.933210	−1.981772	−0.000530
Cl	4.190599	−0.276458	−0.000141
2,7-DCN			
0	1		
C	−2.414057	1.206453	0.000027
C	−1.234043	1.887657	−0.000021
C	0.000000	−0.207457	−0.000060
C	−1.228875	−0.898503	−0.000068
C	−2.397058	−0.199101	−0.000047
H	−3.358616	1.725190	0.000058
H	−1.235834	2.967396	0.000078
C	2.414057	1.206453	0.000029
C	2.397058	−0.199102	−0.000048
C	1.228874	−0.898503	−0.000069
C	0.000000	1.206886	−0.000013
C	1.234043	1.887657	−0.000016
H	3.358616	1.725189	0.000062
H	1.235835	2.967396	0.000084
H	1.238439	−1.976909	0.000000
Cl	3.904990	−1.040764	0.000042
H	−1.238440	−1.976909	0.000002
Cl	−3.904990	−1.040765	0.000043
IM1			
0	1		
C	2.226116	−0.708241	−1.210165
C	3.193701	−0.432043	−0.169016
C	2.976376	0.429109	0.835712
C	1.698046	1.112762	0.939542
C	0.727509	0.982307	−0.213284
C	1.065968	−0.055359	−1.219591
H	−0.215825	−0.743648	1.718072
C	−0.987601	−0.374866	1.057746
C	−2.166794	−0.989965	0.995978
C	−0.706982	0.873982	0.304723

Table S2. *Cont.*

IM	x	y	z
C	−3.199377	−0.467482	0.125634
C	−1.706713	1.203151	−0.781136
C	−3.014966	0.572387	−0.700091
H	−2.383356	−1.869558	1.579316
H	0.767366	1.941671	−0.743384
O	1.404864	1.777145	1.911718
H	0.346747	−0.233952	−2.006018
H	2.477929	−1.436756	−1.962994
H	−0.809261	1.686836	1.037285
O	−1.423138	1.980052	−1.668145
Cl	4.677853	−1.293601	−0.264599
Cl	−4.712446	−1.281959	0.170170
H	3.698181	0.596738	1.617588
H	−3.779199	0.911165	−1.379473
IM2			
0	1		
C	−2.303223	−1.330794	−0.096353
C	−3.408972	−0.588600	−0.005835
C	−3.599756	0.829112	−0.252161
C	−2.699610	1.708819	−0.641028
C	−0.416857	0.209417	0.241184
C	−0.985602	−0.797950	−0.415794
H	0.976720	0.351261	−2.228094
C	1.547120	0.294810	−1.312092
C	2.775378	−0.215955	−1.323070
C	0.911125	0.828231	−0.082123
C	3.541004	−0.280977	−0.093026
C	1.776015	0.793419	1.170645
C	3.094676	0.183944	1.080287
H	3.231856	−0.583879	−2.227357
H	−0.906374	0.643507	1.103457
O	−1.987453	2.560968	−0.959289
H	−0.445305	−1.286611	−1.218343
H	−2.401797	−2.397856	0.034563
H	0.712496	1.894216	−0.250981
O	1.355943	1.258736	2.205764
Cl	5.099546	−0.997459	−0.209722
Cl	−4.898933	−1.376112	0.408562
H	−4.593203	1.242315	−0.172472
H	3.679477	0.129266	1.983340
IM3			
0	1		
C	1.677557	−1.348313	−0.279063
C	2.796595	−0.457575	−0.054183
C	2.685328	0.878014	−0.024968
C	1.383109	1.499815	−0.210290
C	0.251791	0.621355	−0.707512

Table S2. *Cont.*

IM	x	y	z
C	0.479666	−0.838952	−0.557952
C	−1.294119	0.668696	1.288918
C	−2.418646	−0.042450	1.405382
C	−1.097792	1.072686	−0.136638
C	−3.016355	−0.156126	0.082013
C	−2.271306	0.472052	−0.830968
O	1.205831	2.683469	−0.020550
H	−0.372716	−1.486114	−0.710639
H	−0.615888	0.939546	2.080682
H	1.842607	−2.409934	−0.197481
H	−2.842181	−0.466871	2.299373
H	−1.118139	2.163321	−0.211863
H	0.229931	0.824528	−1.786559
H	3.520457	1.521193	0.197008
H	−2.474315	0.555182	−1.884570
Cl	4.319553	−1.206426	0.223814
Cl	−4.488457	−0.989093	−0.192637
IM4			
0	1		
C	−1.920484	−1.234139	−0.049784
C	−3.024202	−0.484106	−0.062351
C	−3.179466	0.945351	−0.263374
C	−2.240896	1.830955	−0.528301
C	−0.061388	0.260428	0.574257
C	−0.562873	−0.720667	−0.172148
C	2.192001	0.261690	−0.608772
C	3.326604	−0.157321	−0.045682
C	1.321423	0.817661	0.466203
C	3.300516	0.044899	1.396217
C	2.132534	0.606827	1.711536
O	−1.496617	2.689250	−0.736480
H	−2.046945	−2.304460	0.016126
H	−4.173305	1.365180	−0.268003
H	1.232582	1.901389	0.307933
H	1.790423	0.879737	2.695493
H	−0.682311	0.702012	1.345400
H	1.946135	0.248221	−1.655873
H	4.103830	−0.223941	2.060374
H	0.078809	−1.212323	−0.893035
Cl	4.678209	−0.837601	−0.850832
Cl	−4.559320	−1.274122	0.131810
IM5			
0	1		
C	2.453969	0.559007	−1.111425
C	1.585515	1.572888	−1.112511

Table S2. *Cont.*

IM	x	y	z
C	0.755796	1.527924	0.129739
C	1.253102	0.309778	0.833585
C	2.236081	−0.217638	0.101783
H	3.198704	0.326488	−1.853340
H	1.486109	2.334510	−1.868286
C	−2.453859	0.558969	1.111586
C	−2.236085	−0.217642	−0.101693
C	−1.253241	0.309798	−0.833585
C	−0.755963	1.528110	−0.129902
C	−1.585444	1.572951	1.112523
H	−3.197841	0.325935	1.854095
H	−1.485628	2.334184	1.868658
H	−0.989305	2.412513	−0.733244
H	0.989184	2.412309	0.733070
H	−0.863581	−0.061030	−1.765087
Cl	−3.152140	−1.619065	−0.469991
H	0.863225	−0.061031	1.765000
Cl	3.152134	−1.619096	0.469904
IM6			
0	1		
C	−1.781026	−0.395030	−1.404205
C	−2.696900	−0.249580	−0.293449
C	−2.326018	−0.336495	0.991325
C	−0.929569	−0.558313	1.330474
C	0.018043	−0.907580	0.202090
C	−0.501998	−0.678392	−1.168496
C	1.601645	1.088437	0.043815
C	2.739483	1.545106	−0.480232
C	1.413919	−0.335426	0.440968
C	3.826836	0.632941	−0.742983
C	2.486089	−1.277311	−0.089675
C	3.735323	−0.689676	−0.543738
O	2.289969	−2.472802	−0.098712
H	4.536881	−1.368389	−0.785635
H	4.741908	1.050846	−1.136176
H	2.859337	2.592776	−0.701148
H	0.158679	−1.993075	0.299421
O	−0.529571	−0.513088	2.473661
H	0.188886	−0.794699	−1.991458
H	−2.161996	−0.264589	−2.403519
Cl	−4.336218	0.076444	−0.701099
Cl	0.309829	2.169527	0.387492
H	−3.018783	−0.184357	1.801944
H	1.545554	−0.364987	1.532942

Table S2. *Cont.*

IM	x	y	z
IM7			
0	1		
C	1.899497	1.409144	−0.319807
C	2.857289	0.369980	−0.014175
C	2.570786	−0.704604	0.732237
C	1.233406	−0.885590	1.277799
C	0.246059	0.267865	1.128043
C	0.670826	1.344442	0.187353
C	−1.529632	−0.878366	−0.225950
C	−2.868773	−1.386498	−0.480656
C	−1.144753	−0.228054	0.867684
C	−4.007386	−0.694381	−0.372764
C	−3.349005	1.659535	0.112514
C	−4.236307	0.702190	−0.064219
O	−2.635489	2.549788	0.295292
H	−5.251705	1.068167	−0.029770
H	−4.921456	−1.235552	−0.565079
H	−2.927829	−2.415870	−0.801508
H	0.245379	0.726400	2.122987
O	0.917042	−1.887578	1.875798
H	−0.057238	2.105619	−0.047017
H	2.201287	2.209897	−0.974554
Cl	−0.367940	−1.267305	−1.458523
Cl	4.427382	0.559917	−0.693930
H	3.287708	−1.488208	0.912454
H	−1.891133	−0.096507	1.637118
IM8			
0	1		
C	1.388064	−0.477933	−1.369643
C	2.291895	−0.009277	−0.338944
C	1.942834	0.867666	0.611163
C	0.586406	1.389099	0.653391
C	−0.335312	1.066475	−0.510476
C	0.154836	0.017425	−1.440269
C	−1.963587	−0.628172	0.423456
C	−2.985062	−1.190993	−0.227761
C	−1.761023	0.784262	−0.020086
C	−3.522400	−0.202856	−1.153362
C	−2.821660	0.932997	−1.058438
H	−4.366323	−0.377377	−1.800155
O	0.199870	2.101273	1.553661
H	−0.537869	−0.333458	−2.192756
H	1.739500	−1.232865	−2.053483
H	−3.347852	−2.195677	−0.096631
H	−1.943100	1.466900	0.815145

Table S2. *Cont.*

IM	x	y	z
H	−0.375339	2.007370	−1.071491
H	2.620129	1.172385	1.391250
H	−2.989355	1.845420	−1.606117
Cl	3.880456	−0.668169	−0.369517
Cl	−0.953908	−1.358599	1.591327
IM9			
0	1		
C	−1.682958	−0.992532	−0.765272
C	−2.658974	−0.305093	−0.169529
C	−2.577634	0.861665	0.690848
C	−1.498957	1.553968	0.998946
C	0.331549	−0.642033	0.600688
C	−0.263661	−0.710390	−0.586802
C	2.553116	0.175747	−0.330872
C	3.619465	−0.594314	−0.563057
C	1.775225	−0.328479	0.837603
C	3.617196	−1.673247	0.415810
C	2.569183	−1.538484	1.234065
H	4.359925	−2.452615	0.456912
O	−0.616292	2.209816	1.349467
H	−1.964580	−1.773255	−1.455813
H	4.362169	−0.436678	−1.326103
H	−3.487110	1.280982	1.091248
H	1.838144	0.421210	1.634586
H	2.289697	−2.186787	2.046705
H	−0.253666	−0.838004	1.491349
H	0.324811	−0.567232	−1.484933
Cl	2.113567	1.607595	−1.155231
Cl	−4.304176	−0.782124	−0.457961
IM10			
0	1		
C	−2.703394	−1.489426	−0.728145
C	−2.172543	−1.628278	0.490509
C	−1.462214	−0.371878	0.882102
C	−1.690015	0.489077	−0.319985
C	−2.407351	−0.158885	−1.241469
H	−3.271627	−2.231836	−1.263882
H	−2.217177	−2.500392	1.121558
H	−2.719027	0.234251	−2.193549
C	1.909957	0.694620	1.123435
C	1.942785	−0.298137	0.058504
C	0.851872	−1.065560	0.086983
C	0.009989	−0.621415	1.236875
C	0.769562	0.536483	1.798413
H	2.685272	1.420095	1.299867

Table S2. *Cont.*

IM	x	y	z
H	0.433321	1.116849	2.641361
H	0.016875	−1.410576	1.997396
H	−1.954553	0.079097	1.748359
Cl	−1.121487	2.096173	−0.427298
H	0.601300	−1.871302	−0.579944
Cl	3.247001	−0.411220	−1.048848
IM11			
0	1		
C	−1.885426	−0.658479	−1.106148
C	−2.915443	−0.331696	−0.322458
C	−2.952784	0.494088	0.870494
C	−1.966847	1.206274	1.378288
C	0.124428	−0.477774	0.301491
C	−0.502524	−0.275439	−0.853073
C	2.179070	0.760954	−0.420149
C	3.163596	0.314188	−1.197467
C	1.537994	−0.072875	0.629798
C	3.683804	−1.022815	−1.002963
C	2.280609	−1.368567	0.946040
C	3.278786	−1.826001	−0.010170
H	4.451424	−1.359819	−1.683917
H	3.594698	0.945286	−1.957345
H	−0.371803	−1.002090	1.108054
O	−1.160357	1.840642	1.905678
H	0.029462	0.186157	−1.676617
H	−2.099016	−1.202870	−2.013598
H	1.499981	0.494168	1.562811
O	1.992645	−1.987644	1.943575
H	3.701171	−2.804442	0.151941
H	−3.887284	0.614431	1.395244
Cl	1.570689	2.361753	−0.588334
Cl	−4.495896	−0.899750	−0.762413
IM12			
0	1		
C	−2.077191	1.490993	0.751734
C	−3.141900	0.570871	1.071208
C	−3.268643	−0.630995	0.490248
C	−2.304532	−1.072383	−0.501043
C	−1.086675	−0.194752	−0.778099
C	−1.132164	1.141037	−0.121232
C	0.370475	−1.160544	1.088261
C	1.642127	−0.876580	1.379003
C	0.155022	−1.027839	−0.384659
C	2.319140	−0.516580	0.140539
C	1.475758	−0.561945	−0.892048

Table S2. *Cont.*

IM	x	y	z
H	1.696759	−0.342972	−1.921550
O	−2.405114	−2.128684	−1.085976
H	−4.083285	−1.300582	0.713759
H	−0.396445	−1.468976	1.779619
H	−3.872773	0.890642	1.799451
H	−2.065865	2.466891	1.208196
H	2.120299	−0.908206	2.342933
H	−1.051957	−0.055534	−1.859738
H	−0.043351	−2.020920	−0.803623
Cl	0.093180	2.261884	−0.561749
Cl	3.979115	−0.097094	0.089119
IM13			
0	1		
C	−2.424306	−1.624438	0.356994
C	−3.620182	−1.025963	0.341099
C	−3.975537	0.360006	0.114921
C	−3.188529	1.369534	−0.199931
C	−0.761770	0.121890	0.888676
C	−1.139392	−0.956856	0.206705
C	0.856151	1.448825	−0.579236
C	2.121436	1.162205	−0.891956
C	0.528005	0.876174	0.767531
C	2.676622	0.343679	0.174536
C	1.774624	0.129810	1.133272
O	−2.571023	2.310550	−0.459207
H	−2.394700	−2.699078	0.459359
H	2.665552	1.464299	−1.770157
H	−5.019047	0.637770	0.124184
H	0.462286	1.731002	1.449508
H	1.901415	−0.444343	2.033311
H	−1.472028	0.472150	1.625897
H	0.158384	2.033360	−1.153877
H	−4.481332	−1.659345	0.492212
Cl	4.287107	−0.243756	0.139007
Cl	−0.071524	−1.712092	−0.930916
IM14			
0	1		
C	−2.201392	−1.244495	−1.005587
C	−3.288995	−0.531513	−0.380816
C	−3.123613	0.282512	0.671582
C	−1.799621	0.515317	1.223232
C	−0.665729	−0.374228	0.731817
C	−0.969831	−1.138586	−0.507854
H	−4.273491	−0.655973	−0.807329
O	−1.581007	1.352007	2.070879

Table S2. *Cont.*

IM	x	y	z
C	0.970128	1.138523	−0.507830
C	2.201700	1.243326	−1.005767
C	0.665502	0.374737	0.732055
C	3.288810	0.529565	−0.381032
C	1.799088	−0.514659	1.224434
C	3.123007	−0.283781	0.671822
O	1.580205	−1.349783	2.073572
H	3.936642	−0.844390	1.102292
H	4.273268	0.652808	−0.807982
H	−2.392257	−1.853389	−1.873623
H	2.392906	1.851782	−1.874035
H	−3.937638	0.842558	1.102051
Cl	0.344750	−1.981363	−1.230924
Cl	−0.343950	1.981816	−1.231235
H	0.550664	1.121101	1.531609
H	−0.551577	−1.120292	1.531772
IM15			
0	1		
C	2.154801	−1.702648	0.617687
C	3.353159	−1.268895	0.207384
C	3.774867	−0.055037	−0.453638
C	3.080112	1.009198	−0.806805
C	0.433888	−0.468643	−0.671841
C	0.866482	−1.057698	0.438487
C	−1.375004	1.133448	0.136430
C	−2.637578	1.208382	0.554646
C	−0.918075	0.140996	−0.883063
C	−3.605128	0.237287	0.105577
C	−1.943552	−0.952842	−1.208963
C	−3.300506	−0.774112	−0.719529
H	−2.938885	1.979508	1.244030
H	1.082257	−0.528378	−1.532027
O	2.558198	1.970732	−1.171686
H	2.127819	−2.635429	1.160593
H	−0.866400	0.712485	−1.817070
O	−1.610953	−1.894414	−1.889745
H	4.827617	0.072152	−0.660854
H	−4.024814	−1.514694	−1.017985
H	−4.614742	0.335038	0.476857
H	4.183665	−1.926233	0.416811
Cl	−0.206096	2.270238	0.673877
Cl	−0.185391	−1.199743	1.815703
IM16			
0	1		
C	1.831203	1.493481	−0.761002

Table S2. *Cont.*

IM	x	y	z
C	2.921145	0.566694	−0.574369
C	2.849085	−0.497605	0.236869
C	1.634556	−0.780731	0.977929
C	0.494121	0.236970	0.912823
C	0.687855	1.317113	−0.099552
C	−1.319212	−0.970691	−0.451229
C	−2.607913	−0.696946	−0.627766
C	−0.846369	−0.472895	0.868993
C	−3.070988	0.038893	0.543783
C	−2.067089	0.220607	1.389650
H	−4.091889	0.341189	0.708731
O	1.504365	−1.770439	1.663705
H	3.826712	0.745274	−1.135605
H	1.945857	2.318650	−1.443953
H	−3.200523	−0.970962	−1.481644
H	−0.732184	−1.353231	1.518040
H	0.549030	0.740561	1.885379
H	−2.100228	0.717617	2.343505
H	3.658922	−1.201321	0.337875
Cl	−0.614917	2.418639	−0.297621
Cl	−0.263258	−1.824733	−1.498776
IM17			
0	1		
C	−2.065252	−1.711060	−0.086597
C	−3.205169	−1.014964	0.013815
C	−3.464700	0.393725	0.202910
C	−2.635697	1.405117	0.382636
C	−0.303866	−0.308111	0.931949
C	−0.714270	−1.190325	0.023432
C	1.779554	0.766078	−0.133535
C	3.036110	0.316200	−0.150117
C	1.059160	0.287320	1.089358
C	3.219663	−0.550689	1.003702
C	2.090422	−0.604404	1.717055
H	4.137987	−1.065525	1.232966
O	−2.014147	2.356152	0.583443
H	−4.115626	−1.586447	−0.088016
H	−2.144335	−2.764904	−0.307195
H	3.779918	0.542677	−0.893989
H	−4.489480	0.732679	0.148133
H	0.955722	1.164658	1.736779
H	1.913268	−1.161994	2.620326
H	−1.036901	−0.034665	1.677759
Cl	1.032974	1.786351	−1.278715
Cl	0.398607	−1.869235	−1.119047

Table S2. *Cont.*

IM	x	y	z
IM18			
0	1		
C	2.602189	1.012684	0.350542
C	1.665761	0.997232	1.303697
C	0.750875	−0.167732	1.098142
C	1.303384	−0.767371	−0.157591
C	2.376066	−0.088826	−0.572688
H	3.403279	1.726768	0.256764
H	1.557023	1.692786	2.118903
H	2.976157	−0.321502	−1.435218
C	−2.601213	−1.013675	0.349927
C	−2.376230	0.089500	−0.571418
C	−1.303721	0.768221	−0.156015
C	−0.749221	0.166099	1.097856
C	−1.665142	−0.998239	1.303444
H	−3.403402	−1.726593	0.257045
H	−1.556708	−1.694364	2.118294
H	−2.978068	0.324714	−1.432095
H	−0.918754	0.875434	1.914973
Cl	−0.662668	2.177049	−0.881558
H	0.919509	−0.877910	1.914081
Cl	0.661755	−2.176267	−0.882449
IM19			
0	2		
C	2.695189	1.263291	−0.560216
C	1.485449	1.787618	−0.260737
C	0.664650	0.745426	0.351429
C	1.402452	−0.414805	0.415182
C	2.649768	−0.111363	−0.142189
H	3.541781	1.746676	−1.012063
H	1.162332	2.800849	−0.430319
C	−2.372554	−0.648360	1.260204
C	−2.634343	−0.087294	−0.058670
C	−1.715609	0.824261	−0.379975
C	−0.753293	0.933523	0.768144
C	−1.271084	−0.071729	1.744386
H	−2.986733	−1.396176	1.731824
H	−0.802971	−0.266393	2.694448
H	−0.857024	1.933167	1.207176
H	−1.646729	1.391263	−1.290756
Cl	−3.966227	−0.580274	−1.018255
H	1.091406	−1.366080	0.805076
Cl	3.942357	−1.194239	−0.307083
IM20			
0	2		

Table S2. *Cont.*

IM	x	y	z
C	1.740188	−1.395079	−0.000252
C	0.452882	−1.018354	−0.000293
C	0.400890	0.446254	−0.000141
C	1.786614	0.901179	0.000035
C	2.558393	−0.197738	−0.000058
H	2.129185	−2.398207	−0.000530
H	−0.409281	−1.661019	−0.000440
C	−1.879327	0.555223	1.144065
C	−2.537340	0.145465	−0.000010
C	−1.879531	0.555610	−1.143956
C	−0.674570	1.277699	−0.741044
C	−0.674515	1.277359	0.741274
H	−2.168033	0.332062	2.155528
H	−0.289535	2.095468	1.327736
H	−0.289549	2.095552	−1.327807
H	−2.168569	0.333276	−2.155507
Cl	−3.957489	−0.819595	0.000065
H	2.111284	1.926633	0.000260
Cl	4.270570	−0.239786	0.000114
IM21			
0	2		
C	2.703075	1.056763	−0.000291
C	1.628173	1.861685	0.000184
C	0.425417	1.032672	−0.000290
C	0.888312	−0.356288	−0.000506
C	2.227725	−0.314255	−0.000006
H	3.740149	1.343319	−0.000427
H	1.623826	2.938755	0.000379
C	−1.793881	0.501952	1.143718
C	−2.312511	−0.074444	0.000105
C	−1.794182	0.502384	−1.143477
C	−0.836743	1.531599	−0.741395
C	−0.836626	1.531482	0.741775
H	−2.008601	0.206800	2.155162
H	−0.697550	2.424568	1.328898
H	−0.698337	2.425325	−1.327764
H	−2.009390	0.207787	−2.154979
Cl	−3.407551	−1.396169	−0.000062
H	0.263722	−1.230347	−0.000898
Cl	3.289529	−1.660155	0.000105
IM22			
0	2		
C	1.713100	−0.244719	1.354100
C	0.424308	0.095603	1.204064
C	0.201613	0.453374	−0.198492

Table S2. *Cont.*

IM	x	y	z
C	1.488451	0.288737	−0.867575
C	2.363761	−0.121046	0.064108
H	2.206459	−0.560128	2.256663
H	−0.333838	0.109774	1.966194
C	−2.625771	0.974311	0.592835
C	−2.271544	−0.136089	−0.155986
C	−1.095745	0.145315	−0.975932
C	−0.765199	1.560428	−0.675388
C	−1.768274	2.011644	0.294821
H	−3.422195	0.996673	1.317085
H	−1.785470	2.993512	0.734683
H	−0.357503	2.232085	−1.413181
H	−0.969502	−0.313163	−1.942739
Cl	−3.024938	−1.658957	−0.105698
H	1.680487	0.468785	−1.910472
Cl	4.024547	−0.464154	−0.178277
IM23			
0	2		
C	2.302557	0.846386	−1.049476
C	1.119686	1.459477	−1.215457
C	0.155429	0.873282	−0.286463
C	0.881395	−0.160503	0.452826
C	2.137999	−0.154999	−0.012811
H	3.222819	1.038204	−1.572719
H	0.891379	2.253534	−1.906455
C	−2.209196	0.118138	1.361093
C	−2.117858	−0.219240	0.020372
C	−1.323046	0.765691	−0.709688
C	−0.947355	1.770694	0.316349
C	−1.545483	1.307882	1.572423
H	−2.690929	−0.483303	2.113049
H	−1.429954	1.808761	2.517722
H	−0.852450	2.821289	0.094984
H	−1.542625	1.004026	−1.737349
Cl	−2.761194	−1.613792	−0.707380
H	0.471079	−0.791945	1.218804
Cl	3.420365	−1.168057	0.506024
IM24			
0	2		
C	2.379274	1.362963	0.002549
C	1.067861	1.605550	0.002841
C	0.293044	0.308801	0.000387
C	1.399859	−0.717289	−0.001492
C	2.571301	−0.081279	−0.000163
H	3.182193	2.080175	0.004013

Table S2. *Cont.*

IM	x	y	z
H	0.578621	2.564563	0.004646
C	−1.870728	−0.008671	1.224329
C	−2.558836	−0.108360	−0.000156
C	−1.870894	−0.004031	−1.224380
C	−0.533511	0.193927	−1.245266
C	−0.533335	0.189255	1.245758
H	−2.424480	−0.093011	2.146298
H	−0.004497	0.264504	2.183656
H	−0.004846	0.272761	−2.182967
H	−2.424808	−0.084841	−2.146569
Cl	−4.256281	−0.359579	−0.000522
H	1.224828	−1.778107	−0.003610
Cl	4.127386	−0.797552	−0.001355
IM25			
0	2		
C	2.136953	0.124350	1.388782
C	0.860150	0.479323	1.531638
C	0.150093	0.436538	0.197026
C	1.253462	−0.015301	−0.729993
C	2.367972	−0.179289	−0.017651
H	2.892494	0.061514	2.153179
H	0.355869	0.766815	2.438241
C	−2.609776	1.077255	−0.415566
C	−2.231877	−0.232559	−0.063977
C	−0.956934	−0.571566	0.228564
C	−0.322316	1.805671	−0.182500
C	−1.619236	2.075645	−0.464907
H	−3.638902	1.297726	−0.641835
H	−1.906140	3.080306	−0.736336
H	0.430725	2.577676	−0.224188
H	−0.697481	−1.584328	0.492735
Cl	−3.469749	−1.444371	−0.012386
H	1.116400	−0.163258	−1.786000
Cl	3.897754	−0.675443	−0.606690
IM26			
0	2		
C	1.620123	2.075518	−0.465596
C	0.323183	1.806760	−0.181977
C	0.956369	−0.570760	0.229750
C	2.231334	−0.232972	−0.064143
C	2.609881	1.076377	−0.416826
H	1.907709	3.079908	−0.737317
H	−0.429117	2.579514	−0.223040
H	3.639013	1.296038	−0.643863
C	−2.137133	0.124506	1.388752

Table S2. *Cont.*

IM	x	y	z
C	−2.367555	−0.179064	−0.017798
C	−1.253043	−0.013906	−0.729859
C	−0.150111	0.438115	0.197667
C	−0.860673	0.480572	1.531974
H	−2.892778	0.060729	2.152969
H	−0.356805	0.768094	2.438797
H	0.696316	−1.583063	0.495071
Cl	3.468416	−1.445505	−0.012052
H	−1.115575	−0.161201	−1.785902
Cl	−3.896829	−0.676313	−0.607265
IM27			
0	2		
C	−2.611250	1.128337	0.412200
C	−1.459161	1.881066	0.486944
C	−0.306276	1.082251	0.040832
C	−0.870598	−0.259369	−0.335373
C	−2.306646	−0.119697	−0.104583
H	−3.592504	1.437600	0.729143
H	−1.381649	2.889039	0.857690
C	2.019998	1.028571	−0.722750
C	2.254655	−0.176261	0.031324
C	1.346656	−0.688005	0.875015
C	−0.012460	−0.184741	0.907836
C	0.841457	1.659383	−0.646832
H	2.844397	1.456968	−1.269035
H	0.716447	2.632112	−1.098251
H	−0.548321	−0.265807	1.842819
H	1.626003	−1.466274	1.567941
Cl	3.848410	−0.845453	−0.048754
H	−0.482270	−0.821327	−1.170427
Cl	−3.410784	−1.388166	−0.370515
IM28			
0	2		
C	2.404895	1.207233	−0.073070
C	1.228583	1.906055	−0.052789
C	−0.000043	1.265375	0.137932
C	1.252580	−0.894966	0.297455
C	2.377355	−0.217858	0.031790
H	3.345352	1.701358	−0.250885
H	1.237124	2.968406	−0.249349
C	−1.252952	−0.894848	0.298779
C	−2.377426	−0.217554	0.031979
C	−2.404762	1.207207	−0.074044
C	−1.228161	1.906011	−0.053804
C	−0.000008	−0.152679	0.623749

Table S2. *Cont.*

IM	x	y	z
H	−1.251007	−1.971853	0.357216
H	0.000954	−0.066797	1.734466
H	−1.236700	2.968283	−0.250755
H	−3.345213	1.701510	−0.251326
Cl	−3.863503	−1.059146	−0.248515
H	1.250120	−1.972048	0.354746
Cl	3.863445	−1.059248	−0.248660
IM29			
0	2		
C	−2.006359	1.267899	0.308405
C	−0.703742	1.574835	0.255925
C	−0.200770	−0.830265	−0.465660
C	−1.638124	−0.991896	−0.536921
C	−2.465068	−0.043321	−0.072589
H	−2.744102	2.019161	0.538158
H	−0.379458	2.594335	0.400324
C	2.555390	−0.014133	0.081572
C	1.915092	−0.924664	0.900681
C	0.481342	−0.642035	0.875724
C	0.320709	0.570010	0.003015
C	1.674111	0.901436	−0.458037
H	1.920143	1.687851	−1.149852
H	0.393162	−1.338722	−1.211725
H	−0.142029	−0.773494	1.746860
H	−2.037423	−1.877218	−1.006439
Cl	−4.177497	−0.290681	−0.092935
Cl	4.237005	−0.049450	−0.265810
H	2.382585	−1.736897	1.428651
IM30			
0	2		
C	−0.767786	−1.716274	0.113703
C	0.206166	−0.679208	0.562315
C	−1.593976	0.980761	0.090769
C	−2.487647	−0.050517	−0.029472
C	−2.054349	−1.409835	−0.107713
H	−0.424420	−2.737164	0.036796
H	0.186455	−0.730762	1.674226
H	−2.772244	−2.168415	−0.375780
C	2.075120	1.417853	0.082636
C	2.483572	0.049164	0.022719
C	1.622284	−0.962768	0.184542
C	−0.231327	0.717666	0.236785
C	0.743366	1.720305	0.151039
H	2.818783	2.191404	−0.011291
H	0.426230	2.750524	0.079045

Table S2. *Cont.*

IM	x	y	z
H	−1.936294	2.000942	0.006565
Cl	−4.164835	0.278009	−0.231803
H	1.948487	−1.988774	0.118505
Cl	4.151921	−0.261575	−0.319491
IM31			
0	2		
C	−0.846194	−1.637967	1.041678
C	0.295907	−0.746851	1.027542
C	−1.303816	0.944897	−0.055186
C	−2.222867	−0.024742	−0.115494
C	−2.000710	−1.342141	0.423983
H	−0.761122	−2.557177	1.602182
H	0.978402	−0.789902	1.864325
H	−2.824025	−2.037792	0.425894
C	2.271600	1.384736	0.514364
C	2.271889	0.196409	−0.196621
C	0.924716	−0.360136	−0.294523
C	0.055983	0.657800	0.388127
C	0.975865	1.709143	0.853807
H	3.154592	1.940820	0.779033
H	0.662296	2.573039	1.414900
H	−1.549530	1.957480	−0.332994
Cl	−3.809602	0.309340	−0.720445
H	0.590686	−0.887674	−1.174308
Cl	3.645747	−0.596733	−0.815149
IM32			
0	2		
C	1.558553	−0.579839	1.146741
C	0.205924	−0.026163	1.094301
C	0.192123	0.906340	−0.086450
C	1.535870	0.807636	−0.672664
C	2.278061	−0.082348	0.076610
H	1.912913	−1.292346	1.870501
H	−0.347173	0.212288	1.989671
C	−2.674036	0.898617	0.091852
C	−2.056304	−0.279808	−0.071990
C	−0.615216	−0.427944	−0.114267
C	−0.592129	2.137218	−0.105616
C	−1.921900	2.126956	0.053111
H	−3.749685	0.936699	0.150677
H	−2.468845	3.056419	0.078532
H	−0.060455	3.064189	−0.256838
H	−0.218861	−1.237508	−0.709563
Cl	−2.965576	−1.733845	−0.284470
H	1.858603	1.308083	−1.568765

Table S2. *Cont.*

IM	x	y	z
Cl	3.883684	−0.556262	−0.305176
IM33			
0	2		
C	1.921498	2.273938	−0.143327
C	0.560245	2.264958	0.011404
C	−0.145473	1.068557	0.155678
C	2.035316	−0.121377	0.011732
C	2.651686	1.052068	−0.202145
H	2.448313	3.203354	−0.286761
H	0.004391	3.189746	−0.039786
H	3.706187	1.068178	−0.427080
C	−1.480505	−1.394353	−0.167475
C	−2.197192	−0.170396	0.010281
C	−1.543718	1.024901	0.112642
C	0.600422	−0.210646	0.426328
C	−0.147268	−1.419570	−0.037985
H	−2.029947	−2.281838	−0.438295
H	0.403670	−2.333678	−0.188003
H	0.634340	−0.306407	1.534091
Cl	2.940186	−1.589052	−0.012729
H	−2.101909	1.948487	0.088351
Cl	−3.916369	−0.216613	−0.064055
IM34			
0	2		
C	1.996712	0.732885	1.009386
C	0.545160	0.860845	0.893182
C	0.127960	−0.150580	−0.134554
C	1.372758	−0.788208	−0.582714
C	2.426190	−0.219468	0.104914
H	2.625900	1.317033	1.657519
H	−0.079949	1.059051	1.750377
C	−2.395707	1.209984	−0.242905
C	−2.299323	−0.208164	−0.008052
C	−1.138038	−0.870041	−0.051230
C	0.038216	1.380785	−0.439563
C	−1.299507	1.927167	−0.538347
H	−3.377435	1.651793	−0.296305
H	−1.405870	2.940174	−0.897368
H	0.798752	1.790588	−1.088698
H	−1.114696	−1.947679	−0.021823
Cl	−3.789783	−1.067432	0.180546
H	1.445280	−1.531587	−1.357117
Cl	4.075754	−0.610839	−0.169210
IM35			
0	2		

Table S2. *Cont.*

IM	x	y	z
C	−2.390291	1.195576	−0.186479
C	−1.253858	1.891145	−0.041135
C	−0.000050	−0.251657	0.216747
C	−1.223423	−0.923864	0.150434
C	−2.388748	−0.221717	0.002279
H	−3.310403	1.678559	−0.474255
H	−1.238919	2.959096	−0.200835
C	2.390350	1.195684	−0.185608
C	2.388606	−0.221840	0.002428
C	1.223537	−0.923956	0.150486
C	−0.000083	1.234679	0.431143
C	1.254033	1.891219	−0.040149
H	3.310631	1.678717	−0.472741
H	1.239299	2.959267	−0.199240
H	−0.000553	1.365791	1.535741
H	1.241567	−2.003090	0.150307
Cl	3.892101	−1.053757	−0.102868
H	−1.241616	−2.003004	0.150464
Cl	−3.892128	−1.053711	−0.102445
IM36			
0	2		
C	2.031544	1.820698	−0.825728
C	0.842631	2.213643	−0.350140
C	0.432386	−0.175663	0.483303
C	1.787923	−0.421717	0.031589
C	2.511243	0.472779	−0.656282
H	2.692008	2.538518	−1.286508
H	0.552689	3.252982	−0.377361
H	3.500205	0.214082	−0.998136
C	−2.289797	0.848995	0.946125
C	−2.011137	−0.062791	−0.058398
C	−0.642740	0.091627	−0.545775
C	−0.104242	1.264751	0.227763
C	−1.198410	1.670896	1.127370
H	−3.207356	0.879892	1.508310
H	−1.115842	2.469340	1.845086
H	0.113716	−0.672574	1.387939
Cl	2.454576	−1.956187	0.462473
H	−0.388213	−0.068254	−1.581766
Cl	−3.060671	−1.276360	−0.625798
IM37			
0	2		
C	−2.011326	1.441327	0.140350
C	−0.838108	2.139065	0.150268
C	0.406243	1.491968	0.150195

Table S2. *Cont.*

IM	x	y	z
C	−0.833329	−0.681657	0.131808
C	−1.972397	0.014794	0.044135
H	−2.962959	1.944907	0.110386
H	−0.858263	3.217655	0.090778
H	−0.824580	−1.754807	0.042468
C	2.776483	1.497106	−0.290773
C	2.802586	0.073654	−0.290195
C	1.699859	−0.629542	0.017138
C	0.437071	0.025002	0.481222
C	1.598641	2.171755	−0.094287
H	3.683937	2.035112	−0.512141
H	0.493889	−0.024429	1.591756
H	1.569634	3.247247	−0.191795
H	3.711980	−0.445866	−0.546880
Cl	−3.474091	−0.795029	−0.247308
Cl	1.755974	−2.350892	0.057676
IM38			
0	2		
C	−3.593759	−0.596840	−0.483370
C	−2.457814	−1.493264	−0.366835
C	−1.352428	−0.766773	−0.082052
C	−1.801545	0.616448	−0.017877
C	−3.174592	0.694452	−0.267131
H	−4.601396	−0.904985	−0.705427
H	−2.496750	−2.562324	−0.489236
H	−3.761689	1.593497	−0.282091
C	1.850595	−0.485993	1.339659
C	2.112388	−0.320700	−0.082325
C	1.075701	−0.738635	−0.810688
C	0.035060	−1.269672	0.125750
C	0.631100	−1.011170	1.477368
H	2.544261	−0.217976	2.117908
H	0.127764	−1.253313	2.397872
H	−0.003331	−2.356947	−0.005217
Cl	−0.801313	1.939680	0.313513
H	0.983981	−0.731854	−1.881996
Cl	3.581249	0.334837	−0.675090
IM39			
0	2		
C	1.474195	2.346907	−0.000474
C	0.334281	1.635597	0.000220
C	0.683506	0.213241	0.000128
C	2.143263	0.193033	−0.000018
C	2.615595	1.449912	−0.000465
H	1.549512	3.421100	−0.000865

Table S2. *Cont.*

H	−0.673017	2.012361	0.000330
H	3.651423	1.740343	−0.001056
C	−1.457674	−0.553878	−1.143662
C	−2.206633	−0.352316	0.000135
C	−1.458062	−0.551918	1.144232
C	−0.094900	−0.894070	0.741329
C	−0.094858	−0.895331	−0.740151
H	−1.798467	−0.424433	−2.155255
H	0.520459	−1.568178	−1.316644
H	0.519102	−1.565734	1.320669
Cl	3.045540	−1.261375	−0.000093
H	−1.799541	−0.422298	2.155573
Cl	−3.845642	0.159009	−0.000518
IM40			
0	2		
C	−3.430820	−0.114605	−0.000034
C	−2.583751	−1.155222	0.000116
C	−1.205816	−0.654264	−0.000014
C	−1.359155	0.810092	−0.000007
C	−2.667086	1.115828	−0.000087
H	−4.506152	−0.166341	−0.000177
H	−2.832675	−2.203340	−0.000001
H	−3.072731	2.112433	−0.000165
C	1.100230	−0.891735	1.142044
C	1.799405	−0.554700	−0.000064
C	1.100167	−0.891823	−1.142023
C	−0.159313	−1.516150	−0.742119
C	−0.159234	−1.515907	0.742298
H	1.400692	−0.679563	2.152359
H	−0.583593	−2.315796	1.327545
H	−0.583690	−2.315957	−1.327416
Cl	−0.101090	1.968707	0.000018
H	1.400508	−0.680081	−2.152465
Cl	3.287555	0.293620	−0.000038
IM41			
0	2		
C	−1.483456	2.284307	−0.300448
C	−0.341754	1.737199	0.149601
C	−0.531590	0.288986	0.236743
C	−1.903197	0.070792	−0.218607
C	−2.470274	1.246008	−0.533477
H	−1.657585	3.333336	−0.470217
H	0.570794	2.243824	0.407998
H	−3.473420	1.392220	−0.893289
C	1.173924	−0.072133	2.101276

Table S2. *Cont.*

IM	x	y	z
C	2.252821	0.129927	1.266994
C	1.922052	−0.290601	−0.011262
C	0.542715	−0.771347	−0.057506
C	0.053280	−0.623719	1.334052
H	1.129178	0.169972	3.148727
H	−0.654185	−1.311612	1.770025
H	3.193243	0.576754	1.540944
H	0.243239	−1.574887	−0.711580
Cl	2.924588	−0.209780	−1.381101
Cl	−2.609198	−1.485874	−0.301064
IM42			
0	2		
C	−3.012192	−0.689766	−0.795922
C	−2.017100	−1.511899	−0.430162
C	−0.888965	−0.714889	0.064003
C	−1.351321	0.676174	−0.072729
C	−2.597423	0.679400	−0.574495
H	−3.969446	−0.982554	−1.191988
H	−2.007497	−2.588473	−0.469111
H	−3.182487	1.558619	−0.780348
C	0.795505	−0.501144	2.027520
C	1.801063	0.044002	1.260822
C	1.665621	−0.402543	−0.042331
C	0.516758	−1.296881	−0.172322
C	−0.055659	−1.357826	1.196759
H	0.616629	−0.301353	3.069483
H	−0.491551	−2.255479	1.604847
H	2.531206	0.763475	1.588896
H	0.559164	−2.136720	−0.846807
Cl	2.640187	0.047110	−1.359062
Cl	−0.475113	2.093755	0.315425
IM43			
0	2		
C	−2.856894	−1.614335	0.000021
C	−1.544116	−1.862093	0.000227
C	−0.767117	−0.567046	0.000168
C	−1.902040	0.433941	−0.000043
C	−3.088614	−0.176629	−0.000113
H	−3.642089	−2.352307	−0.000010
H	−1.052732	−2.819571	0.000396
H	−4.050417	0.306571	−0.000303
C	1.390697	−0.239265	−1.224123
C	2.078627	−0.137374	−0.000007
C	1.390770	−0.238849	1.224179
C	0.054485	−0.439518	1.246011

Table S2. *Cont.*

IM	x	y	z
C	0.054410	−0.439943	−1.245790
H	1.943739	−0.153105	−2.146275
H	−0.475871	−0.513817	−2.182806
H	−0.475730	−0.512929	2.183097
Cl	−1.601926	2.112144	−0.000122
H	1.943879	−0.152322	2.146258
Cl	3.775337	0.116335	−0.000086
IM44			
0	2		
C	2.517903	−1.625211	−0.753044
C	1.296849	−1.867777	−0.269623
C	0.629243	−0.574422	0.137299
C	1.712648	0.419829	−0.224938
C	2.785593	−0.193421	−0.727531
H	3.215678	−2.363318	−1.112819
H	0.806904	−2.819355	−0.157498
H	3.692248	0.283979	−1.057125
C	−1.976522	−0.115432	1.310627
C	−1.803526	−0.123968	−0.086323
C	−0.604950	−0.334313	−0.673105
C	0.375032	−0.555517	1.611406
C	−0.855651	−0.336605	2.131375
H	−2.952111	0.060566	1.730389
H	−0.985857	−0.329588	3.202904
H	1.230750	−0.720507	2.247800
Cl	1.495825	2.091523	0.025626
H	−0.501601	−0.331016	−1.746364
Cl	−3.199692	0.147316	−1.075281
IM45			
0	2		
C	−2.504454	−1.637502	−0.754391
C	−1.282426	−1.869544	−0.268234
C	−0.626380	−0.570066	0.138793
C	−1.717641	0.414341	−0.224919
C	−2.784321	−0.207953	−0.729725
H	−3.195132	−2.381600	−1.115521
H	−0.784477	−2.816762	−0.154619
H	−3.694297	0.261762	−1.061187
C	1.981348	−0.117370	1.310182
C	1.806134	−0.118751	−0.086511
C	0.605942	−0.322196	−0.672391
C	−0.371377	−0.549902	1.612650
C	0.860692	−0.336786	2.131671
H	2.958096	0.053826	1.729236
H	0.991973	−0.331579	3.203078

Table S2. *Cont.*

IM	x	y	z
H	−1.227216	−0.711310	2.249779
Cl	−1.516158	2.087927	0.026487
H	0.500781	−0.313340	−1.745430
Cl	3.201169	0.155213	−1.076374
IM46			
0	2		
C	−3.251894	−0.670441	0.336078
C	−2.280210	−1.556552	−0.094022
C	−0.991517	−0.859337	−0.216315
C	−1.289278	0.565994	0.170159
C	−2.722470	0.596532	0.478141
H	−4.273158	−0.934573	0.554873
H	−2.408415	−2.609999	−0.277706
H	−3.234455	1.487178	0.798406
C	1.784865	−0.281671	0.148064
C	1.304426	−0.873305	−1.072969
C	0.016447	−1.214289	−1.203645
C	−0.443294	−0.243583	1.120325
C	0.993194	−0.070148	1.207808
H	1.419738	0.200352	2.160505
H	−0.955628	−0.523445	2.030245
Cl	−0.603112	1.940581	−0.648236
H	−0.311799	−1.772885	−2.066893
H	2.020808	−1.114480	−1.841073
Cl	3.486717	−0.004777	0.259995
IM47			
0	2		
C	1.853089	2.085457	0.453037
C	0.508406	1.508627	0.551882
C	0.672759	0.043327	0.260536
C	2.107476	−0.103477	−0.011561
C	2.758959	1.114367	0.083523
H	2.073339	3.127835	0.607215
H	−0.202285	1.836533	1.295118
H	3.803449	1.267665	−0.127282
C	−2.151749	−0.077003	−0.043410
C	−1.464448	−0.990596	0.832427
C	−0.127447	−0.988092	0.902445
C	−0.076448	0.980764	−0.741008
C	−1.505134	0.768037	−0.860193
H	−2.039480	1.278703	−1.646082
H	0.459902	1.260728	−1.636512
Cl	2.817876	−1.593038	−0.428205
H	0.395825	−1.768288	1.434130

Table S2. *Cont.*

IM	x	y	z
H	−2.045338	−1.738297	1.347074
Cl	−3.870710	−0.248924	−0.150603
IM48			
0	2		
C	0.589639	2.275414	0.010532
C	−0.105753	1.049130	0.492849
C	2.005583	−0.150438	0.005807
C	2.653670	1.043733	−0.166705
C	1.906695	2.256460	−0.244348
H	0.018423	3.189073	−0.057034
H	−0.065979	1.121662	1.604227
H	3.719135	1.051821	−0.325588
H	2.420608	3.159986	−0.534198
Cl	2.939240	−1.606584	−0.086976
C	−1.487756	−1.411127	0.120786
C	−2.188335	−0.169351	0.050790
C	−1.560417	1.007472	0.164070
C	0.617040	−0.230765	0.177834
C	−0.119238	−1.419498	0.147610
H	−2.045751	−2.331136	0.069548
H	0.406060	−2.359505	0.091613
H	−2.100219	1.938496	0.090937
Cl	−3.893301	−0.233215	−0.236251
IM49			
0	2		
C	−2.928761	−1.290268	−0.226018
C	−1.690493	−1.882173	−0.053502
C	−0.658174	−0.843363	0.079537
C	−1.403885	0.457333	−0.049560
C	−2.807689	0.084921	−0.256882
H	−3.861322	−1.824611	−0.299515
H	−1.486821	−2.937254	0.018918
H	−3.596128	0.807466	−0.376548
C	1.553577	0.619740	1.189667
C	1.707983	−0.274316	0.071797
C	0.705909	−1.025830	−0.396439
C	−0.840625	0.114828	1.307700
C	0.370757	0.734551	1.809992
H	2.430945	1.114400	1.573241
H	0.304501	1.278124	2.740295
H	−1.591979	−0.160843	2.034388
Cl	−0.783891	1.825324	−0.928672
H	0.886381	−1.781280	−1.144207
Cl	3.305234	−0.452886	−0.565700

Table S2. *Cont.*

IM	x	y	z
IM50			
0	2		
C	1.087027	−2.000440	1.071351
C	0.058689	−0.958309	1.159314
C	0.603612	0.220624	0.399936
C	1.902749	−0.254923	−0.095287
C	2.138184	−1.562748	0.294140
H	0.994589	−2.975376	1.518187
H	−0.527910	−0.807114	2.052272
H	2.997820	−2.142213	0.004075
C	−2.035970	1.326857	0.426171
C	−1.873487	0.114364	−0.122130
C	−0.606124	−0.589299	−0.148922
C	0.352927	1.605593	0.777772
C	−0.892720	2.090133	0.860008
H	−3.014394	1.778599	0.448148
H	−1.055794	3.105740	1.184876
H	1.210028	2.230281	0.977054
Cl	2.927098	0.705200	−1.056758
H	−0.427379	−1.255242	−0.980508
Cl	−3.196879	−0.698481	−0.880784
IM51			
0	2		
C	−0.651215	−2.403778	−0.258191
C	0.538024	−1.804815	−0.109259
C	−0.638598	0.402626	0.095277
C	−1.843176	−0.325634	0.052201
C	−1.871654	−1.684276	−0.076209
H	−0.703371	−3.443285	−0.543119
H	1.459914	−2.343595	−0.252366
H	−2.817826	−2.195840	−0.135061
C	1.861752	1.685446	−0.187434
C	1.852005	0.359036	0.022614
C	0.610465	−0.394628	0.380228
C	−0.561641	1.785873	−0.044381
C	0.647426	2.423526	−0.168328
H	2.795627	2.190791	−0.374739
H	0.682739	3.491581	−0.309320
H	−1.475854	2.352978	−0.108948
Cl	−3.351802	0.525856	0.057516
H	0.650771	−0.487048	1.488752
Cl	3.336959	−0.515611	0.059878
IM52			
0	2		

Table S2. *Cont.*

IM	x	y	z
C	2.207648	−1.597865	−0.968410
C	0.765461	−1.531822	−0.709537
C	0.517773	−0.171724	−0.124573
C	1.853963	0.435626	−0.066304
C	2.817593	−0.437408	−0.542352
H	2.705598	−2.454714	−1.388799
H	0.043324	−1.974540	−1.378608
H	3.875478	−0.237978	−0.545473
C	−2.057884	−1.118583	0.678224
C	−1.891461	0.095390	−0.079611
C	−0.690241	0.601570	−0.377185
C	0.366339	−1.456247	0.750477
C	−0.988690	−1.783061	1.144921
H	−3.056370	−1.405272	0.965716
H	−1.126346	−2.572784	1.868672
H	1.160153	−1.663123	1.453932
Cl	2.130471	1.995035	0.555312
H	−0.588491	1.587977	−0.800863
Cl	−3.331432	0.975858	−0.461692
IM53			
0	2		
C	−2.795312	1.462256	−0.247975
C	−1.662324	2.151376	−0.050550
C	−0.411944	−0.017737	0.147422
C	−1.658037	−0.656697	0.039018
C	−2.824129	0.040326	−0.118316
H	−3.703909	1.971570	−0.530038
H	−1.633315	3.225596	−0.156419
H	−3.752655	−0.494829	−0.227840
C	1.983727	1.456029	−0.123792
C	1.980037	0.034116	0.005568
C	0.813627	−0.680109	0.091069
C	−0.426906	1.462274	0.419017
C	0.839379	2.139245	0.016852
H	2.909750	1.954474	−0.361706
H	0.822833	3.213125	−0.095710
H	−0.457048	1.543911	1.528982
Cl	−1.726198	−2.386801	0.008776
H	0.839436	−1.756896	0.056727
Cl	3.487739	−0.789871	−0.084298
IM54			
0	2		
C	2.758036	−0.823360	−0.987552
C	2.142938	0.330048	−1.441022

Table S2. *Cont.*

IM	x	y	z
C	0.967317	0.629544	−0.608201
C	0.938982	−0.473325	0.418840
C	2.101189	−1.316731	0.122990
H	3.613430	−1.287826	−1.449170
H	2.428933	0.915796	−2.298365
H	2.350622	−2.193154	0.695299
C	−1.764848	1.238042	0.055459
C	−1.481804	−0.012076	−0.331043
C	−0.139796	−0.472570	−0.635265
C	0.515957	1.986266	−0.325949
C	−0.741786	2.251149	0.049550
H	−2.779677	1.508647	0.297421
H	−1.028276	3.262004	0.293768
H	1.240422	2.779271	−0.428836
Cl	0.524478	−0.213664	2.088265
H	−0.051953	−1.254153	−1.376437
Cl	−2.733338	−1.183542	−0.537707
IM55			
0	2		
C	−2.863557	−0.656198	0.435928
C	−2.581862	0.676534	0.381989
C	−1.263581	1.116765	0.147122
C	−0.578000	−1.240247	−0.051061
C	−1.862547	−1.610469	0.069321
H	−3.874154	−0.996825	0.595391
H	−3.377739	1.405801	0.421641
H	−2.156421	−2.613625	−0.195611
C	1.384410	1.885588	−0.424682
C	1.189207	0.662852	0.105071
C	−0.163054	0.129915	0.448206
C	−0.981906	2.386478	−0.337433
C	0.297469	2.762965	−0.672072
H	2.392934	2.223121	−0.603865
H	0.490802	3.735154	−1.095079
H	−1.804770	3.070052	−0.487897
Cl	0.563914	−2.335787	−0.716163
H	−0.163794	0.007195	1.548116
Cl	2.555714	−0.223975	0.669279
IM56			
0	2		
C	2.454163	2.100996	−0.143049
C	1.483859	1.875239	0.747472
C	1.204884	0.409798	0.832474
C	2.170448	−0.140675	−0.181001

Table S2. *Cont.*

IM	x	y	z
C	2.889841	0.840170	−0.729022
H	2.870256	3.061195	−0.399362
H	0.958714	2.605796	1.338719
H	3.662605	0.721948	−1.468790
C	−2.084443	−1.304371	0.603401
C	−2.348261	−0.032866	−0.003713
C	−1.193723	0.768526	0.005484
C	−0.214397	0.016139	0.602057
C	−0.783549	−1.279885	0.973958
H	−2.800225	−2.096348	0.724125
H	−0.235473	−2.070989	1.456306
H	1.514392	0.040910	1.817218
Cl	2.302406	−1.813097	−0.503784
H	−1.108839	1.765567	−0.384773
Cl	−3.851486	0.428009	−0.633381
IM57			
0	2		
C	−2.872262	−1.391446	−0.174836
C	−2.232614	−1.426703	1.054337
C	−1.315954	−0.290741	1.153964
C	−1.467417	0.451253	−0.117295
C	−2.460083	−0.292589	−0.901341
H	−3.566030	−2.135793	−0.529517
H	−2.346819	−2.180993	1.813525
H	−2.757705	−0.015941	−1.897390
C	1.653808	−1.390021	−0.701775
C	2.108381	−0.190934	−0.019149
C	1.069228	0.494257	0.479496
C	−0.136216	−0.257263	0.136647
C	0.317780	−1.433996	−0.617400
H	2.304470	−2.100022	−1.181541
H	−0.336519	−2.190417	−1.011698
H	−1.109742	0.204923	2.087986
Cl	−1.483052	2.195695	−0.221556
H	1.092802	1.424582	1.016678
Cl	3.761383	0.237408	0.100736
IM58			
0	2		
C	1.839563	2.193156	−0.234095
C	1.534475	1.853836	1.074641
C	1.328645	0.407308	1.163140
C	1.556240	−0.108620	−0.204771
C	1.873891	1.064975	−1.028376
H	1.991259	3.198613	−0.590540

Table S2. *Cont.*

IM	x	y	z
H	1.420146	2.533381	1.901355
H	2.054435	1.013947	−2.087668
C	−1.772608	−1.401020	0.578448
C	−2.086056	−0.109007	−0.008280
C	−0.979895	0.628835	−0.156768
C	0.135890	−0.178101	0.351944
C	−0.449216	−1.447331	0.790025
H	−2.498308	−2.165583	0.793756
H	0.114708	−2.264944	1.203053
H	1.590427	−0.158450	2.041936
Cl	2.407727	−1.597271	−0.546838
H	−0.903631	1.621030	−0.560047
Cl	−3.681527	0.350085	−0.433003
IM59			
0	2		
C	2.360963	−1.923731	−0.174721
C	1.049798	−1.757038	0.111073
C	1.372358	0.690365	−0.075708
C	2.682143	0.480276	−0.358411
C	3.205331	−0.820277	−0.414240
H	2.767944	−2.922442	−0.222549
H	0.402164	−2.601500	0.291458
H	3.320720	1.329890	−0.542976
H	4.247981	−0.968538	−0.641760
Cl	0.746415	2.292095	−0.014815
C	−1.595534	−0.207998	1.406213
C	−1.903884	−0.273540	−0.017034
C	−0.791420	−0.370533	−0.742567
C	0.391234	−0.408912	0.195440
C	−0.272408	−0.265440	1.547569
H	−2.336833	−0.125500	2.182500
H	0.293576	−0.240747	2.462902
H	−0.702344	−0.435159	−1.811925
Cl	−3.510220	−0.227097	−0.607734
IM60			
0	2		
C	−2.354332	−1.928824	−0.166185
C	−1.046686	−1.756206	0.131841
C	−1.374309	0.688582	−0.076092
C	−2.680339	0.472856	−0.371305
C	−3.198764	−0.829758	−0.424917
H	−2.758690	−2.928877	−0.207575
H	−0.399224	−2.597235	0.328284
H	−3.319894	1.319425	−0.566151

Table S2. *Cont.*

IM	x	y	z
H	−4.238389	−0.982720	−0.662904
Cl	−0.753929	2.292662	−0.016130
C	1.600160	−0.193873	1.405633
C	1.903516	−0.271619	−0.018031
C	0.788506	−0.375039	−0.738731
C	−0.390892	−0.405982	0.204239
C	0.277536	−0.249928	1.552257
H	2.344160	−0.104585	2.178566
H	−0.284747	−0.216908	2.469624
H	0.695201	−0.449187	−1.807100
Cl	3.507765	−0.230378	−0.614751
IM61			
0	2		
C	−1.792924	−0.451760	0.162720
C	−0.866196	−0.902671	−0.752435
C	0.478883	−0.766171	−0.179446
C	0.259432	−0.150326	1.186561
C	−1.190104	−0.017113	1.325873
H	0.873510	−0.430947	2.029370
H	−1.690810	0.406257	2.178146
C	3.251427	−0.164828	0.161234
C	2.395620	0.865283	0.249328
C	0.957321	0.689426	0.144487
C	1.527014	−1.738611	−0.466100
C	2.819501	−1.480879	−0.227106
H	4.307604	0.017948	0.285934
H	1.220390	−2.679574	−0.897443
H	3.565211	−2.236766	−0.417322
H	2.753795	1.873882	0.386264
H	−1.074080	−1.252687	−1.748146
Cl	−3.479103	−0.369026	−0.135362
Cl	0.126429	2.075367	−0.538020
IM62			
0	2		
C	−1.609691	−1.301467	0.857167
C	−0.255516	−1.278815	1.108998
C	0.368175	−0.166016	0.373497
C	−0.749629	0.499659	−0.378976
C	−1.925869	−0.300134	−0.046173
H	0.282734	−1.938308	1.766916
C	1.913781	2.201073	−0.140167
C	2.414337	0.895880	−0.485615
C	1.724104	−0.205926	−0.168051
C	−0.138156	1.251861	0.783277

Table S2. *Cont.*

IM	x	y	z
C	0.762345	2.360609	0.531191
H	2.542616	3.053830	−0.343664
H	0.494059	3.326606	0.931891
H	−0.752911	1.294214	1.671393
H	3.392962	0.804037	−0.927790
H	−0.599682	0.907030	−1.366526
Cl	2.417192	−1.771480	−0.392613
H	−2.324476	−1.968680	1.307412
Cl	−3.479462	0.052711	−0.645653
IM63			
0	2		
C	1.567422	−0.942630	1.177338
C	0.264616	−1.113815	1.581751
C	−0.664491	−0.791848	0.485119
C	0.224863	−0.379349	−0.670544
C	1.579683	−0.540782	−0.146532
H	−0.004888	−0.634083	−1.694096
C	−2.847793	0.265713	−1.024819
C	−1.832981	1.103494	−0.762588
C	−0.643318	0.679145	−0.042748
C	−1.917724	−1.517651	0.315945
C	−2.908809	−1.054538	−0.456479
H	−3.689398	0.623222	−1.597790
H	−2.024120	−2.445869	0.856989
H	−3.809060	−1.634468	−0.586448
H	−1.872393	2.137121	−1.069650
H	−0.063694	−1.397429	2.566974
Cl	0.104132	1.959185	0.895357
H	2.443477	−1.052268	1.792879
Cl	2.960060	−0.185225	−1.072269
IM64			
0	2		
C	−1.994135	−0.091189	0.076152
C	−0.947431	0.522516	−0.580882
C	0.306197	−0.072597	−0.101604
C	−0.096649	−1.104830	0.913149
C	−1.554324	−1.027838	0.992542
H	−1.024946	1.271654	−1.348164
H	−2.177906	−1.636273	1.623349
C	2.879006	−1.350148	−0.171422
C	2.755863	0.062434	0.079173
C	1.561232	0.664140	0.025816
C	0.461344	−1.595396	−0.410224
C	1.815553	−2.107035	−0.486505

Table S2. *Cont.*

IM	x	y	z
H	3.871168	−1.772775	−0.203675
H	1.952532	−3.117043	−0.842788
H	−0.273723	−2.015164	−1.082349
H	3.644903	0.654018	0.225151
H	0.515080	−1.281970	1.784170
Cl	1.436247	2.383020	0.116225
Cl	−3.649602	0.234463	−0.244041
IM65			
0	1		
C	−3.576244	−0.461597	−0.438530
C	−2.408679	−1.279040	−0.763902
C	−1.310433	−0.748731	−0.216035
C	−1.730208	0.453893	0.578668
C	−3.196108	0.564057	0.323586
H	−4.579381	−0.669495	−0.771665
H	−2.438154	−2.181131	−1.354177
H	−3.803057	1.357521	0.722274
C	1.893015	−1.008760	1.152306
C	2.139622	−0.291874	−0.091192
C	1.103069	−0.410161	−0.920845
C	0.079290	−1.286687	−0.266751
C	0.689131	−1.580114	1.070827
H	2.590221	−1.056918	1.971304
H	0.208125	−2.189671	1.817227
H	0.038217	−2.227415	−0.827000
H	−1.527186	0.290168	1.636613
Cl	−0.857853	1.948949	0.150030
H	0.994635	0.025937	−1.897368
Cl	3.588550	0.577233	−0.377441
IM66			
0	2		
C	3.643486	−0.348104	−0.224910
C	2.732221	0.560339	−0.763960
C	1.494484	0.267614	−0.220952
C	1.646571	−0.866573	0.689577
C	2.946900	−1.232540	0.681162
H	4.697858	−0.389708	−0.443991
H	2.948387	1.342760	−1.469811
H	0.840147	−1.306945	1.251365
H	3.399264	−2.032965	1.239457
C	−1.600540	0.999156	0.989444
C	−1.880274	0.066119	−0.092053
C	−0.863182	0.017586	−0.955502
C	0.200138	0.957330	−0.477176

Table S2. *Cont.*

IM	x	y	z
C	−0.391862	1.525704	0.778653
H	−2.277061	1.205934	1.800920
H	0.356119	1.755007	−1.207653
H	0.122263	2.240138	1.398402
H	−0.784613	−0.589990	−1.839050
Cl	−3.345295	−0.817883	−0.185608
IM67			
0	2		
C	3.064812	−0.734531	−0.196122
C	2.203906	−0.115235	−1.023000
C	0.996691	0.209533	−0.266320
C	1.234776	−0.287703	1.090681
C	2.458388	−0.840975	1.120246
H	4.045394	−1.096893	−0.455794
H	2.349782	0.117191	−2.064754
H	0.532724	−0.209460	1.901543
H	2.925967	−1.295735	1.977467
C	−0.641287	2.035612	0.458621
C	−1.650318	1.138102	0.740071
C	−1.534242	0.047754	−0.106993
C	−0.370144	0.188713	−0.978311
C	0.215630	1.498817	−0.602953
H	−0.463636	2.967134	0.967213
H	−2.390632	1.235949	1.515795
H	−0.378038	−0.203973	−1.981682
H	0.693868	2.148017	−1.318177
Cl	−2.540276	−1.323691	−0.115125
IM68			
0	2		
C	3.371764	0.438610	−0.000429
C	2.642672	−0.691781	−0.000282
C	1.229445	−0.323659	0.000120
C	1.201325	1.141486	0.000088
C	2.471164	1.579463	−0.000011
H	4.447174	0.500554	−0.000726
H	3.007790	−1.705305	−0.000257
H	0.307155	1.738997	−0.000064
H	2.784530	2.610020	0.000119
C	−1.039180	−0.573043	−1.143722
C	−1.721370	−0.204313	0.000025
C	−1.039062	−0.571921	1.143960
C	0.209266	−1.217923	0.741662
C	0.209172	−1.218431	−0.740931
H	−1.340625	−0.366428	−2.155031

Table S2. *Cont.*

IM	x	y	z
H	0.640355	−2.012541	1.328534
H	0.640823	−2.014274	−1.325759
H	−1.341227	−0.365413	2.155082
Cl	−3.197479	0.674321	−0.000281
IM69			
0	2		
C	2.972549	−0.728163	−0.728358
C	1.811324	−0.237127	−1.169559
C	0.932579	0.141829	−0.000602
C	1.809680	−0.236592	1.169653
C	2.971546	−0.727804	0.730303
H	3.789230	−1.073386	−1.341125
H	1.496495	−0.103357	−2.190377
H	1.493532	−0.102529	2.190027
H	3.787367	−1.072669	1.344410
C	−0.578128	2.136221	−0.000016
C	−1.720305	1.314187	0.001022
C	−1.545267	−0.082348	0.000020
C	−0.326967	−0.667549	−0.001216
C	0.672532	1.615719	−0.001143
H	−0.708582	3.207913	−0.000213
H	−2.712352	1.732328	0.002375
H	−0.226258	−1.741228	−0.002206
H	1.542368	2.254920	−0.002449
Cl	−2.968180	−1.074837	−0.000062
IM70			
0	2		
C	3.303482	0.000263	0.728767
C	2.041883	0.000875	1.169037
C	1.086282	0.000274	0.000230
C	2.041790	−0.000557	−1.168792
C	3.303463	−0.000782	−0.728725
H	4.189905	0.000291	1.341995
H	1.699825	0.001525	2.189968
H	1.699602	−0.001068	−2.189681
H	4.189796	−0.001680	−1.342067
C	−1.100650	−1.224345	0.000372
C	−1.796086	0.000002	−0.000071
C	−1.100809	1.224588	−0.000692
C	0.251508	1.244848	−0.000745
C	0.251614	−1.244495	0.000509
H	−1.660914	−2.146342	−0.000105
H	0.785935	2.182692	−0.001429
H	0.786153	−2.182217	0.000153

Table S2. *Cont.*

IM	x	y	z
H	−1.661002	2.146602	−0.001176
Cl	−3.513186	−0.000225	0.000177
IM71			
0	2		
C	3.123907	−0.848114	−0.101189
C	1.953623	−1.193405	−0.752360
C	0.839268	−0.399304	−0.214129
C	1.453557	0.470179	0.842730
C	2.870674	0.102878	0.871733
H	4.098327	−1.243308	−0.337002
H	1.853662	−1.894068	−1.564302
H	0.904024	0.721511	1.737057
H	3.597018	0.550784	1.528616
C	−0.245905	1.900606	−0.445158
C	−1.433801	1.353390	−0.139316
C	−1.560618	−0.072972	0.011857
C	−0.523916	−0.908407	−0.117841
C	0.987491	1.142680	−0.435002
H	−0.199365	2.937019	−0.745936
H	−2.331885	1.949340	−0.125214
H	−0.676222	−1.975506	−0.152804
H	1.779537	1.461877	−1.097400
Cl	−3.165340	−0.693696	0.213473
IM72			
0	2		
C	−2.939582	−1.183636	−0.116926
C	−1.635553	−1.569880	0.040813
C	−0.607771	−0.625616	0.160191
C	−2.348444	1.154830	−0.043842
C	−3.273566	0.201919	−0.239983
H	−3.712026	−1.926815	−0.236114
H	−1.374640	−2.618156	0.010439
H	−2.593244	2.201622	−0.150560
H	−4.279416	0.474007	−0.522255
C	0.087180	1.764198	−0.001929
C	1.363161	1.373821	−0.137073
C	1.705433	−0.005257	0.010924
C	0.742992	−0.975515	0.105849
C	−0.974355	0.805103	0.420174
H	−0.186892	2.800490	−0.134033
H	2.141190	2.077082	−0.388209
H	1.024507	−2.017214	0.072290
H	−1.014199	0.890850	1.529853
Cl	3.369279	−0.442451	−0.080623

Table S2. *Cont.*

IM	x	y	z
IM73			
0	2		
C	2.879682	−0.921866	−0.287199
C	2.314673	0.183934	−0.894218
C	1.084691	0.565708	−0.182521
C	0.958201	−0.435530	0.934426
C	2.135160	−1.299412	0.815547
H	3.760293	−1.433648	−0.639143
H	2.671111	0.680570	−1.781076
H	0.546707	−0.148276	1.889830
H	2.343332	−2.126861	1.472459
C	−0.650568	2.240711	0.227727
C	−1.675073	1.228205	0.267057
C	−1.378036	−0.045643	−0.027475
C	−0.025546	−0.530344	−0.213221
C	0.624354	1.944283	−0.058439
H	−0.949410	3.268969	0.359156
H	−2.700633	1.516221	0.431975
H	0.114228	−1.359275	−0.891693
H	1.351950	2.727414	−0.209090
Cl	−2.631930	−1.217964	−0.242502
IM74			
0	2		
C	−2.893937	−0.648087	−0.062120
C	−2.411366	0.624407	0.066224
C	−1.034050	0.882899	0.133765
C	−0.665223	−1.580044	−0.066642
C	−1.988077	−1.743468	−0.220367
H	−3.955492	−0.820135	−0.143074
H	−3.090058	1.465211	0.044174
H	0.017755	−2.403333	−0.198136
H	−2.379764	−2.710127	−0.498816
C	0.836149	2.384413	−0.128936
C	1.739757	1.285430	−0.178402
C	1.300903	0.031562	0.020805
C	−0.111616	−0.274207	0.407670
C	−0.511735	2.170002	0.004793
H	1.220318	3.382770	−0.262391
H	2.783836	1.459325	−0.384751
H	−0.080855	−0.369516	1.516663
H	−1.199229	3.001570	−0.052940
Cl	2.418745	−1.282542	0.006973
IM75			
0	2		

Table S2. *Cont.*

IM	x	y	z
C	−3.374279	−0.063219	−0.158772
C	−2.461627	0.864424	−0.627003
C	−1.127095	0.551197	−0.098100
C	−1.319777	−0.671905	0.751787
C	−2.752894	−0.965255	0.686906
H	−4.415598	−0.094891	−0.434499
H	−2.661433	1.669676	−1.313955
H	−0.740927	−0.810679	1.652261
H	−3.222327	−1.792798	1.191325
C	0.874781	−0.985589	−0.549591
C	1.666458	−0.032921	−0.034049
C	1.175776	1.270315	0.330223
C	−0.126342	1.562839	0.210268
C	−0.565933	−0.841021	−0.551713
H	1.308266	−1.861707	−1.006399
H	−0.466914	2.579225	0.340097
H	−1.113569	−1.346349	−1.334477
H	1.892329	2.028172	0.601737
Cl	3.381516	−0.264932	0.032010
IM76			
0	2		
C	3.280730	0.224546	−0.221433
C	2.314602	1.185352	−0.074448
C	0.974269	0.841182	0.131424
C	1.670522	−1.555007	0.033141
C	2.922942	−1.159188	−0.246707
H	4.303464	0.510901	−0.408828
H	2.569722	2.229851	−0.184093
H	1.393541	−2.598497	−0.000685
H	3.670394	−1.886964	−0.524214
C	−0.763249	−0.958427	0.193692
C	−1.697288	−0.008989	0.049849
C	−1.379468	1.384022	0.058283
C	−0.067020	1.772768	0.063745
C	0.645770	−0.574905	0.497644
H	−1.023492	−2.005043	0.166132
H	0.177703	2.819510	−0.046275
H	0.717082	−0.599856	1.609602
H	−2.176391	2.104154	−0.023368
Cl	−3.355464	−0.440129	−0.205848
IM77			
0	2		
C	3.005538	0.949120	0.552353
C	1.730463	1.191985	1.208012

Table S2. *Cont.*

IM	x	y	z
C	0.805775	0.343966	0.701323
C	1.507580	−0.446033	−0.292446
C	2.853288	−0.057634	−0.365302
H	3.916398	1.483973	0.761058
H	1.556451	1.933736	1.968900
H	3.593676	−0.477574	−1.020000
C	−2.187077	−1.480283	0.976141
C	−2.526342	−0.519059	−0.063463
C	−1.619375	0.460343	−0.031769
C	−0.631900	0.239407	1.074506
C	−1.089093	−1.078351	1.624697
H	−2.749395	−2.377503	1.175245
H	−0.589350	−1.577510	2.436968
H	−3.361159	−0.589042	−0.739011
H	−0.823488	1.003511	1.834571
Cl	−1.545300	1.834590	−1.042603
Cl	0.801989	−1.659317	−1.235164
IM78			
0	2		
C	−1.139129	2.408790	−0.805466
C	0.081171	1.930940	−0.520758
C	−0.066107	0.575021	0.026065
C	−1.517396	0.348137	0.048407
C	−2.145044	1.429345	−0.439253
H	−1.357416	3.369468	−1.240215
H	1.029046	2.408953	−0.688792
H	−3.210589	1.555437	−0.517541
C	2.887557	0.626632	0.367380
C	2.292259	−0.044782	−0.680920
C	0.966744	−0.520884	−0.260596
C	0.806519	−0.060473	1.136643
C	2.051001	0.634708	1.471301
H	3.850712	1.107286	0.320177
H	2.245111	1.105667	2.419328
H	2.685418	−0.194773	−1.671040
H	0.241945	−0.627159	1.859959
Cl	0.455897	−2.070704	−0.876366
Cl	−2.267054	−1.028678	0.727031
IM79			
0	2		
C	1.323657	2.501920	0.298769
C	0.102976	2.010079	0.549477
C	0.108581	0.559690	0.307191
C	1.492151	0.281150	−0.134386

Table S2. *Cont.*

IM	x	y	z
C	2.193289	1.424149	−0.130669
H	1.627508	3.530024	0.396123
H	−0.767680	2.550776	0.875949
H	3.229201	1.518497	−0.405998
C	−0.730130	−2.359513	0.254236
C	−1.105598	−1.464732	−0.725508
C	−1.181298	−0.117363	−0.146280
C	−0.787726	−0.271575	1.272944
C	−0.520374	−1.699389	1.451902
H	−0.553001	−3.409034	0.089298
H	−0.175406	−2.138647	2.371762
H	−1.286325	−1.671074	−1.765537
H	−1.228224	0.319683	2.058841
Cl	−2.512535	0.887298	−0.685149
Cl	2.146698	−1.233575	−0.585821
IM80			
0	2		
C	−2.016422	0.313947	1.717855
C	−0.690076	0.414686	1.825338
C	−0.024925	−0.167094	0.598737
C	−1.238769	−0.551486	−0.220335
C	−2.367389	−0.288730	0.438479
H	−2.737131	0.628523	2.454412
H	−0.119882	0.814355	2.645732
H	−3.365895	−0.482466	0.086607
C	2.799239	−0.574653	0.059111
C	2.170911	0.610568	−0.350415
C	0.852513	0.816504	−0.109077
C	0.736326	−1.402208	0.984555
C	2.052051	−1.567649	0.724736
H	3.847041	−0.722209	−0.143668
H	2.539133	−2.480874	1.031187
H	2.733030	1.372492	−0.867181
H	0.161908	−2.163751	1.489515
Cl	0.090531	2.268188	−0.627352
Cl	−1.072822	−1.244033	−1.767384
IM81			
0	2		
C	−2.046997	−1.587716	0.697350
C	−0.731365	−1.421681	0.957182
C	−0.857425	0.813647	−0.100814
C	−2.175701	0.607214	−0.342469
C	−2.799217	−0.586148	0.050232
H	−2.529930	−2.508307	0.987905

Table S2. *Cont.*

IM	x	y	z
H	−0.152168	−2.189493	1.446896
H	−2.741278	1.374567	−0.847259
H	−3.846958	−0.734078	−0.152632
Cl	−0.100347	2.274542	−0.600118
C	2.006570	0.289690	1.734718
C	2.367279	−0.289158	0.447040
C	1.243624	−0.541204	−0.224344
C	0.023595	−0.175716	0.593663
C	0.679372	0.387243	1.834513
H	2.721605	0.591226	2.482181
H	3.368440	−0.475044	0.098503
H	0.102750	0.769908	2.658514
Cl	1.089707	−1.204295	−1.785560
IM82			
0	2		
C	0.379360	1.900855	−1.325965
C	0.900636	0.553655	−1.190824
C	−1.319100	0.035774	−0.047386
C	−1.637029	1.334642	−0.097269
C	−0.771472	2.279168	−0.751382
H	0.935855	2.590560	−1.941902
H	1.503211	0.172030	−2.003501
H	−2.587731	1.665090	0.287938
H	−1.112280	3.298396	−0.845182
Cl	−2.444036	−1.120827	0.565926
C	1.495731	−2.248836	−0.516335
C	2.145446	−1.202766	0.108750
C	1.261043	−0.033725	0.152387
C	−0.029521	−0.488348	−0.479526
C	0.205275	−1.889780	−0.857717
H	1.941950	−3.205930	−0.729170
H	3.154076	−1.190050	0.483317
H	−0.526075	−2.504049	−1.353488
Cl	1.321023	0.987304	1.559579
IM83			
0	2		
C	−2.778905	0.904394	−0.132687
C	−1.909721	2.006664	0.181127
C	−0.592853	1.822250	0.338137
C	−0.926532	−0.695775	−0.066789
C	−2.308545	−0.335922	−0.336393
H	−3.824219	1.104360	−0.311119
H	−2.320121	3.004134	0.191032
H	−2.945481	−1.113495	−0.728772

Table S2. *Cont.*

IM	x	y	z
C	1.879487	−0.780493	1.002390
C	0.814984	−1.302893	1.700817
C	−0.422398	−0.595831	1.350930
C	−0.008015	0.489482	0.380154
C	1.436860	0.274418	0.226538
H	2.888999	−1.153607	1.010639
H	0.848722	−2.144900	2.369912
H	0.083644	2.659792	0.415815
H	−1.186897	−0.410603	2.090906
Cl	−0.310429	−1.960048	−1.114488
Cl	2.389792	1.215139	−0.821027
IM84			
0	2		
C	−2.842477	−0.181640	0.635654
C	−2.189376	−1.319783	0.912946
C	−0.806921	−1.525287	0.520480
C	−0.861693	0.922444	−0.206956
C	−2.172074	0.957650	0.061408
H	−3.877674	−0.069165	0.917946
H	−2.672379	−2.100700	1.481037
H	−2.722317	1.870054	−0.101348
C	1.906428	−1.464640	−0.595893
C	0.892365	−2.129827	−1.247784
C	−0.395135	−1.499829	−0.940193
C	−0.071057	−0.303121	−0.090564
C	1.390338	−0.365854	0.074346
H	2.941597	−1.759384	−0.566069
H	1.002814	−3.009460	−1.858195
H	−1.194081	−1.442403	−1.662971
H	−0.203302	−2.169027	1.144327
Cl	2.261662	0.627060	1.145727
Cl	−0.048547	2.322317	−0.798397
IM85			
0	2		
C	1.348486	2.607049	−0.294616
C	−0.053644	2.437521	−0.126708
C	−0.616505	1.197594	0.033708
C	1.638300	0.271156	0.409669
C	2.171218	1.574394	−0.081480
H	1.727529	3.570224	−0.598797
H	−0.708671	3.290200	−0.194752
H	1.662240	0.356527	1.519979
H	3.240585	1.667547	−0.195642
Cl	−2.347318	1.205711	0.115622

Table S2. *Cont.*

IM	x	y	z
C	0.706379	−2.348685	−0.058689
C	2.101692	−2.095406	−0.091406
C	2.560185	−0.849864	0.086209
C	0.169585	0.022602	0.098840
C	−0.213480	−1.326429	0.005944
H	0.341417	−3.357326	−0.157283
H	2.777837	−2.914836	−0.279512
H	3.616448	−0.628639	0.056484
Cl	−1.860370	−1.848257	−0.117934
IM86			
0	2		
C	−2.353726	0.237509	1.158776
C	−1.216083	−0.245338	1.674083
C	0.075947	0.745532	−0.310060
C	−1.210138	1.305417	−0.696865
C	−2.346977	1.015007	−0.049604
H	−3.281772	0.116699	1.695027
H	−1.205373	−0.717467	2.644598
H	−1.204051	1.990253	−1.529959
H	−3.275122	1.437365	−0.402045
Cl	1.414410	1.837052	−0.637846
C	2.261887	−0.940345	0.874744
C	1.712050	−1.202287	−0.362469
C	0.322150	−0.731920	−0.403213
C	0.058675	−0.153754	0.976001
C	1.331510	−0.331513	1.692316
H	3.284572	−1.141701	1.145916
H	2.197100	−1.644946	−1.214507
H	1.487567	0.009413	2.701706
Cl	−0.815274	−1.733492	−1.265859

Table S3. Cartesian coordinates for the transition states involved in PCN formation from 3-CP.

TS	x	y	z
TS1			
0	1		
C	−2.423543	−1.401200	0.380024
C	−3.388639	−0.477355	0.119179
C	−3.249673	0.792369	−0.492328
C	−2.085393	1.418088	−0.785529
C	−0.585792	0.153955	0.145457
C	−1.075345	−1.112125	0.082701
H	0.818004	−0.709138	−2.094597

Table S3. *Cont.*

TS	x	y	z
C	1.417149	−0.368253	−1.262167
C	2.687909	−0.748714	−1.155270
C	0.778571	0.568035	−0.303494
C	3.489395	−0.277449	−0.043701
C	1.627821	0.956233	0.896819
C	3.018696	0.526567	0.918289
H	3.150542	−1.400429	−1.878134
H	−1.023317	0.821234	0.882816
O	−1.487854	2.351692	−1.156227
H	−0.441449	−1.921885	−0.260779
H	−2.724573	−2.398260	0.655341
H	0.646066	1.518593	−0.844035
O	1.148955	1.615768	1.791623
Cl	5.119754	−0.822161	−0.002095
Cl	−5.031011	−0.936811	0.394989
H	−4.112996	1.228038	−0.974463
H	3.623337	0.843794	1.751598
TS2			
0	1		
C	2.580629	−1.251248	0.196141
C	3.398905	−0.061839	0.080713
C	2.912117	1.143207	−0.246028
C	1.491094	1.310646	−0.493960
C	0.606542	0.073086	−0.469220
C	1.272674	−1.182868	−0.039853
C	−1.123871	−0.325571	1.371215
C	−2.509011	−0.167126	1.557000
C	−0.684847	0.379999	0.250637
C	−3.142464	0.220346	0.416849
C	−2.301288	−1.064727	−1.404740
C	−2.321035	0.228798	−0.800663
O	−1.732622	−2.076068	−1.309770
O	1.003468	2.393428	−0.736106
H	0.653187	−2.063574	0.038422
H	−0.531653	−1.049331	1.905955
H	3.059116	−2.174998	0.477058
H	−3.044927	−0.390667	2.465782
H	−0.912052	1.441713	0.224800
H	0.347037	−0.072839	−1.526479
H	3.534088	2.020991	−0.300086
H	−2.332383	1.055190	−1.496552
Cl	5.074357	−0.260794	0.406948
Cl	−4.839996	0.430112	0.303028

Table S3. *Cont.*

TS	x	y	z
TS3			
0	1		
C	2.042267	−1.359000	0.233205
C	2.996145	−0.437478	−0.074157
C	2.864562	0.969368	−0.158548
C	1.704664	1.669221	−0.110425
C	0.177169	0.133874	−0.166785
C	0.714401	−0.944701	0.464435
C	−1.729261	0.409292	1.482122
C	−2.964400	−0.078528	1.346020
C	−1.160843	0.726118	0.138303
C	−3.269800	−0.146898	−0.076964
C	−2.245317	0.300594	−0.803802
O	1.118119	2.676018	−0.214277
H	0.133028	−1.467530	1.214514
H	−1.206961	0.593721	2.406092
H	2.353093	−2.364239	0.464649
H	−3.647773	−0.368474	2.125780
H	−1.056312	1.818231	0.067638
H	0.558465	0.342482	−1.163190
H	3.748375	1.577407	−0.028718
H	−2.185565	0.362854	−1.875858
Cl	4.626363	−0.991819	−0.223365
Cl	−4.768647	−0.734874	−0.667054
TS4			
0	1		
C	1.961433	−1.103626	1.085388
C	2.683700	−0.618323	0.034332
C	1.968758	0.285817	−0.877762
C	2.132309	1.636263	−0.426156
C	0.259335	−0.455229	−0.328957
C	0.600235	−0.788950	0.986041
C	−1.448836	1.329748	0.318153
C	−2.724184	1.092121	0.631527
C	−0.981740	0.321027	−0.683374
C	−3.162208	−0.085926	−0.106049
C	−2.177310	−0.563679	−0.866990
O	1.714877	2.402185	0.338430
H	2.428644	−1.601138	1.920606
H	−3.351221	1.650832	1.304964
H	−2.209976	−1.425108	−1.510187
H	2.008869	0.175005	−1.951556
H	−0.796384	0.840293	−1.631139
H	0.496754	−1.219472	−1.064265

Table S3. *Cont.*

TS	x	y	z
H	−0.826135	2.121367	0.698016
H	−0.039012	−0.604120	1.834013
Cl	−4.747190	−0.722106	0.034275
Cl	4.388395	−0.774989	−0.087499
TS5			
0	1		
C	1.886848	0.799759	−1.133960
C	2.791716	0.272746	−0.135673
C	2.408991	−0.086637	1.097283
C	1.015165	0.030747	1.495815
C	0.070042	0.747743	0.541387
C	0.604699	0.983026	−0.827978
C	−1.634088	−1.050333	0.008096
C	−2.981220	−1.475576	−0.080652
C	−1.293738	0.138451	0.565667
C	−3.966662	−0.555638	−0.252003
C	−2.734095	1.598463	−0.210316
C	−3.847225	0.849477	−0.381237
O	−2.163756	2.614312	−0.118158
H	−4.657411	1.402868	−0.835577
H	−4.967620	−0.934472	−0.398584
H	−3.206975	−2.526109	−0.154689
H	−0.033578	1.749366	0.978195
O	0.613456	−0.384694	2.558724
H	−0.087097	1.367266	−1.563072
H	2.274071	1.015917	−2.115866
Cl	−0.431366	−2.052067	−0.716780
Cl	4.432415	0.090059	−0.620324
H	3.092855	−0.519793	1.808089
H	−1.952290	0.448780	1.369176
TS6			
0	1		
C	1.831996	1.010163	−0.798099
C	2.779140	0.267247	0.002951
C	2.437158	−0.461949	1.074894
C	1.049357	−0.534046	1.494427
C	0.048181	0.363880	0.777754
C	0.543406	1.016681	−0.463955
C	−1.715001	−1.109292	−0.380586
C	−3.116402	−1.226112	−0.417826
C	−1.292098	−0.317356	0.693891
C	−3.743684	−0.268174	0.312218
C	−2.884101	1.998421	0.191531
C	−2.849303	0.742878	0.913468

Table S3. *Cont.*

TS	x	y	z
O	−2.354019	2.575882	−0.665832
H	−4.816533	−0.190547	0.383273
O	0.688421	−1.232730	2.416073
H	−0.174853	1.555763	−1.064219
H	2.193562	1.525915	−1.672081
H	−3.640818	−1.952880	−1.017540
H	−1.559122	−0.745537	1.654836
H	−0.083069	1.185042	1.496584
H	3.155493	−1.039994	1.631804
H	−2.861434	0.930426	1.979279
Cl	4.420228	0.333127	−0.506910
Cl	−0.739071	−1.562977	−1.716495
TS7			
0	1		
C	−2.237182	0.397407	1.286532
C	−2.685625	0.077975	0.038933
C	−1.972533	−0.497040	−1.033777
C	−0.631589	−0.716548	−1.093619
C	0.110944	0.262202	0.692451
C	−0.884453	0.202749	1.619479
C	2.549677	0.359432	0.010887
C	3.329428	−0.641223	−0.400985
C	1.530905	−0.122787	0.992211
C	2.869104	−1.862066	0.244214
C	1.824738	−1.592553	1.032740
H	3.314767	−2.830634	0.089596
O	0.308222	−1.037348	−1.705315
H	−2.960709	0.639497	2.046872
H	4.150808	−0.564257	−1.091754
H	1.796684	0.297110	1.970564
H	−0.031575	0.965524	−0.123630
H	1.259446	−2.287376	1.630112
H	−2.529073	−1.025330	−1.794642
Cl	2.639045	2.003314	−0.451710
H	−0.647235	−0.067327	2.643179
Cl	−4.382538	0.229997	−0.257831
TS8			
0	1		
C	−2.167185	−0.320479	1.460133
C	−2.186646	0.274675	0.231127
C	−0.899465	0.321695	−0.488887
C	−0.844308	−0.815709	−1.343445
C	0.064179	0.119390	1.154408
C	−0.870575	−0.620288	1.894500

Table S3. *Cont.*

TS	x	y	z
C	2.227311	0.467720	−0.069867
C	2.836258	−0.380122	−0.901787
C	1.511426	−0.270342	1.015802
C	2.565580	−1.734483	−0.434755
C	1.795086	−1.691911	0.655973
H	2.930844	−2.623661	−0.920601
O	−0.690676	−1.954176	−1.442242
H	−0.631134	−1.479120	2.499989
H	1.412640	−2.531616	1.209584
H	−0.057289	1.197102	1.231542
H	2.000625	−0.034433	1.968628
H	−0.486200	1.222613	−0.917007
H	3.429575	−0.114648	−1.759677
H	−3.075684	−0.585412	1.978165
Cl	2.215142	2.176627	−0.154887
Cl	−3.637962	0.675238	−0.597578
TS9			
0	1		
C	3.153808	0.944521	−0.788841
C	3.800299	−0.311253	−1.102307
C	3.355320	−1.493632	−0.657145
C	2.166726	−1.571433	0.179268
C	1.396550	−0.283037	0.459115
C	2.038344	0.953310	−0.061505
C	−0.561943	0.080545	−1.122130
C	−1.936095	−0.050638	−1.410208
C	−0.013049	−0.485091	−0.015852
C	−2.834511	−0.144183	−0.391457
C	−1.389873	−0.078927	1.613460
C	−2.598614	−0.075708	1.002713
O	−0.724577	−0.120540	2.572257
H	−3.412078	0.225107	1.647121
H	−2.305105	0.095568	−2.411590
H	1.347952	−0.200801	1.551794
O	1.755943	−2.612774	0.637535
H	3.592794	1.866992	−1.132089
H	3.852625	−2.422347	−0.885792
H	−0.446478	−1.415618	0.339640
H	4.688848	−0.271178	−1.715023
H	0.039220	0.710426	−1.767873
Cl	1.272218	2.434852	0.364106
Cl	−4.511657	−0.177704	−0.807353
TS10			
0	1		

Table S3. *Cont.*

TS	x	y	z
C	−2.486056	0.848898	1.054145
C	−2.855108	−0.495958	1.439878
C	−2.554099	−1.568624	0.699117
C	−1.828724	−1.427284	−0.554453
C	−1.321465	−0.046940	−0.952510
C	−1.789958	1.053688	−0.062259
C	0.971473	−1.062951	−1.579397
C	2.309434	−0.863291	−1.209120
C	0.187976	0.003436	−1.127425
C	2.448338	0.035530	−0.192858
C	0.921418	−0.331711	1.574728
C	1.203536	0.497237	0.441107
O	0.476259	−1.352834	1.890424
O	−1.634575	−2.362666	−1.296950
H	0.576527	−1.994034	−1.946968
H	−2.800517	1.680591	1.663219
H	3.150375	−1.405502	−1.611827
H	0.502947	0.978193	−1.487913
H	−1.739566	0.131537	−1.948153
H	−2.840857	−2.568116	0.982189
H	0.980882	1.542415	0.596822
H	−3.405458	−0.615640	2.360984
Cl	3.957759	0.409683	0.533392
Cl	−1.392959	2.656340	−0.565266
TS11			
0	1		
C	−2.548525	1.579552	−0.408823
C	−3.626401	0.754139	−0.318403
C	−3.648732	−0.631156	−0.042115
C	−2.582254	−1.463922	0.048553
C	−0.923332	−0.209795	−0.592557
C	−1.246595	1.059004	−0.221606
C	0.783885	−1.025415	1.124366
C	2.080779	−0.725087	1.215167
C	0.347876	−0.944368	−0.304685
C	2.553979	−0.363214	−0.111964
C	1.572431	−0.450371	−1.011826
O	−2.115403	−2.531049	0.145552
H	−2.693015	2.645713	−0.461750
H	2.699383	−0.738929	2.095799
H	0.183363	−1.981326	−0.620974
H	−1.482999	−0.550152	−1.459610
H	1.631010	−0.238168	−2.064277
H	−4.562095	−1.076094	0.327661

Table S3. *Cont.*

TS	x	y	z
H	−4.600895	1.217480	−0.371629
H	0.128931	−1.340952	1.918735
Cl	−0.114269	2.060069	0.601415
Cl	4.175497	0.106674	−0.412298
TS12			
0	1		
C	−2.543086	1.594993	−0.294142
C	−3.320806	0.565183	−0.722999
C	−2.612505	−0.721540	−0.887622
C	−2.935373	−1.646533	0.189833
C	−0.902757	0.070099	−0.903310
C	−1.187123	1.244410	−0.187767
C	0.603308	−1.221292	0.752825
C	1.898715	−1.010971	0.997588
C	0.288904	−0.814918	−0.652735
C	2.488617	−0.392705	−0.179009
C	1.580596	−0.241413	−1.144812
O	−2.593479	−1.993329	1.241727
H	−2.939593	2.552815	0.003523
H	2.440530	−1.235159	1.900112
H	1.730460	0.188136	−2.119269
H	−2.636677	−1.251296	−1.830954
H	0.112252	−1.742347	−1.212429
H	−1.087515	0.198016	−1.966912
H	−0.118219	−1.653152	1.424084
H	−4.388923	0.645366	−0.845012
Cl	4.140102	0.059537	−0.252334
Cl	−0.170421	1.922367	1.011507
TS13			
0	1		
C	2.583505	0.770300	−0.894635
C	3.471692	0.001771	−0.056067
C	3.076689	−0.605001	1.072061
C	1.694729	−0.526072	1.513409
C	0.733845	0.346776	0.701942
C	1.298288	0.893130	−0.566643
C	−1.051491	−1.179997	−0.317547
C	−2.400281	−1.598125	−0.366485
C	−0.606978	−0.308948	0.624706
C	−3.394641	−0.764507	0.043430
C	−2.181840	1.214401	0.929842
C	−3.298944	0.547532	0.556180
O	−1.621493	2.094992	1.450778
H	−4.170772	1.186269	0.521139

Table S3. *Cont.*

TS	x	y	z
H	−4.407299	−1.106200	−0.114703
H	−2.651058	−2.518373	−0.866902
H	0.591552	1.232783	1.336132
O	1.286434	−1.095087	2.499893
H	2.965511	1.233283	−1.789237
Cl	−0.014037	−1.758677	−1.570770
H	3.747098	−1.193803	1.677031
H	−1.096008	−0.425751	1.585809
H	4.500707	−0.084399	−0.372970
Cl	0.228936	1.813347	−1.548220
TS14			
0	1		
C	−2.332198	−0.672595	−1.198537
C	−3.366063	−0.372059	−0.238817
C	−3.117592	−0.124142	1.054979
C	−1.759759	−0.150108	1.564994
C	−0.630757	−0.515929	0.598597
C	−1.055402	−0.699550	−0.818367
C	0.872130	1.526949	0.097772
C	2.238879	1.832499	0.146911
C	0.561082	0.381032	0.856565
C	2.997636	0.804949	0.610614
C	2.619243	−1.711905	0.465850
C	2.244774	−0.390198	1.005493
O	2.174548	−2.696820	0.054008
H	4.071323	0.851707	0.695164
O	−1.490225	0.073027	2.724964
H	−4.383741	−0.342521	−0.599490
H	−2.599119	−0.858255	−2.225591
H	2.655590	2.752089	−0.231183
H	0.629220	0.561387	1.925065
H	−0.324280	−1.514042	0.936411
H	2.253174	−0.635899	2.067711
H	−3.897730	0.115883	1.758884
Cl	0.181091	−1.052827	−1.957587
Cl	−0.152318	2.264964	−1.068006
TS15			
0	1		
C	2.224509	1.591575	−0.023839
C	3.184498	0.625526	−0.022937
C	3.032327	−0.769697	0.122861
C	1.902154	−1.444941	0.451661
C	0.482476	0.165745	0.874732
C	0.865607	1.239356	0.131187

Table S3. *Cont.*

TS	x	y	z
C	−1.630121	−0.657078	−0.314633
C	−2.888306	−0.225132	−0.203375
C	−0.879611	−0.435580	0.960866
C	−3.041040	0.384458	1.107715
C	−1.890729	0.302642	1.785444
H	−3.954777	0.831037	1.463412
O	1.338555	−2.415211	0.773480
H	4.191691	0.943493	−0.250587
H	2.482950	2.598653	−0.305448
H	−3.653395	−0.302853	−0.956028
H	−0.750589	−1.429332	1.408112
H	1.134208	−0.018622	1.723748
H	−1.691596	0.662006	2.780317
H	3.821651	−1.420547	−0.226873
Cl	−0.288924	2.166373	−0.747905
Cl	−0.914556	−1.413631	−1.666365
TS16			
0	1		
C	2.510708	0.792459	−0.958852
C	1.770279	1.923190	−0.775609
C	0.385873	1.685443	−0.290860
C	0.339066	2.046061	1.100445
C	0.394627	−0.111194	−0.753819
C	1.777552	−0.383729	−0.758454
C	−2.009914	−0.405552	−0.161860
C	−2.565990	−0.317085	1.048228
C	−0.631412	−0.980799	−0.078240
C	−1.602972	−0.822419	2.018018
C	−0.481032	−1.194642	1.394517
H	−1.785905	−0.874353	3.078284
O	0.571990	1.793501	2.199615
H	0.408883	−1.599398	1.842388
H	0.049035	0.216499	−1.731091
H	−0.620281	−1.953430	−0.584196
H	−0.493174	2.030985	−0.816671
H	−3.552409	0.052334	1.269680
H	3.569541	0.812493	−1.164927
Cl	−2.700609	0.072440	−1.653264
H	2.178244	2.916652	−0.865242
Cl	2.485870	−1.798333	−0.071808
TS17			
0	2		
C	−2.505911	0.497719	1.158770
C	−1.558884	1.442013	1.209266

Table S3. *Cont.*

TS	x	y	z
C	−0.723183	1.355365	−0.011269
C	−1.256419	0.194214	−0.749773
C	−2.305804	−0.271191	−0.061219
H	−3.293094	0.313286	1.869433
H	−1.425301	2.185229	1.977279
C	2.453798	0.539237	−1.156412
C	2.338360	−0.217884	0.082925
C	1.376711	0.285059	0.858815
C	0.783491	1.461776	0.154644
C	1.541750	1.513971	−1.132383
H	3.166132	0.322324	−1.933939
H	1.363292	2.250989	−1.897721
H	0.995209	2.372689	0.725164
H	−1.028897	2.328808	−0.652653
H	1.055903	−0.077337	1.819219
Cl	3.335556	−1.567655	0.428465
H	−0.861853	−0.181538	−1.676692
Cl	−3.314100	−1.584885	−0.494456
H	−1.199603	3.277051	−1.228420
TS18			
0	2		
C	−2.348684	−0.315632	1.326941
C	−1.552897	0.730536	1.569286
C	−0.754072	1.029909	0.351994
C	−1.156623	−0.015975	−0.619850
C	−2.092079	−0.770181	−0.035912
H	−3.064651	−0.768547	1.991242
H	−1.497888	1.307597	2.476816
C	2.439133	0.801001	−0.972054
C	2.434289	−0.225658	0.061546
C	1.460986	−0.005505	0.947663
C	0.744090	1.240331	0.540442
C	1.447909	1.656564	−0.709984
H	3.138407	0.832112	−1.790009
H	1.165170	2.526700	−1.278542
H	0.882375	2.015130	1.301051
H	−1.136909	2.045493	−0.038864
H	1.210221	−0.605056	1.804626
Cl	3.557053	−1.519971	0.073898
H	−0.745379	−0.125788	−1.607406
Cl	−2.910310	−2.107132	−0.723207
O	−1.541104	3.334214	−0.684991
H	−2.349468	3.007069	−1.101162

Table S3. *Cont.*

TS	x	y	z
TS19			
0	2		
C	2.335477	1.245757	−0.778478
C	1.238177	1.835301	−0.297654
C	0.721866	1.059019	0.870709
C	1.672488	−0.089174	0.973296
C	2.591999	0.052140	0.015381
H	2.945208	1.564798	−1.606257
H	0.764321	2.728511	−0.669406
C	−1.913483	−1.237898	1.427765
C	−1.686121	−1.359252	−0.003885
C	−0.976986	−0.322180	−0.454165
C	−0.719216	0.589024	0.705455
C	−1.333523	−0.105340	1.855123
H	−2.452598	−1.959585	2.017889
H	−1.348055	1.514634	0.540281
H	−1.323506	0.274603	2.862641
Cl	−2.333986	2.794447	−0.615008
H	0.781106	1.677786	1.771219
H	1.598252	−0.882937	1.695275
H	−0.650247	−0.131504	−1.460708
Cl	3.915595	−0.987934	−0.292006
Cl	−2.281523	−2.676553	−0.918411
TS20			
0	2		
C	−1.747527	−1.396254	−0.045869
C	−0.465033	−1.000841	−0.189342
C	−0.452195	0.447404	−0.280164
C	−1.798769	0.903742	−0.169489
C	−2.568958	−0.210798	−0.037368
H	−2.117833	−2.400704	0.054803
H	0.407851	−1.627485	−0.221376
C	1.910776	0.599046	−1.089289
C	2.540959	0.140717	0.022212
C	1.870550	0.545313	1.201968
C	0.771191	1.311416	0.819653
C	0.712426	1.333675	−0.653655
H	2.178097	0.396154	−2.110535
H	0.422506	2.233692	−1.178083
H	0.169311	1.924620	1.463961
H	2.150897	0.275796	2.204200
Cl	3.933557	−0.858913	0.029675
H	−2.136090	1.923649	−0.213163
Cl	−4.269749	−0.244984	0.119046

Table S3. *Cont.*

TS	x	y	z
TS21			
0	2		
C	2.720299	1.056419	0.063194
C	1.642709	1.869060	−0.055945
C	0.482828	1.040565	−0.250925
C	0.912817	−0.335222	−0.227873
C	2.250088	−0.306826	−0.043805
H	3.746811	1.341287	0.211949
H	1.637240	2.945312	−0.031005
C	−1.807175	0.481064	1.202470
C	−2.320259	−0.081852	0.007033
C	−1.819774	0.542110	−1.088391
C	−0.876082	1.573938	−0.626598
C	−0.953825	1.520465	0.846623
H	−2.021806	0.135169	2.197486
H	−0.550177	2.264869	1.507261
H	−0.846475	2.526627	−1.138296
H	−2.000713	0.282210	−2.115735
Cl	−3.386048	−1.423420	−0.016284
H	0.285589	−1.200116	−0.337560
Cl	3.289564	−1.662090	0.060472
TS22			
0	2		
C	1.723484	−0.286434	1.345553
C	0.401143	−0.088299	1.155085
C	0.188396	0.167166	−0.254880
C	1.456322	0.126663	−0.904895
C	2.370530	−0.154527	0.063987
H	2.229489	−0.498869	2.270139
H	−0.373248	−0.104761	1.900510
C	−2.621227	0.968230	0.647197
C	−2.286387	−0.085957	−0.141067
C	−1.126987	0.252359	−0.982981
C	−0.873144	1.668984	−0.656555
C	−1.760072	2.051373	0.347271
H	−3.390468	0.963316	1.400197
H	−1.769422	3.008922	0.837768
H	−0.220879	2.311941	−1.217085
H	−1.099619	−0.105902	−2.002500
Cl	−2.986281	−1.636654	−0.126051
H	1.643624	0.257969	−1.955285
Cl	4.053821	−0.336873	−0.164892

Table S3. *Cont.*

TS	x	y	z
TS23			
0	2		
C	2.327766	0.780981	−1.095562
C	1.182311	1.499941	−1.183793
C	0.302836	1.064257	−0.131330
C	0.970153	0.018990	0.598072
C	2.179347	−0.133555	0.013001
H	3.200677	0.853623	−1.719683
H	0.957886	2.276436	−1.894972
C	−2.229723	0.153219	1.335902
C	−2.154538	−0.222658	−0.033289
C	−1.449359	0.731186	−0.752791
C	−0.992285	1.749650	0.213031
C	−1.575613	1.326858	1.501561
H	−2.701652	−0.436412	2.103177
H	−1.423506	1.850093	2.429494
H	−1.019423	2.798049	−0.053474
H	−1.384652	0.804914	−1.821637
Cl	−2.785501	−1.669155	−0.673554
H	0.582458	−0.521828	1.440699
Cl	3.398610	−1.238967	0.481059
TS24			
C	−1.774982	1.401432	−0.051703
C	−0.481643	1.082488	0.008124
C	−0.350288	−0.402220	0.085363
C	−1.748755	−0.895770	0.074175
C	−2.550606	0.166445	−0.005582
H	−2.208372	2.383933	−0.126465
H	0.361792	1.750342	−0.006785
C	1.886082	−0.398237	1.205578
C	2.505935	−0.124311	0.043651
C	1.861005	−0.678663	−1.113523
C	0.558891	−1.072867	−0.941453
C	0.585455	−1.020755	1.034858
H	2.301011	−0.191645	2.178848
H	0.285580	−1.891288	1.598855
H	0.098811	−1.798168	−1.593785
H	2.429440	−0.950154	−1.988913
Cl	4.016350	0.685953	−0.085108
H	−2.035611	−1.931420	0.124167
Cl	−4.262185	0.154233	−0.045648
TS25			
0	2		
C	2.666374	1.089197	−0.011517

Table S3. *Cont.*

TS	x	y	z
C	1.572330	1.851285	0.018582
C	0.369351	0.981347	0.059713
C	0.915321	−0.404763	0.057179
C	2.243213	−0.307959	0.009393
H	3.692761	1.411444	−0.043496
H	1.527213	2.927671	0.013996
C	−1.789474	0.409770	1.187670
C	−2.297349	−0.081005	0.042876
C	−1.818976	0.574179	−1.141762
C	−0.679222	1.322950	−0.994856
C	−0.711395	1.359986	0.981421
H	−2.141142	0.144460	2.171510
H	−0.671964	2.305919	1.500827
H	−0.433546	2.112911	−1.687044
H	−2.430000	0.632345	−2.028470
Cl	−3.524435	−1.281105	−0.040928
H	0.317793	−1.297537	0.076830
Cl	3.366662	−1.601667	−0.032975
TS26			
0	2		
C	−1.691716	1.989952	−0.264723
C	−0.566255	1.397618	−0.956278
C	−0.980547	−0.516819	−0.531670
C	−2.191716	−0.238043	0.048118
C	−2.504930	1.124324	0.374563
H	−1.849000	3.057723	−0.278974
H	−0.267943	1.708336	−1.946195
H	−3.358257	1.396907	0.971223
C	1.876528	0.271194	1.355461
C	2.416668	−0.131779	0.061154
C	1.476364	−0.082683	−0.882239
C	0.216868	0.373220	−0.241210
C	0.589268	0.576145	1.191199
H	2.449608	0.298284	2.266296
H	−0.108241	0.905921	1.940905
H	−0.824376	−1.409779	−1.114524
Cl	−3.432945	−1.421668	0.099197
H	1.580748	−0.322532	−1.925754
Cl	4.052609	−0.590899	−0.148563
TS27			
0	2		
C	−1.428968	1.298435	1.563077
C	−0.657707	1.760460	0.428363
C	−1.128163	0.271899	−0.828956

Table S3. *Cont.*

TS	x	y	z
C	−2.051981	−0.290602	0.014617
C	−2.087376	0.134980	1.384900
H	−1.460500	1.868783	2.478882
H	−0.655913	2.792888	0.112452
H	−2.668434	−0.372102	2.135582
C	2.313594	0.731502	−1.159527
C	2.238991	−0.189775	−0.029620
C	1.022615	−0.183584	0.512876
C	0.190184	0.776321	−0.268537
C	1.117379	1.302089	−1.305258
H	3.198500	0.896548	−1.749472
H	0.827578	2.029661	−2.045061
H	−1.267938	0.275609	−1.897645
Cl	−3.336005	−1.246146	−0.602541
H	0.666039	−0.756075	1.349332
Cl	3.582432	−1.130658	0.469854
TS28			
0	2		
C	−2.575716	1.136342	0.442808
C	−1.414824	1.827828	0.518496
C	−0.305490	0.989589	0.014245
C	−0.979581	−0.259194	−0.447051
C	−2.319580	−0.132642	−0.145707
H	−3.540070	1.457648	0.797453
H	−1.272724	2.808625	0.938996
C	2.017171	0.975646	−0.793034
C	2.301374	−0.148214	0.037413
C	1.410045	−0.574939	0.994162
C	0.130007	−0.033864	1.047689
C	0.808419	1.563933	−0.769361
H	2.810800	1.375249	−1.404309
H	0.617497	2.457041	−1.342637
H	−0.511824	−0.190919	1.898471
H	1.715447	−1.299191	1.732567
Cl	3.866538	−0.860297	−0.063467
H	−0.502789	−1.017776	−1.039155
Cl	−3.498731	−1.354855	−0.347083
TS29			
0	2		
C	−2.553365	1.092276	0.499900
C	−1.450846	1.864226	0.378540
C	−0.212447	1.127441	0.116417
C	−0.939098	−0.629073	0.071757
C	−2.272561	−0.257552	0.141770

Table S3. *Cont.*

TS	x	y	z
H	−3.541035	1.448102	0.737494
H	−1.441079	2.936848	0.495287
C	1.977408	0.932792	−0.897251
C	2.285397	−0.131946	0.016727
C	1.424623	−0.564389	0.945893
C	0.058712	−0.010527	1.022129
C	0.773024	1.547632	−0.813876
H	2.722062	1.242606	−1.610811
H	0.541961	2.377779	−1.464592
H	−0.325188	0.095488	2.034209
H	1.720211	−1.319131	1.657365
Cl	3.874210	−0.807713	−0.083069
H	−0.601363	−1.421968	−0.576589
Cl	−3.498954	−1.261993	−0.514837
TS30			
0	2		
C	2.412943	1.206880	−0.026383
C	1.233718	1.892287	−0.032025
C	0.000000	1.221915	0.023076
C	1.239059	−0.902591	0.086079
C	2.396769	−0.203752	0.000063
H	3.357615	1.722600	−0.077249
H	1.239739	2.969966	−0.098488
C	−1.239060	−0.902592	0.086079
C	−2.396770	−0.203753	0.000063
C	−2.412942	1.206880	−0.026380
C	−1.233718	1.892287	−0.032023
C	−0.000001	−0.199512	0.217887
H	−1.242577	−1.980341	0.106613
H	0.000029	−0.131379	1.873952
H	−1.239738	2.969966	−0.098484
H	−3.357614	1.722600	−0.077244
Cl	−3.904185	−1.039451	−0.103363
H	1.242576	−1.980341	0.106614
TS31			
0	2		
C	−2.010323	1.272321	0.261382
C	−0.696782	1.557585	0.259112
C	−0.249847	−0.768341	−0.585542
C	−1.625437	−0.980420	−0.601495
C	−2.478136	−0.026285	−0.100980
H	−2.738138	2.036347	0.483996
H	−0.348292	2.558749	0.456662
C	2.548488	0.007653	0.074751

Table S3. *Cont.*

TS	x	y	z
C	1.933402	−0.824977	1.046409
C	0.583371	−0.525950	1.060663
C	0.314806	0.507951	0.019527
C	1.649185	0.820748	−0.528884
H	1.836174	1.514688	−1.328700
H	0.419829	−1.391245	−1.154641
H	−0.151201	−0.832453	1.782907
H	−2.023053	−1.877706	−1.048733
Cl	−4.175187	−0.317523	−0.064196
Cl	4.219741	−0.075137	−0.303610
H	2.434887	−1.574868	1.631563
TS32			
0	2		
C	−1.991644	1.265325	0.410015
C	−0.668455	1.554807	0.336102
C	−0.182521	−0.728533	−0.577890
C	−1.641356	−0.930360	−0.588084
C	−2.459920	0.000182	−0.079474
H	−2.704098	1.977414	0.790811
H	−0.306051	2.522459	0.649211
C	2.521419	−0.019642	0.033491
C	1.887532	−0.995532	0.855028
C	0.507371	−1.028704	0.739428
C	0.269506	0.612077	−0.154610
C	1.677018	0.921694	−0.435813
H	1.972758	1.793522	−0.995822
H	0.341112	−1.149879	−1.432012
H	−0.124409	−1.364223	1.547685
H	−2.041133	−1.835237	−1.017506
Cl	−4.169634	−0.258602	−0.059247
Cl	4.224725	0.002456	−0.200969
H	2.411576	−1.497463	1.652144
TS33			
0	2		
C	−0.763625	−1.700852	0.009038
C	0.211463	−0.668520	0.190550
C	−1.598398	0.968235	0.018007
C	−2.498187	−0.055129	−0.023002
C	−2.083890	−1.402988	−0.066179
H	−0.431079	−2.727010	−0.020388
H	0.182423	−0.692077	1.852529
H	−2.824023	−2.179589	−0.169955
C	2.081527	1.412223	0.035393
C	2.495811	0.064466	−0.001601

Table S3. *Cont.*

TS	x	y	z
C	1.604560	−0.955161	0.041054
C	−0.221534	0.691638	0.058151
C	0.750228	1.708279	0.046917
H	2.823668	2.193300	0.018344
H	0.428454	2.738706	0.028963
H	−1.938855	1.991492	−0.003143
Cl	−4.188781	0.282809	−0.083409
H	1.935433	−1.980813	0.014200
Cl	4.186208	−0.266171	−0.126621
TS34			
0	2		
C	0.938369	1.701206	1.021574
C	−0.136048	0.824826	1.141302
C	1.259824	−0.915289	−0.027142
C	2.228797	0.010181	−0.128634
C	2.077200	1.351102	0.333890
H	0.886553	2.660234	1.513970
H	−0.941864	1.000944	1.833994
H	2.901229	2.036588	0.230782
C	−2.260341	−1.263860	0.712607
C	−2.269189	−0.194606	−0.227227
C	−0.988485	0.264021	−0.442761
C	−0.082927	−0.521601	0.447696
C	−0.985554	−1.498574	1.098510
H	−3.143627	−1.763904	1.071345
H	−0.651068	−2.223975	1.820171
H	1.428622	−1.938560	−0.318499
Cl	3.774921	−0.429666	−0.769798
H	−0.645406	0.943080	−1.200799
Cl	−3.688116	0.473264	−0.908429
TS35			
0	2		
C	0.961062	1.726154	0.930021
C	−0.204034	0.835812	1.094301
C	1.250461	−0.953547	0.080481
C	2.230884	−0.041540	−0.112601
C	2.087617	1.318244	0.333023
H	0.889939	2.732307	1.315251
H	−0.745187	0.964143	2.028966
H	2.924079	1.987929	0.210906
C	−2.274232	−1.145565	0.810473
C	−2.289270	0.012696	−0.016912
C	−1.110618	0.738568	−0.108306
C	0.021812	−0.565090	0.676956

Table S3. *Cont.*

TS	x	y	z
C	−1.006656	−1.521084	1.095458
H	−3.161910	−1.690398	1.082143
H	−0.740296	−2.425295	1.620298
H	1.386261	−1.980628	−0.220181
Cl	3.713015	−0.484900	−0.868161
H	−0.865067	1.320690	−0.982394
Cl	−3.577131	0.288627	−1.117272
TS36			
0	2		
C	1.633169	−0.387632	1.274591
C	0.371348	0.170271	1.242699
C	0.177800	0.840159	−0.079439
C	1.482168	0.673438	−0.756128
C	2.280764	−0.061602	0.051455
H	2.053664	−0.989472	2.060244
H	−0.329710	0.261834	2.052400
C	−2.681940	0.907989	0.070415
C	−2.052073	−0.300984	−0.092617
C	−0.669205	−0.411596	−0.207524
C	−0.582899	2.104344	−0.161961
C	−1.921825	2.111316	−0.031794
H	−3.752991	0.947977	0.176063
H	−2.454904	3.049733	−0.043230
H	−0.026892	3.017765	−0.299618
H	−0.201905	−1.317533	−0.553698
Cl	−2.992241	−1.746679	−0.219907
H	1.703699	1.017867	−1.750320
Cl	3.861958	−0.598168	−0.338918
TS37			
0	2		
C	1.512295	−0.679688	1.110207
C	0.144704	−0.445400	1.066389
C	0.141402	0.918986	−0.227942
C	1.547267	0.838750	−0.643150
C	2.238874	−0.103547	0.031559
H	2.002242	−1.067918	1.988202
H	−0.459544	−0.425538	1.960205
C	−2.656948	0.944883	0.109823
C	−2.051235	−0.227010	−0.129102
C	−0.592136	−0.364318	−0.251417
C	−0.549251	2.136761	−0.001677
C	−1.893162	2.155426	0.184183
H	−3.729750	0.981980	0.212895
H	−2.408475	3.084606	0.365598

Table S3. *Cont.*

TS	x	y	z
H	0.022273	3.052315	0.012377
H	−0.263035	−1.094390	−0.986068
Cl	−2.954372	−1.682406	−0.297394
H	1.947780	1.454601	−1.431333
Cl	3.885998	−0.496106	−0.268200
TS38			
0	2		
C	1.952322	2.260542	−0.050060
C	0.588521	2.257147	−0.007832
C	−0.123729	1.046798	0.028300
C	2.014517	−0.133496	−0.017378
C	2.672929	1.051436	−0.082275
H	2.493197	3.192730	−0.088762
H	0.037717	3.185251	−0.023161
H	3.747517	1.060301	−0.164871
C	−1.502698	−1.394569	−0.065820
C	−2.194314	−0.167305	−0.006682
C	−1.529882	1.020911	0.018999
C	0.591140	−0.193586	0.150132
C	−0.148235	−1.407719	−0.018630
H	−2.061600	−2.311345	−0.159236
H	0.389830	−2.340034	−0.059427
H	0.623603	−0.249573	1.820492
Cl	2.913925	−1.603254	−0.030083
H	−2.076709	1.950611	0.008143
Cl	−3.918454	−0.192563	−0.030253
TS39			
0	2		
C	1.986140	0.589262	1.157778
C	0.609352	0.681777	1.093929
C	0.136313	−0.078817	−0.100530
C	1.368596	−0.674874	−0.656731
C	2.413497	−0.239140	0.085032
H	2.634516	1.075292	1.864732
H	−0.059716	1.080729	1.834491
C	−2.412169	1.218963	−0.219814
C	−2.301448	−0.190774	−0.025731
C	−1.126943	−0.842155	−0.036599
C	−0.021390	1.369291	−0.525060
C	−1.290658	1.944906	−0.540686
H	−3.389316	1.671543	−0.218734
H	−1.393034	2.973131	−0.852701
H	0.830441	1.855868	−0.969642
H	−1.080088	−1.915895	0.036504

Table S3. *Cont.*

TS	x	y	z
Cl	−3.777706	−1.077794	0.142887
H	1.409725	−1.276532	−1.546966
Cl	4.064749	−0.577193	−0.233311
TS40			
0	2		
C	1.957387	0.733643	1.038005
C	0.649555	1.166297	0.875321
C	0.080604	−0.138098	−0.350192
C	1.376341	−0.768064	−0.633290
C	2.388927	−0.204882	0.058311
H	2.514239	0.908551	1.944222
H	0.051816	1.497931	1.710604
C	−2.405250	1.181612	−0.276850
C	−2.301719	−0.232363	−0.045658
C	−1.110544	−0.872793	−0.116087
C	0.039698	1.330122	−0.502693
C	−1.311894	1.913598	−0.530759
H	−3.386327	1.629278	−0.287116
H	−1.410162	2.960710	−0.774788
H	0.736879	1.745301	−1.225804
H	−1.052180	−1.943111	0.004319
Cl	−3.753143	−1.102229	0.273743
H	1.483008	−1.566831	−1.348488
Cl	4.040443	−0.655786	−0.104307
TS41			
0	2		
C	−2.412388	1.195295	−0.092660
C	−1.243256	1.879930	−0.048340
C	0.000000	−0.232668	0.048025
C	−1.228482	−0.915211	0.039663
C	−2.396213	−0.213404	−0.009326
H	−3.354873	1.706257	−0.202465
H	−1.238115	2.957440	−0.108471
C	2.412388	1.195295	−0.092664
C	2.396213	−0.213404	−0.009326
C	1.228482	−0.915211	0.039665
C	0.000000	1.198612	0.139583
C	1.243256	1.879930	−0.048346
H	3.354873	1.706257	−0.202472
H	1.238114	2.957440	−0.108481
H	0.000002	1.265443	1.806649
H	1.242391	−1.993595	0.049019
Cl	3.903367	−1.051783	−0.031777
H	−1.242390	−1.993595	0.049016

Table S3. *Cont.*

TS	x	y	z
Cl	−3.903367	−1.051784	−0.031778
TS42			
0	2		
C	1.959614	1.849991	−0.855548
C	0.752543	2.173270	−0.356273
C	0.593749	−0.156223	0.581810
C	1.852383	−0.391947	0.040244
C	2.509255	0.540029	−0.727035
H	2.558070	2.606830	−1.339378
H	0.373907	3.181335	−0.410071
H	3.474628	0.310062	−1.145205
C	−2.240391	0.738736	1.078403
C	−2.062024	−0.066958	−0.082989
C	−0.821131	0.157916	−0.634322
C	−0.115685	1.139026	0.245590
C	−1.125622	1.477613	1.275497
H	−3.117418	0.718071	1.702405
H	−0.938998	2.165012	2.082672
H	0.178370	−0.790275	1.346339
Cl	2.622481	−1.896702	0.405145
H	−0.447588	−0.175256	−1.584675
Cl	−3.204664	−1.208268	−0.643048
TS43			
0	2		
C	1.944586	1.843306	−0.952552
C	0.745405	2.188235	−0.422728
C	0.508395	−0.097980	0.604701
C	1.849291	−0.346092	0.047043
C	2.505734	0.545983	−0.706381
H	2.502519	2.544747	−1.551355
H	0.336421	3.174607	−0.581332
H	3.476688	0.298602	−1.104978
C	−2.210843	0.729675	1.053532
C	−1.931216	−0.282258	0.094793
C	−0.619849	−0.387807	−0.349480
C	−0.007218	1.267321	0.351383
C	−1.203929	1.631095	1.113958
H	−3.145795	0.802852	1.581740
H	−1.232162	2.540286	1.694151
H	0.359060	−0.491895	1.607234
Cl	2.530496	−1.884335	0.410013
H	−0.376674	−0.776771	−1.325278
Cl	−3.201213	−1.094564	−0.723296

Table S3. *Cont.*

TS	x	y	z
TS44			
0	2		
C	−1.990094	1.464169	0.037360
C	−0.806112	2.137821	0.026981
C	0.426643	1.460050	0.022165
C	−0.829878	−0.656806	0.037223
C	−1.982674	0.054108	0.007216
H	−2.931166	1.988888	0.031879
H	−0.802467	3.217213	0.002262
H	−0.841858	−1.732903	0.017004
C	2.829512	1.457928	−0.102667
C	2.838237	0.049979	−0.112386
C	1.673735	−0.640545	−0.013835
C	0.417948	0.030063	0.166856
C	1.650729	2.143273	−0.048130
H	3.766456	1.987710	−0.168182
H	0.464081	0.001574	1.832639
H	1.642296	3.222262	−0.080433
H	3.768243	−0.487095	−0.204381
Cl	−3.498322	−0.766718	−0.091480
Cl	1.708095	−2.362569	−0.000020
TS45			
0	2		
C	−2.932142	−1.283788	−0.842954
C	−2.214305	−1.660259	0.224707
C	−1.393831	−0.519129	0.686381
C	−1.701123	0.536508	−0.309858
C	−2.617506	0.095649	−1.180565
H	−3.641162	−1.891784	−1.379785
H	−2.225796	−2.619610	0.713574
H	−3.048141	0.662123	−1.987761
C	1.946211	0.503801	1.216940
C	2.022642	−0.319328	0.018485
C	0.933833	−1.080389	−0.109997
C	0.047662	−0.809898	1.061712
C	0.779523	0.255476	1.814257
H	2.713337	1.191267	1.528810
H	0.405016	0.709251	2.716387
H	0.031507	−1.702472	1.698235
H	−1.918478	−0.137681	1.710218
Cl	−1.011132	2.096594	−0.265895
H	0.709133	−1.778000	−0.896978
Cl	3.367214	−0.271371	−1.042992
H	−2.304599	0.226259	2.673743

Table S3. *Cont.*

TS	x	y	z
TS46			
0	2		
C	2.429726	−2.064838	−0.315749
C	1.935397	−1.296997	−1.296986
C	1.270107	−0.113490	−0.708156
C	1.438996	−0.342419	0.756503
C	2.126829	−1.466035	0.977017
H	2.972700	−2.986737	−0.442443
H	1.995970	−1.474873	−2.357050
H	2.417945	−1.855993	1.936719
C	−1.914351	1.372122	−0.362088
C	−2.188321	−0.056527	−0.297732
C	−1.165603	−0.763271	−0.783587
C	−0.120895	0.203363	−1.237550
C	−0.692924	1.536079	−0.874592
H	−2.600432	2.134673	−0.036028
H	−0.172356	2.462077	−1.050619
H	−0.056499	0.166410	−2.330726
H	1.940400	0.808241	−0.975003
Cl	0.890089	0.765391	1.936320
H	−1.082184	−1.833135	−0.855258
Cl	−3.656708	−0.682326	0.326694
O	2.547442	2.107839	−0.939816
H	2.523672	2.256592	0.015217
TS47			
0	2		
C	1.903351	−1.555587	−0.346038
C	0.668827	−1.522703	−0.853424
C	0.333169	−0.123559	−1.259480
C	1.526794	0.663064	−0.829275
C	2.417796	−0.195249	−0.324082
H	2.445815	−2.413872	0.010592
H	−0.006213	−2.352667	−0.979445
C	−1.821704	2.545058	−0.344955
C	−1.601213	1.942808	0.943278
C	−1.084733	0.716895	0.745074
C	−0.982866	0.433137	−0.733253
C	−1.443283	1.693504	−1.324944
H	−2.224419	3.533716	−0.491607
H	−1.789190	−0.323755	−0.954656
H	−1.494860	1.869139	−2.386071
Cl	−3.141892	−1.684681	−0.334357
H	0.270363	−0.104810	−2.353121
H	−1.820594	2.388931	1.897056

Table S3. *Cont.*

TS	x	y	z
H	1.630381	1.728528	−0.934053
Cl	−0.626753	−0.380820	1.949539
Cl	3.974050	0.188477	0.276225
TS48			
0	2		
C	−1.498202	2.355067	0.076871
C	−0.368765	1.644389	−0.140952
C	−0.739651	0.249130	−0.231018
C	−2.157397	0.196225	−0.032125
C	−2.629284	1.454717	0.145608
H	−1.563724	3.423723	0.186729
H	0.634321	2.019392	−0.235634
H	−3.655529	1.731961	0.309512
C	1.496255	−0.591673	1.190365
C	2.221652	−0.349643	−0.006830
C	1.449442	−0.553337	−1.101280
C	0.101459	−0.925890	−0.637158
C	0.215631	−0.986737	0.833669
H	1.877997	−0.462602	2.187199
H	−0.535586	−1.403242	1.480338
H	−0.451219	−1.693681	−1.163819
Cl	−3.056869	−1.264367	−0.048001
H	1.729805	−0.392017	−2.126470
Cl	3.846110	0.195130	−0.023808
TS49			
0	2		
C	−3.444066	−0.123591	0.148144
C	−2.603914	−1.148655	−0.127363
C	−1.268377	−0.621734	−0.257916
C	−1.384641	0.806401	−0.030477
C	−2.681696	1.101316	0.213084
H	−4.508679	−0.191669	0.291686
H	−2.858491	−2.187320	−0.252773
H	−3.071116	2.083162	0.415527
C	1.135881	−1.013167	1.142435
C	1.815021	−0.542839	−0.008958
C	1.106064	−0.796533	−1.135895
C	−0.135985	−1.483262	−0.743623
C	−0.030456	−1.640751	0.722214
H	1.469780	−0.887535	2.156763
H	−0.655405	−2.281616	1.315460
H	−0.474730	−2.312544	−1.351336
Cl	−0.123477	1.963765	−0.058208
H	1.351547	−0.478894	−2.132911

Table S3. *Cont.*

TS	x	y	z
Cl	3.282307	0.332311	0.059839
TS50			
0	2		
C	−1.524221	2.288687	−0.299128
C	−0.470477	1.748256	0.356544
C	−0.701820	0.327889	0.460443
C	−1.951450	0.073978	−0.195637
C	−2.462072	1.244749	−0.649848
H	−1.658438	3.330756	−0.532641
H	0.392663	2.259387	0.742570
H	−3.391865	1.372556	−1.175041
C	2.242078	0.104728	1.285530
C	1.961496	−0.313106	−0.047298
C	0.686537	−0.844419	−0.122822
C	0.079194	−0.704233	1.216283
C	1.156463	−0.137809	2.054017
H	3.162205	0.569856	1.595317
H	1.041717	0.102674	3.096594
H	−0.527954	−1.509794	1.610009
Cl	−2.642582	−1.490598	−0.332424
H	0.254870	−1.381137	−0.947488
Cl	3.032725	−0.125674	−1.358036
TS51			
0	2		
C	−2.927581	−1.006321	−0.795219
C	−2.014635	−1.630778	−0.012669
C	−1.042660	−0.659248	0.418999
C	−1.450618	0.596767	−0.176856
C	−2.574481	0.388738	−0.901235
H	−3.784020	−1.459768	−1.263641
H	−1.996387	−2.670044	0.268462
H	−3.109637	1.141244	−1.452539
C	1.795285	0.447438	1.161122
C	1.733473	−0.384741	0.010843
C	0.718443	−1.317536	0.144283
C	0.023777	−1.014988	1.414204
C	0.799911	0.094299	2.005155
H	2.483796	1.265411	1.283061
H	0.546249	0.576056	2.933010
H	−0.268596	−1.828978	2.065843
Cl	−0.686905	2.121753	−0.010321
H	0.570917	−2.189947	−0.462943
Cl	2.757034	−0.234445	−1.341621

Table S3. *Cont.*

TS	x	y	z
TS52			
0	2		
C	−1.575550	2.319182	−0.052441
C	−0.401430	1.685384	0.012007
C	−0.645790	0.216724	0.042201
C	−2.129747	0.129677	−0.001364
C	−2.665982	1.350008	−0.056429
H	−1.719199	3.385785	−0.099989
H	0.582932	2.118224	0.026360
H	−3.716312	1.581024	−0.092648
C	1.479786	−0.411727	1.192619
C	2.174705	−0.280564	0.048714
C	1.429104	−0.609651	−1.134562
C	0.066304	−0.644634	−0.993475
C	0.064509	−0.658260	0.983588
H	1.913393	−0.353981	2.177814
H	−0.474740	−1.439446	1.499273
H	−0.560236	−1.213250	−1.663259
Cl	−2.951323	−1.368153	0.029740
H	1.921933	−1.005722	−2.008161
Cl	3.849957	0.094655	−0.034713
TS53			
0	2		
C	−3.397721	−0.269725	−0.013388
C	−2.496101	−1.253645	−0.015792
C	−1.124105	−0.678900	−0.032493
C	−1.393512	0.795030	−0.022276
C	−2.711734	1.014288	−0.010297
H	−4.468278	−0.386753	−0.014218
H	−2.679926	−2.315158	−0.024868
H	−3.185628	1.980413	0.012033
C	1.123603	−0.933547	1.140588
C	1.788749	−0.506730	−0.055763
C	1.129795	−0.808518	−1.185812
C	−0.193698	−1.354949	−0.952833
C	−0.198531	−1.287730	1.011653
H	1.679861	−1.150257	2.038705
H	−0.663860	−1.946212	1.728741
H	−0.533305	−2.243655	−1.464292
Cl	−0.200021	2.012063	0.037735
H	1.525453	−0.670032	−2.178621
Cl	3.327387	0.249008	0.004677
TS54			
0	2		

Table S3. *Cont.*

TS	x	y	z
C	−1.856733	1.909837	−1.063904
C	−0.656404	1.757765	−0.498414
C	−0.577553	0.402786	0.120671
C	−1.920376	−0.165875	−0.174267
C	−2.657831	0.707306	−0.861498
H	−2.201818	2.778582	−1.599454
H	0.157423	2.460411	−0.479709
H	−3.666510	0.554803	−1.203856
C	2.148974	0.893705	0.917523
C	1.863959	−0.052466	−0.124358
C	0.576998	−0.496889	−0.279705
C	−0.049224	0.223689	1.484081
C	1.158119	0.942902	1.831150
H	3.092995	1.404643	0.995549
H	1.235595	1.470588	2.769484
H	−0.576808	−0.409272	2.182478
Cl	−2.373802	−1.730826	0.340364
H	0.349804	−1.438710	−0.753191
Cl	3.163787	−0.831386	−0.929716
TS55			
0	2		
C	−2.917778	−1.228408	−0.536832
C	−1.814602	−1.756963	−0.004965
C	−0.810454	−0.685807	0.249266
C	−1.525531	0.538612	−0.243949
C	−2.738392	0.207757	−0.694923
H	−3.811223	−1.762613	−0.812830
H	−1.631995	−2.789634	0.241682
H	−3.456683	0.888303	−1.118709
C	1.690452	0.616678	1.106696
C	1.645573	−0.198256	−0.069771
C	0.568791	−1.030807	−0.273316
C	−0.150439	−0.794443	1.566659
C	0.774204	0.226059	2.011692
H	2.416929	1.397725	1.247116
H	0.712170	0.624433	3.012618
H	−0.403816	−1.634017	2.197096
Cl	−0.864706	2.111415	−0.286637
H	0.659374	−1.902706	−0.901317
Cl	3.052018	−0.357769	−1.038598
TS56			
0	2		
C	3.233658	−0.730370	−0.317751
C	2.240485	−1.560455	0.097295

Table S3. *Cont.*

TS	x	y	z
C	0.991975	−0.785912	0.258013
C	1.425530	0.614325	−0.024376
C	2.763538	0.602570	−0.397154
H	4.231017	−1.039101	−0.583581
H	2.284486	−2.631630	0.195089
H	3.323116	1.467099	−0.707073
C	−1.796356	−0.334265	−0.118661
C	−1.303108	−0.801420	1.136688
C	0.000924	−1.073576	1.311961
C	0.381852	−0.491407	−1.107026
C	−0.985095	−0.259457	−1.223204
H	−1.412173	−0.064829	−2.194084
H	0.996238	−0.670277	−1.973158
Cl	0.566234	2.012874	0.453333
H	0.357783	−1.494375	2.238321
H	−2.010508	−0.977717	1.931400
Cl	−3.477433	0.006575	−0.253429
TS57			
0	2		
C	−3.231642	−0.651023	0.285537
C	−2.302785	−1.608845	0.056124
C	−0.934469	−1.090186	−0.055325
C	−1.312050	0.746014	0.213408
C	−2.698558	0.664284	0.190143
H	−4.279785	−0.848074	0.438312
H	−2.507229	−2.666184	−0.006380
H	−3.294929	1.527034	−0.058580
C	1.756293	−0.326034	0.131852
C	1.275293	−1.008114	−1.032371
C	−0.018408	−1.421981	−1.076553
C	−0.493070	−0.210502	1.057461
C	0.957520	0.017492	1.150837
H	1.362801	0.458776	2.046422
H	−0.977600	−0.399305	2.011515
Cl	−0.506133	2.048021	−0.577612
H	−0.369828	−2.010365	−1.910681
H	1.965805	−1.242332	−1.824859
Cl	3.453899	−0.017797	0.211586
TS58			
0	2		
C	1.923149	2.104247	0.391689
C	0.651360	1.600179	0.597627
C	0.667305	0.138561	0.301165
C	2.091082	−0.112634	−0.000945

Table S3. *Cont.*

TS	x	y	z
C	2.795058	1.045501	0.023609
H	2.202681	3.141005	0.464637
H	−0.197118	2.094388	1.034099
H	3.836971	1.145438	−0.227240
C	−2.137994	−0.090212	−0.049553
C	−1.477728	−0.855348	0.956988
C	−0.141173	−0.810548	1.090598
C	−0.048447	0.716112	−0.906409
C	−1.428958	0.591199	−1.011216
H	−1.937988	0.993771	−1.872537
H	0.553220	1.056738	−1.732147
Cl	2.671606	−1.664309	−0.404008
H	0.370856	−1.453709	1.788407
H	−2.069468	−1.515438	1.571099
Cl	−3.855198	−0.184193	−0.148206
TS59			
0	2		
C	1.796664	2.098756	0.520274
C	0.448672	1.793454	0.400797
C	0.632574	−0.074564	0.094056
C	2.091218	−0.089989	−0.051586
C	2.708366	1.108578	0.064835
H	2.122229	2.953447	1.092666
H	−0.298463	2.227006	1.047966
H	3.766706	1.257798	−0.061088
C	−2.150290	−0.129407	−0.047998
C	−1.456909	−1.044699	0.813370
C	−0.102100	−1.023001	0.845929
C	−0.057228	0.929414	−0.738164
C	−1.520604	0.770806	−0.814311
H	−2.076479	1.386061	−1.503920
H	0.422660	1.161975	−1.686129
Cl	2.889745	−1.574193	−0.365973
H	0.443510	−1.736937	1.443946
H	−2.024241	−1.755452	1.389882
Cl	−3.871986	−0.280512	−0.119118
TS60			
0	2		
C	0.604314	2.253348	−0.028511
C	−0.109056	1.028208	0.164672
C	2.009164	−0.151073	−0.011354
C	2.676447	1.037476	−0.069027
C	1.957468	2.248393	−0.113993
H	0.046207	3.176180	−0.055852

Table S3. *Cont.*

TS	x	y	z
H	−0.071768	1.067676	1.823183
H	3.752993	1.041279	−0.115701
H	2.499750	3.174069	−0.225536
Cl	2.925451	−1.616611	−0.033244
C	−1.498098	−1.402833	0.049661
C	−2.190520	−0.176206	0.008340
C	−1.533643	1.007686	0.032696
C	0.601470	−0.214681	0.036822
C	−0.133914	−1.412106	0.046541
H	−2.056433	−2.324475	0.049268
H	0.394522	−2.351283	0.035844
H	−2.071852	1.941339	0.003319
Cl	−3.913405	−0.208922	−0.096733
TS61			
0	2		
C	−2.917422	−1.288420	−0.291959
C	−1.694819	−1.853515	−0.113823
C	−0.676266	−0.787584	0.003459
C	−1.464239	0.457646	−0.236729
C	−2.803702	0.121700	−0.373378
H	−3.850285	−1.825603	−0.334522
H	−1.473834	−2.895980	0.038607
H	−3.604828	0.826994	−0.505641
C	1.577307	0.471388	1.249325
C	1.710659	−0.279364	0.042654
C	0.689548	−0.934144	−0.534317
C	−0.749780	−0.151312	1.384966
C	0.380172	0.462149	1.919797
H	2.444928	0.960191	1.658837
H	0.316452	0.902648	2.902961
H	−1.618503	−0.366763	1.983636
Cl	−0.788713	1.958897	−0.693739
H	0.839196	−1.549991	−1.405272
Cl	3.296838	−0.392000	−0.637941
TS62			
0	2		
C	−2.826882	−1.393192	−0.273974
C	−1.644663	−1.984917	0.020184
C	−0.551761	−1.032496	0.250809
C	−1.511701	0.567525	−0.019764
C	−2.703398	0.009161	−0.470287
H	−3.748762	−1.928118	−0.431271
H	−1.490653	−3.047947	0.118933
H	−3.383663	0.583328	−1.078098

Table S3. *Cont.*

TS	x	y	z
C	1.544214	0.622094	1.160274
C	1.729247	−0.301320	0.078569
C	0.738235	−1.140796	−0.314449
C	−0.839528	0.045519	1.231776
C	0.343729	0.760323	1.737909
H	2.401316	1.166596	1.523110
H	0.225111	1.398729	2.599286
H	−1.573544	−0.208703	1.991633
Cl	−0.880435	1.983643	−0.775614
H	0.921636	−1.890781	−1.067619
Cl	3.291235	−0.394497	−0.639813
TS63			
0	2		
C	2.250353	−1.456352	0.226142
C	1.877956	−0.207410	−0.139812
C	0.584888	0.154834	0.483355
C	0.299984	−1.034488	1.343518
C	1.288458	−1.974940	1.136439
H	3.117351	−1.979086	−0.139327
H	−0.488209	−1.045535	2.073951
H	1.314434	−2.959358	1.570720
C	−2.061420	1.225545	0.553634
C	−1.829685	0.063900	−0.143633
C	−0.563055	−0.496353	−0.257563
C	0.311209	1.522044	0.968013
C	−0.952718	1.976354	1.045954
H	−3.062325	1.614188	0.636525
H	1.150117	2.125091	1.276298
H	−1.143356	2.957045	1.454321
Cl	−3.137592	−0.718471	−0.961421
H	−0.346545	−1.274722	−0.968554
Cl	2.680104	0.831622	−1.226827
TS64			
0	2		
C	0.966834	−2.041076	1.026586
C	−0.181251	−1.257927	1.000801
C	0.577413	0.294804	0.213294
C	1.882578	−0.289572	−0.111254
C	2.050233	−1.589471	0.230221
H	1.076786	−2.838732	1.744565
H	−0.884568	−1.238597	1.818931
H	2.936530	−2.160879	0.015394
C	−1.991970	1.415040	0.383787
C	−1.859396	0.199499	−0.165440

Table S3. *Cont.*

TS	x	y	z
C	−0.571974	−0.507873	−0.251479
C	0.395960	1.590541	0.754996
C	−0.843999	2.129310	0.860687
H	−2.966544	1.873513	0.438140
H	−0.981122	3.110565	1.284958
H	1.270223	2.137852	1.072167
Cl	3.064972	0.675470	−0.891285
H	−0.426920	−1.075532	−1.167691
Cl	−3.216205	−0.644167	−0.806342
TS65			
0	2		
C	−0.669671	−2.422163	−0.089598
C	0.531267	−1.796003	−0.035116
C	−0.615529	0.391808	0.015518
C	−1.831428	−0.326001	0.002332
C	−1.869782	−1.688250	−0.032771
H	−0.715526	−3.495522	−0.184770
H	1.451036	−2.354227	−0.069848
H	−2.820670	−2.194617	−0.053773
C	1.860820	1.698830	−0.071236
C	1.828565	0.343659	−0.014800
C	0.596619	−0.377604	0.138151
C	−0.546193	1.793483	−0.008351
C	0.660293	2.430215	−0.039972
H	2.807863	2.207949	−0.145072
H	0.701234	3.507402	−0.069060
H	−1.461527	2.360802	−0.020339
Cl	−3.328638	0.538206	−0.005581
H	0.640678	−0.419008	1.805230
Cl	3.312588	−0.532360	−0.020732
TS66			
0	2		
C	2.211177	−1.583816	−1.057380
C	0.846186	−1.521328	−0.853482
C	0.527247	−0.234937	−0.165765
C	1.846748	0.429438	−0.101602
C	2.813198	−0.383675	−0.591121
H	2.742094	−2.420388	−1.477781
H	0.086643	−2.183648	−1.226455
H	3.866976	−0.164525	−0.594826
C	−2.027212	−1.053687	0.808323
C	−1.889273	0.056447	−0.076912
C	−0.699039	0.517283	−0.495136
C	0.375173	−1.243654	0.954820

Table S3. *Cont.*

TS	x	y	z
C	−0.901856	−1.617339	1.362212
H	−3.013383	−1.357479	1.116034
H	−1.004156	−2.337146	2.159959
H	1.258094	−1.519582	1.505917
Cl	2.050987	1.971341	0.595031
H	−0.616159	1.409669	−1.092871
Cl	−3.341234	0.874818	−0.541721
TS67			
0	2		
C	−2.139111	1.611125	−1.032203
C	−0.828915	1.831356	−0.627653
C	−0.477062	0.103418	0.072454
C	−1.846072	−0.413470	−0.021511
C	−2.767641	0.448529	−0.512801
H	−2.576363	2.186908	−1.833033
H	−0.127321	2.388904	−1.229239
H	−3.817238	0.226094	−0.597199
C	2.077257	1.078238	0.697099
C	1.903201	−0.149057	−0.030357
C	0.670787	−0.638058	−0.301605
C	−0.361343	1.408778	0.749867
C	1.017776	1.793266	1.098411
H	3.079668	1.384681	0.950844
H	1.162670	2.674306	1.705434
H	−1.101164	1.603210	1.523152
Cl	−2.180394	−2.013777	0.494077
H	0.544769	−1.586537	−0.799239
Cl	3.318142	−1.005183	−0.510014
TS68			
0	2		
C	−2.832120	1.440445	−0.110408
C	−1.665506	2.128827	−0.049851
C	−0.412297	0.003499	0.024787
C	−1.659239	−0.655410	0.002547
C	−2.836587	0.032560	−0.047522
H	−3.770011	1.962429	−0.215933
H	−1.650172	3.206925	−0.090505
H	−3.767349	−0.509940	−0.074622
C	1.988586	1.457066	−0.074067
C	1.982990	0.048411	−0.009074
C	0.821957	−0.665975	0.020999
C	−0.428955	1.436317	0.134926
C	0.812725	2.128184	−0.030006
H	2.926872	1.978739	−0.168415

Table S3. *Cont.*

TS	x	y	z
H	0.796141	3.206280	−0.074371
H	−0.441606	1.480259	1.799513
Cl	−1.707070	−2.382684	0.003774
H	0.846259	−1.742611	0.021047
Cl	3.497102	−0.776470	−0.025580
TS69			
0	2		
C	2.698885	−1.029721	−0.920603
C	1.988220	−0.087273	−1.591316
C	0.920999	0.441035	−0.711213
C	1.184676	−0.256302	0.585338
C	2.229570	−1.150803	0.411394
H	3.488617	−1.629342	−1.341817
H	2.078293	0.181252	−2.629817
H	2.596658	−1.822922	1.166415
C	−1.783431	1.216088	−0.154204
C	−1.494204	−0.114006	−0.323135
C	−0.215185	−0.566127	−0.630821
C	0.489314	1.851569	−0.730967
C	−0.774564	2.189838	−0.417772
H	−2.782530	1.523740	0.104008
H	−1.055474	3.231710	−0.393904
H	1.225059	2.598421	−0.982177
Cl	0.585172	0.258061	2.099659
H	−0.041537	−1.562850	−0.997729
Cl	−2.760158	−1.286637	−0.218787
TS70			
0	2		
C	2.819101	−0.306892	−1.052605
C	2.263278	0.928437	−1.041880
C	0.974379	0.990867	−0.342627
C	0.844972	−0.824921	0.160330
C	2.116732	−1.230265	−0.233334
H	3.740145	−0.552031	−1.554863
H	2.680148	1.799376	−1.522725
H	2.574244	−2.103039	0.203273
C	−1.697778	1.205544	0.548328
C	−1.402409	0.259223	−0.353435
C	−0.024189	−0.021340	−0.779252
C	0.604091	1.972894	0.602724
C	−0.668339	2.048441	1.075369
H	−2.720214	1.353629	0.856827
H	−0.930663	2.785213	1.817120
H	1.360366	2.660883	0.948511

Table S3. *Cont.*

TS	x	y	z
Cl	0.171402	−1.395253	1.640869
H	0.065671	−0.359598	−1.807661
Cl	−2.627209	−0.705709	−1.078645
TS71			
0	2		
C	−1.789211	−2.374877	−0.037563
C	−2.406751	−1.164283	0.001014
C	−1.666730	0.029893	0.020747
C	0.374757	−1.304838	−0.023868
C	−0.388950	−2.430789	−0.073606
H	−2.359453	−3.289445	−0.065991
H	−3.483675	−1.093521	−0.008374
H	0.110643	−3.383176	−0.142328
C	−0.300876	2.442531	−0.081327
C	0.421654	1.290255	−0.022975
C	−0.221026	0.003686	0.133920
C	−2.363101	1.249710	−0.007216
C	−1.702548	2.437067	−0.051780
H	0.232749	3.376299	−0.149710
H	−2.239576	3.371268	−0.087398
H	−3.441859	1.217518	−0.017988
Cl	2.076891	−1.574961	−0.023553
H	−0.118070	0.000720	1.816037
Cl	2.132282	1.500268	−0.005172
TS72			
0	2		
C	−2.690924	−1.534177	−0.797768
C	−2.066364	−1.730662	0.367323
C	−1.433033	−0.454779	0.822785
C	−1.798959	0.479384	−0.288936
C	−2.528294	−0.148007	−1.215145
H	−3.239740	−2.273406	−1.357488
H	−2.000824	−2.650122	0.924581
H	−2.929384	0.292100	−2.111570
C	1.962680	0.708676	1.115548
C	2.025118	−0.213996	−0.008892
C	0.916925	−0.960890	−0.076152
C	0.062184	−0.580111	1.066310
C	0.789705	0.526963	1.731621
H	2.742635	1.403847	1.373583
H	0.423597	1.050780	2.598073
H	0.149512	−1.484627	1.851788
H	−1.907026	−0.115444	1.748478
Cl	−1.339232	2.123605	−0.295275

Table S3. *Cont.*

TS	x	y	z
H	0.675662	−1.717951	−0.800461
Cl	3.370684	−0.283975	−1.065337
H	0.116653	−2.333298	2.603254
TS73			
0	2		
C	2.708145	0.608791	−1.515891
C	2.094322	1.399017	−0.629764
C	1.443606	0.562222	0.425540
C	1.786777	−0.823753	−0.027773
C	2.521928	−0.786696	−1.142155
H	3.263772	0.933440	−2.380099
H	2.029085	2.473893	−0.633969
H	2.914137	−1.639157	−1.669379
C	−1.941296	−0.262724	1.266385
C	−1.981788	−0.114689	−0.184314
C	−0.886114	0.505444	−0.631420
C	−0.050999	0.836847	0.549414
C	−0.794272	0.265996	1.702652
H	−2.717440	−0.727788	1.849303
H	−0.450422	0.321712	2.721225
H	−0.103476	1.977392	0.702530
H	1.912175	0.755865	1.394162
Cl	1.307869	−2.223324	0.826899
H	−0.628035	0.737350	−1.649305
Cl	−3.291461	−0.687139	−1.126539
O	−0.112358	3.488611	0.694059
H	−1.001720	3.593538	0.330888
TS74			
0	2		
C	2.498687	1.158784	−1.516837
C	1.727675	1.735542	−0.588633
C	1.372264	0.733776	0.463330
C	2.065730	−0.495507	−0.036516
C	2.714542	−0.239210	−1.177528
H	2.906125	1.638319	−2.391175
H	1.369181	2.750895	−0.574712
H	3.304652	−0.941604	−1.740429
C	−1.569533	−1.135685	1.245295
C	−1.691032	−0.939293	−0.187154
C	−0.847284	0.009775	−0.601348
C	−0.128608	0.529952	0.607520
C	−0.627445	−0.295112	1.713906
H	−2.136914	−1.857331	1.808012
H	−0.577018	1.566163	0.801224

Table S3. *Cont.*

TS	x	y	z
H	−0.296379	−0.201783	2.733787
Cl	−1.503605	3.171180	0.343638
H	1.786758	1.029737	1.429962
H	−0.706732	0.385452	−1.598845
Cl	2.021480	−1.979298	0.802549
Cl	−2.796677	−1.824116	−1.146189
TS75			
0	2		
C	−2.904673	−1.316255	−0.281173
C	−2.315809	−1.405901	0.943915
C	−1.410810	−0.257442	1.126041
C	−1.612289	0.546975	−0.087869
C	−2.490277	−0.136186	−0.937786
H	−3.564916	−2.051640	−0.710451
H	−2.393286	−2.223068	1.639732
H	−2.777067	0.195083	−1.919731
C	1.604488	−1.380774	−0.759036
C	2.094761	−0.261361	0.007593
C	1.079588	0.328858	0.696531
C	−0.094574	−0.416926	0.394020
C	0.279590	−1.478660	−0.528068
H	2.207607	−1.999015	−1.399371
H	−0.401361	−2.193791	−0.951923
H	−1.321315	0.225090	2.088860
Cl	−1.278886	2.222720	−0.233578
H	1.149924	1.175894	1.353461
Cl	3.733029	0.220661	0.025014
TS76			
0	2		
C	1.866981	2.167033	−0.298534
C	1.602138	1.853625	0.999193
C	1.431725	0.393446	1.116605
C	1.723130	−0.103070	−0.237816
C	1.942889	0.990717	−1.079408
H	1.970007	3.165403	−0.690299
H	1.422657	2.542627	1.806154
H	2.128870	0.925783	−2.136544
C	−1.785838	−1.362708	0.620292
C	−2.079172	−0.065863	0.048133
C	−0.980545	0.714176	0.060130
C	0.071213	−0.080437	0.657068
C	−0.478760	−1.366147	0.979329
H	−2.498451	−2.161309	0.728130
H	0.064775	−2.175591	1.433071

Table S3. *Cont.*

TS	x	y	z
H	1.814914	−0.133773	1.979188
Cl	2.218346	−1.699011	−0.624103
H	−0.890994	1.721324	−0.300184
Cl	−3.623896	0.362006	−0.552925
TS77			
0	2		
C	−1.759982	2.051446	0.346907
C	−0.872963	1.668615	−0.656883
C	−1.126993	0.252109	−0.982950
C	−2.286380	−0.085970	−0.140956
C	−2.621175	0.968457	0.647083
H	−1.769152	3.009104	0.837192
H	−0.221059	2.311440	−1.217985
H	−3.390408	0.963745	1.400097
C	1.723564	−0.285947	1.345608
C	2.370590	−0.154572	0.063992
C	1.456387	0.126187	−0.904979
C	0.188424	0.167014	−0.254905
C	0.401185	−0.087968	1.155124
H	2.229570	−0.497981	2.270285
H	−0.373103	−0.104168	1.900670
H	−1.099680	−0.106369	−2.002390
Cl	−2.986598	−1.636529	−0.125796
H	1.643610	0.257056	−1.955440
Cl	4.053909	−0.336945	−0.164833
TS78			
0	2		
C	−1.578259	1.338789	1.602174
C	−1.080591	1.823026	0.396774
C	−1.356542	0.823433	−0.654969
C	−2.126715	−0.209782	0.058463
C	−2.218901	0.094762	1.378238
H	−1.467546	1.817751	2.559261
H	−0.698457	2.809110	0.210492
H	−2.670918	−0.527703	2.131153
C	2.318956	0.890220	−1.020396
C	2.146567	−0.160756	−0.043322
C	0.841895	−0.299168	0.282813
C	0.131121	0.675843	−0.498547
C	1.092291	1.398832	−1.288971
H	3.262962	1.190910	−1.438956
H	0.855598	2.190143	−1.979418
H	−1.681472	1.122560	−1.642194
Cl	−2.686783	−1.622259	−0.706588

Table S3. *Cont.*

TS	x	y	z
H	0.411947	−0.995920	0.977953
Cl	3.449662	−1.075273	0.583539
TS79			
0	2		
C	−2.241518	−1.471042	1.044902
C	−1.111977	−0.714877	1.259047
C	−1.396904	0.523365	−0.206887
C	−2.519375	−0.153135	−0.835228
C	−2.909961	−1.281963	−0.208140
H	−2.701499	−2.015944	1.854993
H	−0.752717	−0.495858	2.251863
H	−2.964668	0.250615	−1.730412
H	−3.702762	−1.919885	−0.561742
Cl	−1.389465	2.238729	−0.138108
C	1.602537	−1.271406	−0.908717
C	2.063359	−0.193796	−0.039150
C	1.035898	0.412776	0.556311
C	−0.202176	−0.268029	0.109710
C	0.272189	−1.317907	−0.841356
H	2.255691	−1.903818	−1.485007
H	−0.390668	−1.994146	−1.350943
H	1.071366	1.236630	1.245650
Cl	3.720808	0.188241	0.149440
TS80			
0	2		
C	1.657627	1.881852	1.019412
C	1.085503	0.676351	1.355238
C	1.477328	−0.221756	−0.319122
C	1.964057	0.927271	−1.064749
C	1.968661	2.080641	−0.365335
H	2.030777	2.545957	1.783907
H	1.147456	0.276468	2.354867
H	2.275823	0.816921	−2.091000
H	2.263895	3.030928	−0.777848
Cl	2.250063	−1.740892	−0.533390
C	−1.748914	−1.268987	0.741426
C	−2.044981	−0.060625	−0.022367
C	−0.935990	0.637028	−0.260502
C	0.193839	−0.097265	0.380015
C	−0.436041	−1.302999	0.973360
H	−2.487686	−1.989140	1.047669
H	0.113760	−2.061355	1.503281
H	−0.834054	1.567032	−0.788196
Cl	−3.638092	0.347017	−0.505253

Table S3. *Cont.*

TS	x	y	z
TS81			
0	2		
C	−1.772213	−0.480427	0.113162
C	−0.844809	−0.771433	−0.826095
C	0.484547	−0.701986	−0.190307
C	0.183370	−0.416530	1.251588
C	−1.178007	−0.250179	1.384504
H	0.916724	−0.506008	2.031988
H	−1.709264	0.030640	2.276111
C	3.261600	−0.152871	0.176718
C	2.390899	0.901874	0.223533
C	1.017783	0.718126	0.008855
C	1.534180	−1.685345	−0.537231
C	2.829301	−1.434506	−0.285360
H	4.308051	0.013049	0.376522
H	1.214332	−2.618544	−0.973117
H	3.572608	−2.188350	−0.495125
H	2.750311	1.905350	0.389330
H	−1.013099	−0.934445	−1.875291
Cl	−3.455780	−0.337953	−0.169183
Cl	0.074629	2.099010	−0.398265
TS82			
0	2		
C	−1.798142	−0.395854	0.137928
C	−0.892954	−0.846555	−0.755080
C	0.496555	−0.710971	−0.304883
C	0.171750	0.115703	1.356996
C	−1.219950	−0.016804	1.370514
H	0.740749	0.004745	2.267216
H	−1.782332	0.019542	2.288980
C	3.225950	−0.282837	0.208053
C	2.382610	0.742604	0.394943
C	0.932908	0.612204	0.172020
C	1.452920	−1.744024	−0.521201
C	2.767067	−1.556226	−0.261322
H	4.280199	−0.140320	0.390012
H	1.092211	−2.685433	−0.907437
H	3.476967	−2.350347	−0.427674
H	2.739650	1.711753	0.707042
H	−1.129264	−1.244222	−1.727853
Cl	−3.492893	−0.355942	−0.130272
Cl	0.285219	2.072462	−0.656674
TS83			
0	2		

Table S3. *Cont.*

TS	x	y	z
C	−1.642187	−1.080775	1.025454
C	−0.317684	−1.044852	1.295835
C	0.338941	−0.072589	0.394479
C	−0.756629	0.386825	−0.508226
C	−1.916964	−0.216909	−0.071476
H	0.201236	−1.575620	2.074717
C	2.121401	2.075719	−0.260811
C	2.490107	0.725824	−0.523754
C	1.690785	−0.294675	−0.161364
C	0.108616	1.356267	0.852718
C	0.994682	2.360804	0.476764
H	2.789353	2.865425	−0.563801
H	0.807065	3.369354	0.811921
H	−0.652808	1.513607	1.598340
H	3.443122	0.513991	−0.982048
H	−0.600241	0.956496	−1.404959
Cl	2.173576	−1.930036	−0.391014
H	−2.391111	−1.638338	1.561156
Cl	−3.483166	0.095992	−0.680342
TS84			
0	2		
C	−1.654065	−0.947735	1.111842
C	−0.319411	−1.086088	1.268503
C	0.479242	−0.077466	0.570258
C	−0.938285	0.906257	−0.228185
C	−1.957964	0.038624	0.128767
H	0.151702	−1.840831	1.877526
C	2.152435	2.016038	−0.306360
C	2.487562	0.649726	−0.590413
C	1.682080	−0.341647	−0.137284
C	0.075551	1.327581	0.809068
C	1.044074	2.348769	0.370693
H	2.838226	2.785058	−0.627860
H	0.841595	3.379207	0.620646
H	−0.369626	1.512410	1.784299
H	3.381608	0.412611	−1.142101
H	−0.893052	1.367016	−1.202177
Cl	2.103675	−1.991529	−0.417758
H	−2.401732	−1.559424	1.586530
Cl	−3.389325	−0.070847	−0.810371
TS85			
0	2		
C	1.578896	−0.513146	1.354132
C	0.296572	−0.580924	1.771000

Table S3. *Cont.*

TS	x	y	z
C	−0.604822	−0.621994	0.598188
C	0.332285	−0.662847	−0.573290
C	1.606133	−0.553313	−0.069616
H	0.032764	−0.892421	−1.578624
C	−2.853709	−0.124894	−1.085153
C	−1.943528	0.885543	−0.915817
C	−0.844997	0.732619	−0.062652
C	−1.800813	−1.494363	0.605703
C	−2.815561	−1.282958	−0.249421
H	−3.673951	0.007261	−1.772179
H	−1.810688	−2.318210	1.301679
H	−3.652445	−1.964348	−0.267075
H	−2.076475	1.834780	−1.410455
H	−0.056818	−0.534010	2.786502
Cl	−0.024354	2.152545	0.459272
H	2.451796	−0.401017	1.974019
Cl	3.029242	−0.413391	−1.004702
TS86			
0	2		
C	1.609987	−0.327917	1.354981
C	0.346753	−0.590424	1.759294
C	−0.670163	−0.586420	0.706472
C	0.466041	−0.170462	−0.758161
C	1.673947	−0.250550	−0.052201
H	0.410747	−0.503328	−1.782664
C	−2.811774	−0.351188	−1.099178
C	−1.864969	0.596041	−1.122028
C	−0.702439	0.565160	−0.214451
C	−1.737429	−1.532304	0.705682
C	−2.765028	−1.438458	−0.165969
H	−3.642403	−0.285465	−1.785193
H	−1.696688	−2.329707	1.432533
H	−3.560973	−2.165545	−0.155188
H	−1.923908	1.431596	−1.802219
H	0.058715	−0.766509	2.783484
Cl	−0.399882	2.210410	0.464168
H	2.469250	−0.242581	1.996539
Cl	3.141982	−0.482135	−0.897929
TS87			
0	2		
C	−1.975708	−0.079940	0.059718
C	−0.941118	0.401011	−0.668222
C	0.304856	−0.130504	−0.081024
C	−0.147973	−0.893845	1.117654

Table S3. *Cont.*

TS	x	y	z
C	−1.529900	−0.869424	1.153347
H	−0.992699	0.997233	−1.560944
H	−2.166517	−1.381205	1.852516
C	2.910212	−1.328729	−0.162810
C	2.765781	0.077090	0.016209
C	1.552668	0.659009	−0.009957
C	0.533904	−1.579559	−0.479093
C	1.822440	−2.108461	−0.478211
H	3.900043	−1.754896	−0.144605
H	1.961476	−3.136274	−0.776727
H	−0.295294	−2.097351	−0.931210
H	3.644328	0.692043	0.130406
H	0.527912	−1.245113	1.875703
Cl	1.379210	2.365745	0.106597
Cl	−3.635106	0.166354	−0.297820
TS88			
0	2		
C	−2.022168	−0.058328	0.100681
C	−0.961022	0.664117	−0.445185
C	0.256785	0.049276	−0.001456
C	−0.115509	−1.139896	0.798410
C	−1.585986	−1.104419	0.870844
H	−1.038431	1.518174	−1.092793
H	−2.202469	−1.824723	1.378167
C	2.803672	−1.419155	−0.072002
C	2.701072	−0.052010	0.264984
C	1.543816	0.647776	0.111774
C	0.420773	−1.719153	−0.471869
C	1.756535	−2.122271	−0.606141
H	3.794754	−1.845221	−0.107172
H	1.963209	−2.977998	−1.232649
H	−0.319415	−2.049964	−1.186248
H	3.607314	0.496086	0.467702
H	0.469276	−1.366453	1.681090
Cl	1.608113	2.374218	0.011743
Cl	−3.670586	0.306633	−0.200469
TS89			
0	1		
C	−3.487371	−0.675559	−0.678966
C	−2.437836	−1.531322	−0.370105
C	−1.351963	−0.775979	0.095714
C	−1.810389	0.623272	0.054507
C	−3.120090	0.639591	−0.434258
H	−4.468007	−0.989643	−0.994564

Table S3. *Cont.*

TS	x	y	z
H	−2.451651	−2.606656	−0.410322
H	−3.722885	1.522487	−0.541733
C	1.921777	−0.334255	1.352921
C	2.112618	−0.328869	−0.090552
C	1.042640	−0.822458	−0.714725
C	0.052453	−1.242516	0.327500
C	0.711769	−0.833575	1.609588
H	2.653435	0.013440	2.061601
H	0.261851	−0.977639	2.577943
H	0.012117	−2.337552	0.325449
H	−1.830469	−0.119964	1.121821
Cl	−0.790940	1.985852	0.276858
H	0.893761	−0.930478	−1.774034
Cl	3.546835	0.256267	−0.822500
TS90			
0	2		
C	−3.544472	−0.605547	−0.145288
C	−2.402065	−1.282373	−0.761003
C	−1.259930	−0.758213	−0.297261
C	−1.618833	0.315189	0.669714
C	−3.097883	0.325777	0.702250
H	−4.576048	−0.832741	−0.356320
H	−2.477741	−2.093989	−1.466851
H	−3.674232	1.014368	1.293728
C	1.941972	−1.366419	0.883364
C	2.165094	−0.289049	−0.071446
C	1.133689	−0.171651	−0.908758
C	0.131196	−1.227353	−0.564008
C	0.756786	−1.917317	0.614258
H	2.640596	−1.647184	1.652966
H	0.287480	−2.737667	1.130374
H	0.083300	−1.945117	−1.389537
H	−1.107503	0.272786	1.624805
Cl	−1.065102	2.027880	0.005122
H	1.019214	0.540887	−1.704958
Cl	3.591564	0.661474	−0.064265
H	−0.378246	3.571366	−0.509716
TS91			
0	2		
C	−2.241428	−1.669145	−0.381161
C	−1.121009	−2.223953	0.084957
C	−0.343006	−1.210660	0.877018
C	−1.191320	0.019509	0.778465
C	−2.273728	−0.280687	0.058265

Table S3. *Cont.*

TS	x	y	z
H	−3.010704	−2.134692	−0.973284
H	−0.783158	−3.235512	−0.062139
H	−0.945934	0.973940	1.208989
C	3.265266	−1.271929	−0.088829
C	2.770935	−0.549439	−1.137503
C	1.378592	−0.341933	−0.896251
C	1.045341	−1.064180	0.354876
C	2.190693	−1.585322	0.833020
H	4.291860	−1.572465	0.039363
H	3.317288	−0.166300	−1.979850
H	0.644663	−0.186319	−1.672083
H	2.302514	−2.146685	1.745268
Cl	1.502341	1.694510	−0.248638
O	0.924191	3.257886	0.655139
H	1.305746	3.104992	1.524742
H	−0.290430	−1.540759	1.918532
Cl	−3.567482	0.770615	−0.332997
TS92			
0	2		
C	−2.552953	−1.555035	−0.375259
C	−1.555203	−2.293599	0.115983
C	−0.634327	−1.423618	0.924656
C	−1.261166	−0.069124	0.816122
C	−2.360982	−0.180532	0.067144
H	−3.376479	−1.884980	−0.985349
H	−1.392799	−3.348410	−0.025038
C	2.923877	−1.969480	−0.020300
C	2.490233	−1.275673	−1.141326
C	1.148763	−0.912358	−0.919863
C	0.751621	−1.491308	0.390101
C	1.844114	−2.096232	0.913736
H	3.918394	−2.356522	0.126278
H	3.068633	−1.033607	−2.014024
H	0.432790	−0.665426	−1.686985
H	1.903521	−2.582175	1.872004
Cl	1.651008	1.143301	−0.382030
Cl	1.335056	3.219239	0.431526
H	−0.630002	−1.758248	1.964823
H	−0.860995	0.826228	1.258009
Cl	−3.446471	1.073042	−0.351594
TS93			
0	2		
C	2.465480	−0.936827	1.083537
C	1.338334	−0.189996	1.111422

Table S3. *Cont.*

TS	x	y	z
C	1.184124	0.409422	−0.196136
C	2.274405	−0.042604	−1.008304
C	3.053828	−0.846350	−0.235623
H	2.866169	−1.510862	1.901394
H	0.665957	−0.051266	1.938674
H	3.961116	−1.337375	−0.544851
C	−1.639583	1.128276	0.761484
C	−1.569220	0.034123	−0.144979
C	−0.510788	0.208303	−1.022232
C	0.182266	1.448620	−0.623298
C	−0.633714	1.990109	0.481277
H	−2.360191	1.213744	1.556825
H	−0.397881	2.894705	1.014261
H	0.550626	2.127018	−1.381591
H	−0.314915	−0.362691	−1.909652
Cl	−2.604908	−1.319286	−0.104895
H	2.441761	0.236137	−2.034733
TS94			
0	2		
C	3.382872	0.450258	0.106875
C	2.662930	−0.687112	−0.089742
C	1.291306	−0.306288	−0.244405
C	1.221795	1.135045	−0.105810
C	2.480132	1.582245	0.098746
H	4.449452	0.509309	0.244680
H	3.039331	−1.694000	−0.150765
H	0.319547	1.717674	−0.156852
H	2.773986	2.608328	0.239006
C	−1.036512	−0.564561	−1.117497
C	−1.727788	−0.199865	−0.009409
C	−1.068964	−0.619983	1.174007
C	0.083497	−1.298341	0.794856
C	0.192788	−1.244427	−0.675585
H	−1.283089	−0.327300	−2.136592
H	0.711347	−1.893279	1.431381
H	0.545919	−2.104888	−1.227832
H	−1.397223	−0.415853	2.177185
Cl	−3.179506	0.712835	−0.000731
TS95			
0	2		
C	3.111101	−0.801689	−0.163990
C	2.141288	−0.496626	−1.030289
C	1.005123	0.112872	−0.291764
C	1.467982	0.118144	1.126601

Table S3. *Cont.*

TS	x	y	z
C	2.688985	−0.420110	1.182321
H	4.057843	−1.256027	−0.405271
H	2.140980	−0.652721	−2.096415
H	0.875138	0.495320	1.941262
H	3.280423	−0.563500	2.071675
C	−0.593296	2.027678	−0.088104
C	−1.522450	1.260405	0.517703
C	−1.462211	−0.100974	0.066081
C	−0.339078	−0.529217	−0.593598
C	0.386731	1.314562	−0.880519
H	−0.561428	3.103558	−0.006615
H	−2.293154	1.620366	1.177188
H	−0.362898	−1.380668	−1.254032
H	0.696300	1.656030	−1.857003
Cl	−2.890485	−1.054861	0.080150
TS96			
0	2		
C	3.375748	0.307677	−0.051438
C	2.587494	−0.766200	0.048123
C	1.170351	−0.323620	0.068515
C	1.258476	1.161740	−0.027262
C	2.545803	1.509215	−0.102593
H	4.452629	0.303355	−0.086867
H	2.890519	−1.798524	0.106853
H	0.397762	1.807147	−0.042705
H	2.928124	2.512755	−0.191797
C	−1.042342	−0.697599	−1.103847
C	−1.696550	−0.155594	0.053874
C	−1.054938	−0.391697	1.212140
C	0.266882	−0.965931	1.032988
C	0.275588	−1.039300	−0.940549
H	−1.608917	−1.001039	−1.969930
H	0.602356	−1.820579	1.601554
H	0.754543	−1.754746	−1.590470
H	−1.467446	−0.189559	2.187371
Cl	−3.239332	0.594650	−0.067865
TS97			
0	2		
C	3.115079	−0.812051	−0.203090
C	1.963500	−1.016633	−0.889813
C	0.858902	−0.309571	−0.207421
C	1.507379	0.259690	1.010583
C	2.857345	−0.034780	0.960470
H	4.090314	−1.155856	−0.506261

Table S3. *Cont.*

TS	x	y	z
H	1.846476	−1.534893	−1.826220
H	0.950909	0.691876	1.822360
H	3.595204	0.297354	1.670577
C	−0.263223	1.933844	−0.396925
C	−1.466816	1.356860	−0.065656
C	−1.562365	−0.064215	0.002474
C	−0.502330	−0.878960	−0.132764
C	0.900626	1.178217	−0.507663
H	−0.222640	2.989562	−0.618654
H	−2.361899	1.947178	0.033740
H	−0.618397	−1.949812	−0.154002
H	1.794932	1.570362	−0.961766
Cl	−3.148440	−0.737069	0.183474
TS98			
0	2		
C	3.069659	−0.889318	−0.072402
C	1.937789	−1.230867	−0.732175
C	0.776024	−0.398210	−0.397192
C	1.607037	0.746180	0.851180
C	2.820444	0.078124	0.942815
H	4.023750	−1.367348	−0.221368
H	1.862411	−2.017660	−1.466598
H	1.110744	1.162622	1.714576
H	3.425676	0.148697	1.833373
C	−0.231130	1.873922	−0.468786
C	−1.432727	1.339754	−0.210719
C	−1.579301	−0.077935	−0.034533
C	−0.523050	−0.916040	−0.159940
C	0.995106	1.058617	−0.499695
H	−0.145992	2.931163	−0.671372
H	−2.317527	1.955522	−0.176507
H	−0.655284	−1.983705	−0.079047
H	1.735231	1.368591	−1.233836
Cl	−3.157536	−0.688426	0.293498
TS99			
0	2		
C	−2.955344	−1.170006	−0.043625
C	−1.646501	−1.555853	0.005593
C	−0.613770	−0.601164	0.029086
C	−2.333060	1.151235	−0.050587
C	−3.298103	0.198315	−0.107900
H	−3.735634	−1.914449	−0.071402
H	−1.386431	−2.603963	0.005419
H	−2.583047	2.200466	−0.095300

Table S3. *Cont.*

TS	x	y	z
H	−4.333254	0.483085	−0.214920
C	0.081424	1.749864	−0.036117
C	1.382104	1.369830	−0.076391
C	1.707922	−0.001273	−0.007079
C	0.743838	−0.964608	0.023906
C	−0.962381	0.785644	0.135097
H	−0.183474	2.794946	−0.086207
H	2.173407	2.095304	−0.172736
H	1.017575	−2.008137	0.022503
H	−0.981738	0.830453	1.794341
Cl	3.375048	−0.449977	−0.024329
TS100			
0	2		
C	2.874586	−0.899811	−0.363571
C	2.202695	0.089353	−1.001605
C	1.045941	0.504547	−0.176665
C	1.188027	−0.323419	1.060012
C	2.268962	−1.167732	0.896354
H	3.722226	−1.433706	−0.760638
H	2.400784	0.482581	−1.984075
H	0.600864	−0.158095	1.945156
H	2.579010	−1.926114	1.594629
C	−1.690343	1.229535	0.274616
C	−1.387340	−0.071559	−0.046899
C	−0.089105	−0.491098	−0.314925
C	0.610630	1.913528	−0.083941
C	−0.671264	2.223691	0.185146
H	−2.706721	1.508878	0.495400
H	1.358403	2.680350	−0.209351
H	−0.957748	3.258014	0.300588
H	0.113526	−1.442481	−0.775805
Cl	−2.660417	−1.235978	−0.186884
TS101			
0	2		
C	2.859316	−0.870887	−0.288729
C	2.342195	0.258489	−0.827005
C	1.023623	0.628608	−0.299106
C	0.802643	−0.806793	0.909536
C	2.082327	−1.338564	0.808615
H	3.799345	−1.307069	−0.583483
H	2.812659	0.846937	−1.599305
H	0.277092	−0.736271	1.849443
H	2.508310	−1.911449	1.617645
C	−0.647373	2.260597	0.325491

Table S3. *Cont.*

TS	x	y	z
C	−1.667825	1.256297	0.255490
C	−1.359179	−0.002460	−0.086273
C	0.018000	−0.454202	−0.334148
C	0.641838	1.952298	0.035604
H	−0.925670	3.268182	0.588662
H	−2.695227	1.523064	0.445908
H	0.119225	−1.180009	−1.137612
H	1.406220	2.714661	0.047676
Cl	−2.580902	−1.206961	−0.248575
TS102			
0	2		
C	−2.900270	−0.599197	−0.039795
C	−2.386119	0.663710	−0.003645
C	−0.996422	0.884920	0.017829
C	−0.691784	−1.553767	−0.031764
C	−2.037971	−1.714658	−0.090871
H	−3.968225	−0.748821	−0.067819
H	−3.043012	1.520858	−0.015296
H	−0.031270	−2.403732	−0.064728
H	−2.453652	−2.705530	−0.188502
C	0.899804	2.367600	−0.043051
C	1.774557	1.264686	−0.071734
C	1.280464	0.002025	−0.013292
C	−0.124010	−0.250860	0.142252
C	−0.451060	2.179374	−0.010823
H	1.310941	3.364088	−0.076425
H	2.838886	1.417716	−0.146463
H	−0.097067	−0.303421	1.805782
H	−1.122993	3.024332	−0.028716
Cl	2.374309	−1.331088	−0.020499
TS103			
0	2		
C	−3.362267	−0.063058	−0.201642
C	−2.423591	0.770554	−0.717742
C	−1.118117	0.478148	−0.091560
C	−1.438329	−0.578021	0.911400
C	−2.780838	−0.894286	0.794123
H	−4.390363	−0.116216	−0.520200
H	−2.554289	1.486216	−1.511492
H	−0.744958	−0.894843	1.669103
H	−3.289333	−1.668213	1.343067
C	0.879129	−0.967195	−0.626071
C	1.685971	−0.010393	−0.056359
C	1.177730	1.273460	0.299491

Table S3. *Cont.*

TS	x	y	z
C	−0.138672	1.538256	0.223474
C	−0.497093	−0.775259	−0.688563
H	1.316933	−1.849553	−1.065549
H	−0.512865	2.531433	0.414932
H	−1.123723	−1.396591	−1.306244
H	1.878932	2.044221	0.577683
Cl	3.383301	−0.280570	0.077612
TS104			
0	2		
C	−3.341900	−0.016436	−0.144415
C	−2.458756	0.919756	−0.562830
C	−1.060598	0.616438	−0.232578
C	−1.340513	−1.034727	0.646664
C	−2.725379	−0.989456	0.694910
H	−4.402090	0.025289	−0.332092
H	−2.716076	1.807066	−1.120323
H	−0.745379	−1.389839	1.474253
H	−3.270140	−1.516937	1.462622
C	0.863010	−0.929016	−0.588800
C	1.662716	0.001182	−0.050902
C	1.180139	1.269432	0.414811
C	−0.139287	1.558891	0.287526
C	−0.595327	−0.725111	−0.637464
H	1.278476	−1.836565	−0.997508
H	−0.511797	2.528496	0.583095
H	−1.082769	−1.141777	−1.515790
H	1.878663	1.981169	0.821108
Cl	3.370970	−0.263684	0.039006
TS105			
0	2		
C	3.299901	0.225380	−0.090350
C	2.321777	1.177220	−0.042007
C	0.964932	0.815118	0.019653
C	1.666188	−1.538297	−0.019608
C	2.961822	−1.145291	−0.115165
H	4.336251	0.519287	−0.148032
H	2.577822	2.226078	−0.073261
H	1.399978	−2.584200	−0.033403
H	3.742668	−1.882268	−0.221875
C	−0.746484	−0.951905	0.043541
C	−1.705400	0.004533	0.008587
C	−1.383833	1.378043	0.027424
C	−0.074822	1.761527	0.014850
C	0.627057	−0.571122	0.167336

Table S3. *Cont.*

TS	x	y	z
H	−1.006416	−1.998248	0.029456
H	0.175205	2.811559	−0.016967
H	0.684454	−0.576367	1.821603
H	−2.178588	2.105663	0.015656
Cl	−3.371659	−0.444279	−0.085809
TS106			
0	2		
C	2.619569	1.113196	0.328660
C	1.630211	1.098084	1.232346
C	0.757992	−0.069532	0.979052
C	1.349310	−0.669068	−0.243302
C	2.448180	0.007064	−0.599635
H	3.423576	1.828117	0.278204
H	1.473108	1.790298	2.041797
H	3.095382	−0.228581	−1.426380
C	−2.540024	−1.170349	0.370348
C	−2.438956	−0.020854	−0.516752
C	−1.400116	0.719185	−0.122836
C	−0.747163	0.113767	1.083237
C	−1.561193	−1.127098	1.278367
H	−3.293730	−1.935216	0.284884
H	−1.362785	−1.842898	2.058050
H	−3.092369	0.196462	−1.343854
H	−0.932130	0.775026	1.936096
Cl	−0.863449	2.180146	−0.826870
H	1.034109	−0.852037	1.858093
Cl	0.730486	−2.068276	−1.001403
H	1.208360	−1.599326	2.656853
TS107			
0	2		
C	1.886865	−2.109345	−0.111788
C	0.841545	−1.853567	−0.903879
C	0.653732	−0.373269	−1.025921
C	1.725364	0.146997	−0.116988
C	2.440050	−0.860491	0.390652
H	2.274936	−3.083818	0.133587
H	0.209924	−2.558671	−1.416305
H	3.284441	−0.764601	1.051164
C	−1.708911	2.211573	−0.347482
C	−1.784235	1.443280	0.886367
C	−1.211262	0.256664	0.667828
C	−0.747498	0.157353	−0.747643
C	−1.096928	1.487013	−1.294211
H	−2.087743	3.213609	−0.459684

Table S3. *Cont.*

TS	x	y	z
H	−2.236172	1.769793	1.806695
H	−1.482723	−0.587829	−1.266996
H	−0.882707	1.784419	−2.306231
Cl	−1.093672	−1.039219	1.777840
O	−2.328424	−1.735216	−1.514248
H	−2.465068	−2.019541	−0.600172
H	0.907916	−0.088509	−2.051663
Cl	1.982628	1.814492	0.154041
TS108			
0	2		
C	1.762139	−2.363820	−0.218410
C	0.697546	−1.905088	−0.883480
C	0.800328	−0.417621	−1.016427
C	2.050008	−0.120875	−0.247912
C	2.610470	−1.251039	0.188367
H	1.974538	−3.395914	0.004506
H	−0.125148	−2.471535	−1.286131
H	3.531517	−1.330057	0.739699
C	−1.363351	2.434164	−0.159089
C	−1.546855	1.657081	0.990823
C	−0.907849	0.453905	0.793841
C	−0.414345	0.383752	−0.615375
C	−0.628644	1.720538	−1.087692
H	−1.752774	3.427363	−0.302408
H	−1.374880	−0.149752	−1.051636
H	−0.345334	2.065297	−2.067926
Cl	−3.044289	−0.927876	−0.850693
H	0.993979	−0.190104	−2.069933
Cl	2.632510	1.474363	−0.051595
H	−2.096562	1.929824	1.873580
Cl	−0.715398	−0.748317	1.943016
TS109			
0	2		
C	−1.107174	2.311264	−0.983884
C	0.078933	1.907689	−0.481521
C	−0.135213	0.646724	0.209556
C	−1.530159	0.355780	0.080400
C	−2.126531	1.353407	−0.622512
H	−1.279389	3.196032	−1.571670
H	1.035041	2.384488	−0.596062
H	−3.176142	1.424907	−0.847449
C	2.876112	0.668412	0.450347
C	2.381660	−0.117818	−0.612739
C	1.141870	−0.646447	−0.228281

Table S3. *Cont.*

TS	x	y	z
C	0.805491	−0.118532	1.104098
C	1.991751	0.668126	1.486280
H	3.805096	1.213418	0.423893
H	2.065291	1.231359	2.400154
H	2.854233	−0.295666	−1.562206
H	0.296245	−0.748008	1.821548
Cl	0.444574	−2.012706	−0.987208
Cl	−2.318740	−0.962480	0.841403
TS110			
0	2		
C	1.626972	2.353644	0.393092
C	0.313202	2.035512	0.414242
C	0.177967	0.640220	0.067983
C	1.530818	0.158861	−0.166104
C	2.390829	1.180179	0.035273
H	2.050585	3.321073	0.600356
H	−0.516346	2.686815	0.623538
H	3.460006	1.123425	−0.067029
C	−0.945453	−2.261276	0.319936
C	−1.156136	−1.380651	−0.685640
C	−1.150278	−0.010925	−0.126829
C	−0.983151	−0.205661	1.326601
C	−0.804107	−1.564575	1.551261
H	−0.839450	−3.326331	0.202620
H	−0.581955	−2.011753	2.504579
H	−1.252399	−1.585592	−1.736681
H	−1.139128	0.564639	2.057868
Cl	−2.437466	1.040409	−0.729467
Cl	2.014801	−1.419482	−0.621365
TS111			
0	2		
C	2.035621	0.620135	1.530671
C	0.707531	0.313149	1.342025
C	0.911041	−0.524659	−0.390494
C	2.303266	−0.165599	−0.624324
C	2.880497	0.528478	0.376006
H	2.442302	0.714261	2.525884
H	0.073808	−0.017544	2.150252
H	2.784646	−0.451568	−1.545723
H	3.893426	0.893864	0.348420
Cl	0.361762	−2.047248	−0.954379
C	−1.111141	2.317667	−0.971935
C	−2.109440	1.364530	−0.505165
C	−1.474391	0.337322	0.064476

Table S3. *Cont.*

TS	x	y	z
C	−0.002532	0.554482	0.011888
C	0.112496	1.875415	−0.671966
H	−1.347392	3.239861	−1.476238
H	−3.176574	1.478103	−0.584266
H	1.057534	2.340158	−0.887617
Cl	−2.199729	−0.983496	0.866272
TS112			
0	2		
C	−0.523330	−1.748806	1.479812
C	−0.440424	−0.372085	1.515658
C	−1.147105	−0.073077	−0.251999
C	−1.184408	−1.452428	−0.707868
C	−0.781480	−2.351599	0.208159
H	−0.589486	−2.310491	2.399011
H	−0.632648	0.169800	2.428384
H	−1.508691	−1.681092	−1.709954
H	−0.705833	−3.409866	0.024195
Cl	−2.452485	0.948184	−0.725721
C	1.229520	2.481230	0.359475
C	2.112852	1.454632	−0.175099
C	1.456754	0.291773	−0.189624
C	0.069157	0.484905	0.355818
C	0.051453	1.939106	0.670882
H	1.503688	3.515382	0.481389
H	3.131727	1.601806	−0.489252
H	−0.811210	2.434683	1.079992
Cl	2.132162	−1.197721	−0.674576
TS113			
0	2		
C	0.108669	1.920737	−1.380435
C	0.705809	0.664972	−1.313439
C	−1.303608	−0.120350	−0.037753
C	−1.807286	1.124420	−0.114033
C	−1.082424	2.175851	−0.745927
H	0.577929	2.685298	−1.980167
H	1.495909	0.370964	−1.983518
H	−2.793328	1.318909	0.277060
H	−1.523265	3.158464	−0.785860
Cl	−2.214256	−1.413626	0.637756
C	1.737667	−2.029516	−0.626412
C	2.234302	−0.991661	0.200436
C	1.238940	−0.033287	0.340002
C	0.064922	−0.442320	−0.485415
C	0.461228	−1.765880	−1.009387

Table S3. *Cont.*

TS	x	y	z
H	2.309809	−2.890961	−0.928148
H	3.213341	−0.929348	0.640886
H	−0.158146	−2.347287	−1.669699
Cl	1.198281	1.155753	1.565987
TS114			
0	2		
C	0.790894	−1.672429	1.325035
C	0.854533	−0.208423	1.200865
C	−1.332744	−0.370681	0.020795
C	−1.241145	−1.726669	0.006885
C	−0.184829	−2.371378	0.726142
H	1.543464	−2.167187	1.918267
H	1.270107	0.302397	2.065536
H	−1.993117	−2.311633	−0.495373
H	−0.204718	−3.447613	0.804104
Cl	−2.674099	0.409519	−0.733165
C	0.663434	2.567475	0.491300
C	1.603183	1.753041	−0.199378
C	1.395971	0.383702	−0.084293
C	−0.353805	0.445035	0.630850
C	−0.450255	1.890471	0.856154
H	0.795388	3.628207	0.625512
H	2.320833	2.143557	−0.902340
H	−1.326899	2.330288	1.303728
Cl	1.916660	−0.681218	−1.335397
TS115			
0	2		
C	2.862849	−0.603666	−0.178029
C	2.128754	−1.779974	0.168587
C	0.802784	−1.738994	0.376785
C	0.833034	0.698010	−0.256236
C	2.209134	0.563126	−0.475072
H	3.928537	−0.668184	−0.328014
H	2.647094	−2.726209	0.194790
H	2.736387	1.390891	−0.922626
C	−1.925982	0.516541	1.085096
C	−0.898168	1.080889	1.888709
C	0.312149	0.508140	1.559041
C	0.087919	−0.446229	0.424319
C	−1.377082	−0.393482	0.248317
H	−2.965433	0.794970	1.104946
H	−1.041601	1.855896	2.621450
H	1.247518	0.577054	2.083102
H	0.222855	−2.634323	0.535515

Table S3. *Cont.*

TS	x	y	z
Cl	−0.000283	1.959047	−1.076904
Cl	−2.175464	−1.312945	−0.942994
TS116			
0	2		
C	−2.709907	1.082340	−0.041389
C	−1.762756	2.128338	0.207216
C	−0.449340	1.831579	0.327678
C	−0.923446	−0.614341	−0.023444
C	−2.329191	−0.197087	−0.166744
H	−3.753099	1.335715	−0.153122
H	−2.102361	3.148989	0.277837
H	−3.042487	−0.973930	−0.394860
C	1.833802	−0.917752	0.934561
C	0.762765	−1.416027	1.696910
C	−0.519890	−0.955037	1.369032
C	0.011488	0.488396	0.245421
C	1.440846	0.161033	0.214139
H	2.841685	−1.292340	0.964286
H	0.908928	−2.035587	2.567496
H	−1.314059	−0.969935	2.099830
H	0.286262	2.605688	0.483565
Cl	−0.490902	−1.804940	−1.310606
Cl	2.493778	1.136278	−0.714413
TS117			
0	2		
C	−2.054716	−1.217702	0.608574
C	−1.404557	−0.276110	−0.120279
C	0.050331	−0.351824	0.135195
C	0.150820	−1.409475	1.180583
C	−1.118052	−1.913651	1.414401
H	−3.108692	−1.427004	0.545815
C	2.802489	−0.412646	−0.659213
C	2.235055	0.769910	−0.103546
C	0.930422	0.832561	0.218211
C	0.659171	−1.515585	−0.620844
C	2.009902	−1.493943	−0.962017
H	3.849099	−0.414572	−0.916679
H	2.854372	1.641322	0.038007
H	2.416147	−2.325836	−1.516657
H	−1.352803	−2.729210	2.076110
H	1.051801	−1.584001	1.739511
H	−0.024606	−2.231820	−1.044120
Cl	−2.078116	0.736902	−1.312147
Cl	0.239850	2.263210	0.872830

Table S3. *Cont.*

TS	x	y	z
TS118			
0	2		
C	2.855625	0.144214	−0.619659
C	2.336674	−1.062703	−0.889976
C	0.933704	−1.377931	−0.568657
C	0.741560	1.007054	0.163161
C	2.068771	1.198108	−0.050679
H	3.889753	0.349764	−0.851031
H	2.934715	−1.825915	−1.364142
H	2.523087	2.142098	0.198935
C	−1.703601	−1.631677	0.530550
C	−0.616041	−2.187638	1.259228
C	0.646232	−1.771157	0.868024
C	0.114424	−0.232461	−0.119343
C	−1.328731	−0.510533	−0.126270
H	−2.715176	−1.998940	0.556898
H	−0.759821	−2.746343	2.170691
H	0.436445	−2.030860	−1.282163
H	1.487135	−1.769503	1.545343
Cl	−2.389668	0.456293	−1.061894
Cl	−0.203735	2.274769	0.847024
TS119			
0	2		
C	−0.331375	2.433031	−0.039677
C	0.411500	1.283474	−0.003971
C	−0.196286	0.000038	0.006925
C	−2.374731	1.216966	−0.032580
C	−1.732688	2.405174	−0.092784
H	0.189922	3.375791	−0.057853
H	−2.279916	3.329661	−0.185937
C	−1.733386	−2.404703	−0.092605
C	−2.375050	−1.216304	−0.032521
C	−1.643107	0.000213	0.130199
C	0.411119	−1.283572	−0.004140
C	−0.332047	−2.432950	−0.039522
H	−2.280898	−3.329026	−0.185721
H	0.188979	−3.375854	−0.057797
H	−1.656056	0.000385	1.797407
H	−3.451737	−1.156085	−0.060659
H	−3.451447	1.157142	−0.060405
Cl	2.120865	−1.539671	0.000360
Cl	2.121338	1.539070	0.000525
TS120			
0	2		

Table S3. *Cont.*

TS	x	y	z
C	2.315020	−0.361794	1.166372
C	1.180831	0.148365	1.671453
C	−0.073937	−0.883953	−0.256264
C	1.155134	−1.385972	−0.706421
C	2.324395	−1.084390	−0.066653
H	3.234983	−0.273319	1.723658
H	1.160316	0.626372	2.637923
H	1.148680	−2.057086	−1.550610
H	3.256024	−1.467578	−0.450607
Cl	−1.491540	−1.726966	−0.759073
C	−2.258173	0.905735	0.878062
C	−1.619592	1.410889	−0.282567
C	−0.298912	0.995329	−0.274674
C	−0.088364	0.102059	0.915465
C	−1.389600	0.169373	1.613364
H	−3.298272	1.058519	1.113243
H	−2.067104	2.015492	−1.051147
H	−1.604530	−0.391911	2.506160
Cl	0.943839	1.750240	−1.174894
TS121			
0	2		
C	2.368488	−0.774186	1.060303
C	1.147114	−0.867643	1.637396
C	0.029814	−0.377354	−0.554311
C	1.392464	−0.365077	−1.113905
C	2.476881	−0.539284	−0.346615
H	3.261724	−0.911931	1.647964
H	1.048442	−1.089216	2.689329
H	1.474490	−0.216817	−2.178524
H	3.455039	−0.517408	−0.801645
Cl	−1.076953	−1.265617	−1.670864
C	−2.319169	−0.033227	1.168924
C	−1.792927	1.064262	0.459604
C	−0.484317	0.914570	−0.027468
C	−0.047987	−0.679347	0.893326
C	−1.367763	−0.948642	1.470407
H	−3.349054	−0.099603	1.475463
H	−2.290961	2.015853	0.370279
H	−1.526591	−1.832946	2.066721
Cl	0.459972	2.341360	−0.279463

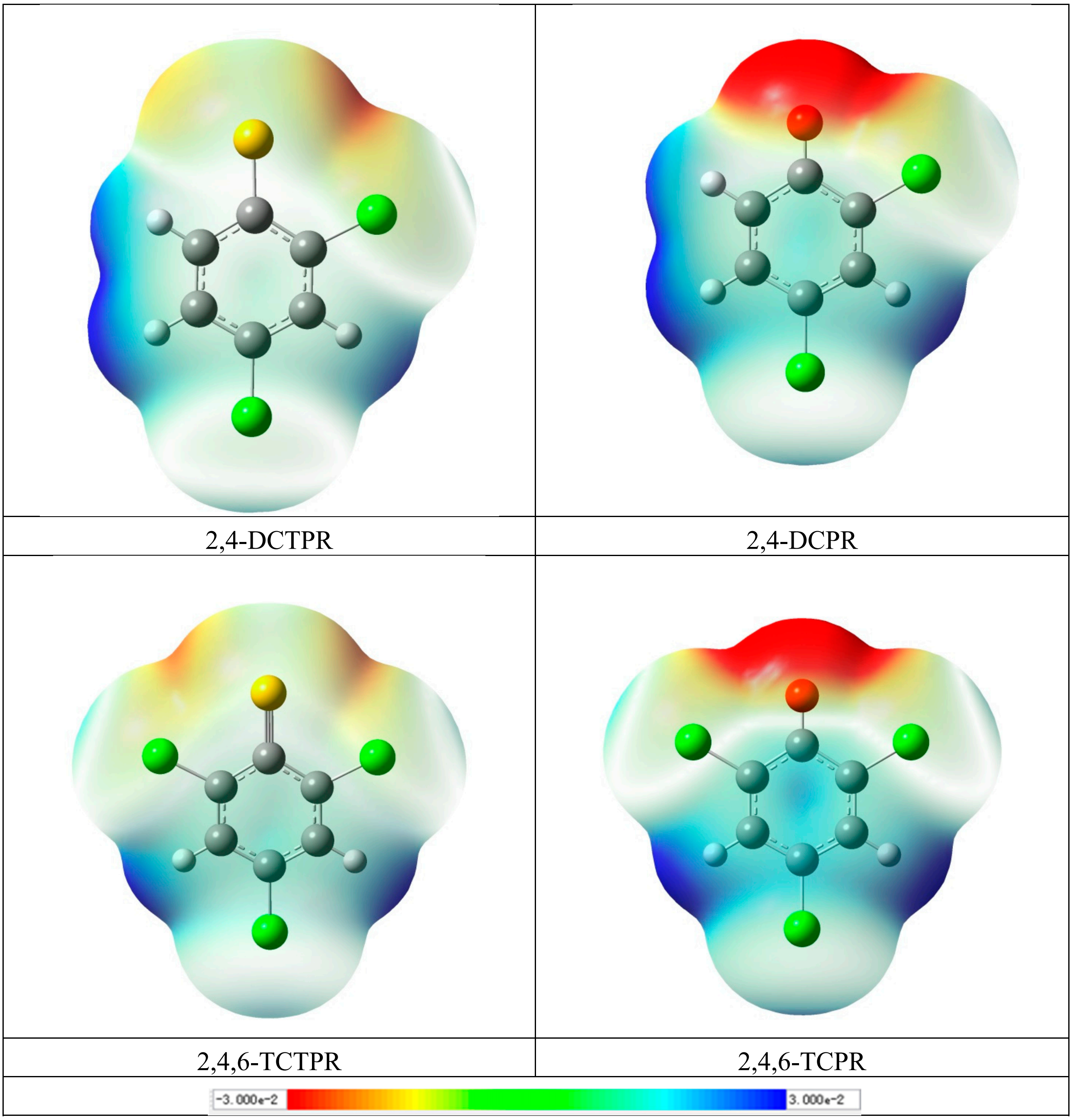

Figure S1. Electron density from total SCF density of 2,4-DCTPR, 2,4-DCPR, 2,4,6-TCTPR 2,4,6-TCPR, at MPWB1K/6-31+G(d,p) level. This is mapped on the surface of molecular electron density at 0.003 e a.u.$^{-3}$.
